# Anticancer Agent Shikonin Is an Incompetent Inducer of Cancer Drug Resistance

**DOI:** 10.1371/journal.pone.0052706

**Published:** 2013-01-03

**Authors:** Hao Wu, Jiansheng Xie, Qiangrong Pan, Beibei Wang, Danqing Hu, Xun Hu

**Affiliations:** Cancer Institute, the Second Affiliated Hospital, Zhejiang University School of Medicine, Hangzhou, Zhejiang, China; Wayne State University, United States of America

## Abstract

**Purpose:**

Cancer drug resistance is a major obstacle for the success of chemotherapy. Since most clinical anticancer drugs could induce drug resistance, it is desired to develop candidate drugs that are highly efficacious but incompetent to induce drug resistance. Numerous previous studies have proven that shikonin and its analogs not only are highly tumoricidal but also can bypass drug-transporter and apoptotic defect mediated drug resistance. The purpose of this study is to investigate if or not shikonin is a weak inducer of cancer drug resistance.

**Experimental Design:**

Different cell lines (K562, MCF-7, and a MDR cell line K562/Adr), after repeatedly treated with shikonin for 18 months, were assayed for drug resistance and gene expression profiling.

**Results:**

After 18-month treatment, cells only developed a mere 2-fold resistance to shikonin and a marginal resistance to cisplatin and paclitaxel, without cross resistance to shikonin analogs and other anticancer agents. Gene expression profiles demonstrated that cancer cells did strongly respond to shikonin treatment but failed to effectively mobilize drug resistant machineries. Shikonin-induced weak resistance was associated with the up-regulation of βII-tubulin, which physically interacted with shikonin.

**Conclusion:**

Taken together, apart from potent anticancer activity, shikonin and its analogs are weak inducers of cancer drug resistance and can circumvent cancer drug resistance. These merits make shikonin and its analogs potential candidates for cancer therapy with advantages of avoiding induction of drug resistance and bypassing existing drug resistance.

## Introduction

Cancer drug resistance is one of the major obstacles significantly interfering with the efficacy of cancer chemotherapy. Cellular factors that contribute to drug resistance include: (1) drug transporter-mediated increased efflux and decreased influx of anticancer drugs, (2) activation of DNA repair, (3) activation of detoxification system, and (4) blocked apoptosis [1], [2], [3], [4], [5], [6]. All these problems arise as a result of cancer cells' adapting to chemotherapeutic agents, i.e., the former have the capacity to attenuate the stimulation from the latter. Thus, in order to solve the problem, anticancer drugs that are toxic toward cancer cells but incompetent to induce drug resistance are desired.

**Figure 1:**
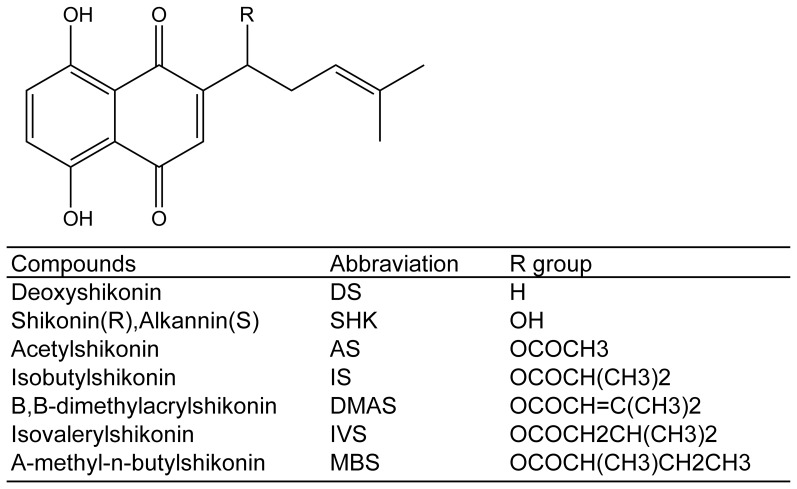
The chemical structures of shikonin and its analogues.

Shikonin is a naturally occurring naphthoquinone compound, and the main component of red pigment extracts from *Lithospermiun erythrorhizon Sieb et Zucc* of East Asia. Shikonin and its analogues are potential pharmaceutical agents with anticancer activities well documented. Shikonin and its analogues could kill cancer cells via inhibiting topoisomerase-I [7], [8], [9], polo-like kinase 1 (PLK1) and protein tyrosine kinase (PTK) [10], [11], regulating the activities of phosphorylated extracellular regulated protein kinase (pERK), c-Jun N-terminal kinase (JNK), and protein kinase C-a (PKC-a) [12], suppressing the expression of tumor necrosis factor receptor-associated protein 1 (TRAP1) [13], activating caspase activities[14], [15], inhibiting proteasome [16], among others. Sankawa *et al.* found that shikonin and a range of simple derivatives completely inhibited tumor growth in mice at a dose of 5–10 mg/kg/day [17], [18]. The LD50 of shikonin and some derivatives to mice by intraperitonal administration ranged from 20 mg/kg to 48 mg/kg [19], [20]. Notably, a clinical study indicated that shikonin mixture was effective in treatment of 19 patients with later-stage lung cancer who were not suitable for surgery, radiotherapy, and chemotherapy [21]. We reported that naturally-occurring shikonin and its analogues (Fig. 1) could circumvent cancer drug resistance mediated by drug transporters P-glycoprotein (P-gp), multidrug resistance-associated protein 1 (MRP1), and brest cancer resistance protein (BCRP1), and by antiapoptotic proteins Bcl-2 and Bcl-xL, by induction of necroptosis [22], [23], [24], [25], [26], a cell death recently defined and studied in depth by Degterev A and Yuan J [27], [28], [29]. Recently, we identified shikonin and its analogues were potent inhibitors to pyruvate kinase isozyme M2 (PKM2) or tumor M2-PK [30], which almost ubiquitously expresses in tumor cells [31] and plays important roles in cancer cell metabolism and growth [32], [33], [34]. Taken together, all these lines of evidence support that shikonin and its analogues are strong anticancer agents. However, the evidence does not mean that shikonin and its analogues are incapable of inducing drug resistance. In this study, we show that shikonin is a weak inducer of cancer drug resistance.

## Materials and Methods

### Reagents

Shikonin was purchased from National Institute for the Control of Pharmaceutical and Biological Products (Beijing, China) with purity of >99%. Doxorubicin, paclitaxel, vincristine, Methotrexate, cisplatin, dicumarol, and MTT (3-(4, 5-dimethylthiazol-2-yl)-2,5-diphenyltetrazolium bromide) were purchased from Sigma-Aldrich. Shikonin analogues (Fig. 1) were purchased from the Tokyo Chemical Industry (TCI, Tokyo).

### Cell lines

All cell lines were obtained from and characterized by The Cell Bank of Type Culture Collection of Chinese Academy of Sciences according to the cell line authentification testing (vitality, species confirmation and interspecies contamination, DNA fingerprinting and mycoplasma contamination). MCF-7 cell was maintained in DMEM containing 10% fetal bovine serum (FBS). MCF-7/Adr cell was grown in RPMI 1640 containing 10% FBS and 1 µg/mL doxorubicin. K562 was maintained in RPMI 1640 supplemented with 10% FBS. K562/Adr cell was grown in RPMI 1640 supplemented with 10% FBS and 500 ng/ml doxorubicin.

### Antibodies

The α-tubulin Rabbit polyclonal antibody was purchased from Cell Signaling Technology, Inc. (Boston, MA). Mouse monoclonal antibodies for α-tubulin, β-tubulin II and β-tubulin were purchased from Santa Cruz Biotechnology, Inc. (Santa Cruz, CA). The β-tubulin I, III and IV Mouse monoclonal antibodies were purchased from Sigma-Aldrich. Mouse anti-actin IgG was purchased from Biomedical Technologies Inc (Stoughton, MA). The secondary antibodies used are HRP-conjugated anti-mouse IgG and HRP-conjugated anti-rabbit IgG (Santa Cruz Biotechnology, CA).

### Generation of MCF-7/Shk, MCF-7/Shk-dox, K562/Shk, K562/Shk-dox and K562/Adr/Shk cell lines

MCF-7/Shk was derived by repeated exposure of MCF-7 to 4–6 µM shikonin. Briefly, MCF-7 cells (1×10^6^) were incubated with shikonin (4 µM) for 4 hours, then shikonin was removed and cells were incubated in complete DMEM medium. Cells were treated again immediately after cell growth was recovered. The total cycles of treatment were 25 with the time period of 18 months. Similarly, K562/Shk and K562/Adr/Shk were derived by repeated exposure of K562 and MDR cell line K562/Adr to shikonin.

MCF-7/Shk-dox was generated by alternative treatment of shikonin (4 µM) and doxorubicin (250 ng/mL). Briefly, cells were firstly treated with shikonin. After cell growth was recovered, cells were treated with doxorubicin. When cells resumed growth, again cells were treated with shikonin. The time period is 1.5 years with 13 cycles of treatment. Similarly, K562/Shk-dox was developed by alternative treatment of shikonin and doxorubicin.

### Cell growth inhibition assay

The drug-related effect of shikonin analogues against drug-sensitive and -resistant cells was determined by MTT assay as described [23]. Cells were seeded into 96-well plates (cultured overnight for adherent cells) and treated with shikonin analogues at serial concentrations. After a 72-hour incubation, 20 µl MTT (5 mg/ml) was added into each well for another 4-hour incubation. After that, the supernatant was removed and 150 µl dimethyl-sulphoxide (DMSO, Sigma) was added into each well in order to solubilize the blue-purple crystals of formazan. The absorbance was then measured using a model ELX800 Micro Plate Reader (Bio-Tek Instruments, Inc.) at 570 nm. The inhibition rate was calculated according to the following formula: Inhibition rate = (Absorbance of control−absorbance of treatment)/Absorbance of control×100% [35].

### Western blotting assay

Western Blotting was performed as described by us previously [22], [25]. The protein was applied to a 12% SDS polyacrylamide gel, transferred to a PVDF membrane, and then detected by the proper primary and secondary antibodies before visualization by EZ-ECL (Biological Industries, Israel). Film was scanned and densitometry was determined with Quantity One software (Bio-Rad Laboratories, CA).

### Identification of interaction between shikonin and βII-tubulin using solid-phase shikonin extraction combined with Western Blot

Solid phase shikonin was prepared according to our previous reported method [36]. The procedure to determine the interaction between βII-tubulin and solid-phase shikonin is as follows. MCF-7 cells were lysed at 4°C for 0.5 hour with M-PER Mammalian Protein Extraction Reagent (Pierce biotechnology, Rockford, IL) plus Halt Protease Inhibitor Cocktail (Pierce biotechnology, Rockford, IL), followed by centrifugation at 13,000 rpm at 4°C for 15 minutes. The clear supernatant was collected and the pellet was discarded. The total protein concentration in the supernatant was determined using a BCA protein quantification kit (Pierce biotechnology, Rockford, IL). 4 mg protein was mixed with 100 µl of shikonin-conjugated Epoxy-activated Sepharose-6B (GE Healthcare, Sweden) to a total volume of 300 µl. The mixture was incubated for 6 h at 4°C with constant and gentle stirring. The mixture was spun down at 5000 r.p.m. for 5 min at 4°C. The supernatant was discarded and the precipitate was washed by chilled washing buffer (50 mM Tris–HCl (pH 7.0), 500 mM NaCl, 10% glycerol, 1 mM DTT, 1 mM phenylmethylsulfonyl fluoride) with or without 1 mM Shikonin by centrifugation (5000 r.p.m. for 5 min at 4°C) for five times. The precipitate was mixed with SDS–PAGE sample buffer (Pierce biotechnology, Rockford, IL) and boiled for 10 min. The samples were subjected to immunoblotting analysis for βII-tubulin.

### Preparation of RNA for gene arrays

Cells were trypsinized, and total RNA was extracted using RNeasy kit (Qiagen, Germany). The quality of the total RNA was verified using a BioAnalyzer (Agilent, Santa Clara, CA). Sample preparation, labeling and hybridization to Human Genome U133 Plus 2.0 Arrays (Affymetrix, Santa Clara, CA) were performed according to manufacturer's protocols at ShanghaiBio Corporation (Shanghai, China). Raw and processed data have been deposited in Gene Expression Omnibus (GSE34298).

### Microarray data analysis

We used the Affymetrix Human U133 Plus 2.0 microarray, which interrogates over 47,000 transcripts, with an average of 27 probes per gene. Affymetrix raw data files [cell intensity (CEL) files] were first analyzed with Robust multi-array Average (RMA) normalization as implemented in the Affymetrix Expression Console Software (version 1.1) to remove between-array effects and to standardize the low-level data [37]. In order to detect differentially expressed genes, significance analysis of microarrays (SAM) algorithm [38] was used to calculate the q-values (false discovery rate) for genes in the indicated time points. SAM was performed with the software tool of The Institute for Genomic Research (TIGR) MeV (http://www.tigr.org/software/tm4/mev.html) [39]. We had three replicates for each cell lines. The list of differentially expressed genes of each indicated time point were obtained by SAM with the fold change ≥1.75, and q-values≤0.0015 compared to control.

Gene Ontology (GO) category enrichment analysis was performed on differentially expressed genes using the publicly available software DAVID (Database for Annotation, Visualization and Integrated Discovery, http://david.abcc.ncifcrf.gov/, Bethesda, MD) with default parameters [40]. The goal of this analysis was to search for GO terms in molecular function, biological process, and cellular component that were significantly enriched in the gene lists obtained above.

### Statistical analysis

Unless otherwise stated, data were expressed as the mean ± SD, and analyzed by two-tailed unpaired Student's t test.

## Results

### 1. Time frame for developing cell sublines

Repeated exposure of cancer cells to anticancer drugs would lead to development of cancer drug resistance eventually. Previously, numerous reports demonstrated that the time needed for induction of drug resistance by different drugs usually took days to months (Table S1). In this study, cancer cells were repeatedly treated with shikonin for a time period of 18 months, which was empirically sufficiently long for inducing drug resistance.

### 2. Shikonin induces a marginal resistance to shikonin, cisplatin, and paclitaxel

In general, the resistance induced by shikonin is weak. After 18-months of treatment with shikonin, MCF-7/Shk and K562/Shk showed a 2-fold resistance to shikonin but no cross-resistance to its analogues. MCF-7/Shk showed a marginal resistance to paclitaxel and cisplatin but without obvious cross-resistance to other conventional anticancer drugs (Table 1 and 2).

**Table 1:** IC_50_ profile of all the parental cells and the drug-resistant cells to shikonin analogues.

	Shikonin and analogues/µM						
Cell lines	SHK	DS	IBS	DMAS	AS	IVS	MBS
K562	0.93±0.03	1.24±0.20	0.46±0.01	0.45±0.01	0.45±0.02	0.46±0.02	0.46±0.01
K562/Shk	1.82±0.03*** (1.96)	1.03±0.05	0.47±0.01	0.46±0.01	0.54±0.08	0.46±0.01	0.47±0.03
K562/Shk-Dox	0.90±0.02	1.58±0.18	0.47±0.01	0.45±0.04	0.84±0.03*** (1.87)	0.44±0.02	0.46±0.01
K562/Adr	0.95±0.03	1.04±0.06	0.59±0.02	0.56±0.07	0.51±0.12	0.60±0.04	0.49±0.06
K562/Adr-Shk	1.96±0.05*** (2.06)	1.46±0.09** (1.40)	0.49±0.01** (0.83)	0.48±0.01	0.46±0.01	0.73±0.03*(1.22)	0.43±0.08
MCF-7	1.52±0.07	0.86±0.04	1.03±0.03	0.83±0.01	0.99±0.02	0.88±0.01	0.98±0.03
MCF-7/Shk	3.09±0.07*** (2.03)	0.88±0.05	1.17±0.07* (1.13)	0.94±0.03** (1.13)	1.20±0.12	0.94±0.04	1.21±0.07* (1.23)
MCF-7/Shk-Dox	1.72±0.82	0.69±0.04*(0.80)	0.56±0.03*** (0.54)	0.87±0.03	1.24±0.04** (1.25)	0.85±0.03	0.90±0.01* (0.92)

The inhibitory effect of shikonin analogues on drug-sensitive and -resistant cells was determined by MTT assay as described in the Material and Methods section.

* , p<0.05, statistical significance in comparison to parental cell line K562, K562/Adr or MCF-7.

Numbers in parenthesis are the drug-resistance fold (IC_50_ of drug-resistant cells/IC_50_ of the parental drug-sensitive cells).

**Table 2:** IC_50_ profile of all the parental cells and the drug-resistant cells to conventional anticancer drugs.

	Conventional anticancer drugs/nM					
Cell lines	Cisplatin	Methotrexate	Doxorubicin	Paclitaxel	Vincristine	Shikonin/µM
K562	521±16	0.98±0.03	8.76±0.54	0.92±0.02	0.59±0.02	0.93±0.03
K562/Shk	966±13*** (1.85)	0.98±0.03	9.25±0.33	0.96±0.01	0.81±0.03*** (1.37)	1.82±0.03** (1.96)
K562/Shk-Dox	473±93	0.94±0.04	57.0±9.2** (6.5)	9.42±0.58*** (10)	7.04±0.85*** (11.9)	0.90±0.02
K562/Adr	665±52	0.79±0.01	970±270*** (111)	88.7±3.2*** (96)	738±101*** (1251)	0.95±0.03
K562/Adr-Shk	1057±52** (1.59)	1.00±0.04** (1.27)	828±592	88.4±3.7	775±30	1.96±0.05** (2.06)
MCF-7	1807±33	4.61±0.66	3.48±0.47	3.87±0.71	2.78±0.48	1.52±0.07
MCF-7/Adr	n.d.	n.d.	1569±107*** (451)	84.1±19.6** (21.7)	2047±130*** (736)	n.d.
MCF-7/Shk	3308±1166 (1.83)	2.82±0.56* (0.61)	4.69±0.26* (1.35)	8.57±0.44** (2.21)	1.58±0.58	3.09±0.07** (2.03)
MCF-7/Shk-Dox	2681±109*** (1.48)	2.77±0.57* (0.60)	9.38±1.13** (2.70)	15.5±2.1** (4)	6.31±1.01* (2.27)	1.72±0.82

The inhibitory effect of conventional anticancer drugs and shikonin on drug-sensitive and -resistant cells was determined by MTT assay as described in the Material and Methods section. Numbers in parenthesis are the drug-resistance fold (IC_50_ of drug-resistant cells/IC_50_ of the parental drug-sensitive cells). n.d., not determined.

* , p<0.05;

** , p<0.01;

*** , P<0.001.

Similarly, K562/Shk only exhibited a marginal resistance to shikonin and cisplatin, without cross-resistance to other shikonin analogues and other conventional anticancer drugs (Table 1 and 2).

Overall, these results demonstrated a weak potency of shikonin in inducing cancer drug resistance.

### 3. Shikonin induces a global change of gene expression profile

We thought that the failure to induce drug resistance by shikonin could be due to the insufficient interaction between cancer cells and the compound. In order to rule out this possibility, we compared the gene expression profiles of shikonin-treated cells and control cells, which showed a global change of gene expression in shikonin-treated cells (Table S2), which encompasses all of the cellular processes as categorized by GO terms including cell cycle, cell death/survival, metabolism, organelle organization, cell motion, among others (Table S3 and S4). The results demonstrated that cancer cells did respond to shikonin but failed to effectively mobilize drug resistant machineries.

### 4. Shikonin-induced resistance is not associated with DT diaphroase

**Figure 2:**
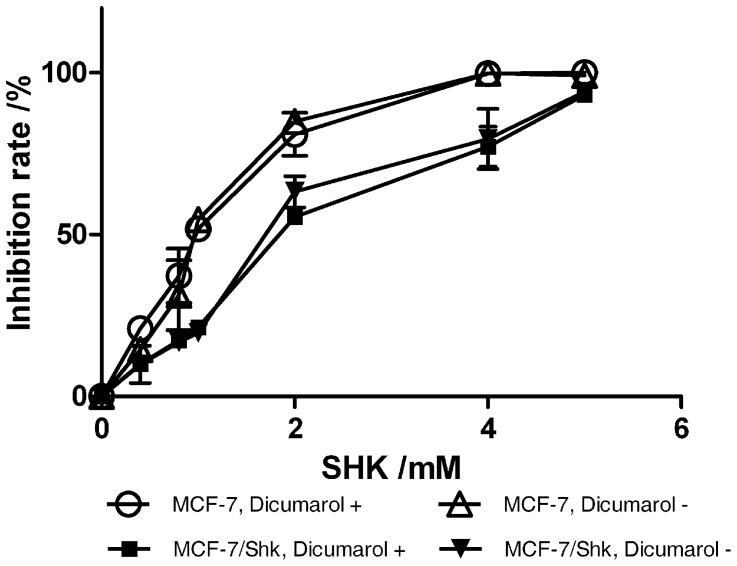
DT diaphorase is not involved in shikonin induced drug resistance. MCF-7 and MCF-7/Shk cells were treated with shikonin in the presence or absence of 20 µM dicumarol.

Shikonin is structurally similar to vitamin K3. Cancer cells can develop resistance to vitamine K3 via upregulation of DT diaphorase, which can be reversed by dicumarol, an inhibitor to the enzyme [41]. We assayed if DT diaphorase was involved in shikonin-induced drug resistance. The results demonstrated that in the presence or absence of dicumarol, there was no significant change of the sensitivity of MCF-7/Shk toward shikonin, indicating DT diaphorase did not play a major role in shikonin resistance (Fig. 2).

### 5. Shikonin-induced resistance is associated with βII-tubulin

**Figure 3:**
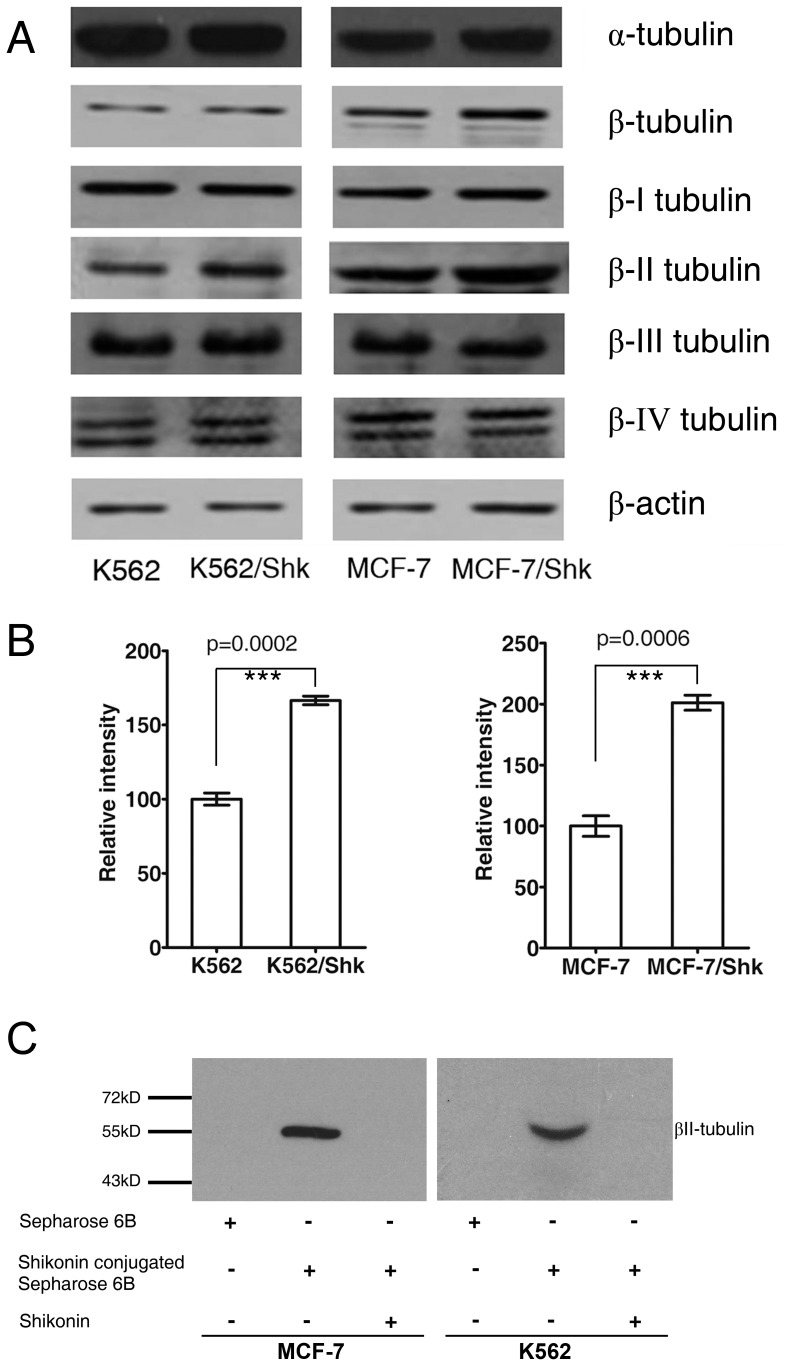
Shikonin induced resistance is associated with βII-tubulin. (A) Determination of cellular tubulins by Western blot. (B) The densitometry of βII-tubulin band in Western blot (Data are mean ± SD, n = 3). (C) Specific binding of βII-tubulin with solid-phase shikonin. MCF-7 and K562 cell lysate was incubated with Sepharose 6B or shikonin-conjugated Sepharose 6B in the absence or presence of free shikonin, followed by thorough wash. The resin is then mixed with loading buffer, boiled, and subjected for Western Blot (for details, see Materials and Methods).

Since MCF-7/Shk showed a weak cross-resistance to paclitaxel and cisplatin, and since resistance to these 2 agents could be associated with altered expression of tubulin [42], we examined tubulin expression and found that MCF-7/Shk had an increased expression of βII-tubulin (Fig. 3A&B).

Then why K562/Shk was not cross-resistant to paclitaxel, since it also showed an upregulated βII-tubulin (Fig. 3A&B). Perhaps, cells from different origins could respond to drugs differentially, e.g., MCF-7 is a breast cancer cell line whose growth depends on attachment, so that the role of tubulin is apparently more important for MCF-7 than for K562, a leukemia cell line whose growth does not depend on attachment.

The results suggested that βII-tubulin was the target by shikonin. To validate it, we used solid-phase shikonin affinity extraction of cellular protein combined with Western Blot. The results showed that βII-tubulin in MCF-7 and K562 cell lysate bound with shikonin-conjugated resin and proved the physical interaction between shikonin and βII-tubulin (Fig. 3C).

The results explained the mechanism of resistance to shikonin, but not the non-cross-resistance to its analogues. Perhaps, shikonin analogues with different R group (Fig. 1) may have lower affinity to βII-tubulin than shikonin, which can make the marginal resistance insignificant.

### 6. MDR cell line K562/Adr repeatedly exposed to shikonin develops weak resistance to this agent

K562/Adr is a cell line developed by repeated exposure of K562 to doxorubicin [43]. The cell line is characterized with overexpression of P-gp and cross resistance to anthracycline antibiotics, Vinca alkaloids, taxanes, and epipodophyllotoxins [43], [44], [45], [46], [47]. Although P-gp plays a major role in this cell line's drug resistance, given that doxorubicin kills cancer cells via producing free radicals through quinone redox cycling, inhibiting DNA Topoisomerase, intercalating into DNA helix, etc. [48], this cell line should be presumably equipped with multiple defense lines in addition to P-gp against anticancer drug. Since K562/Adr is much more adapted to drug environment than its parental cell K562, we presumed that K562/Adr could develop stronger and quicker resistance to shikonin and its analogues, if such resistance could be induced. However, after an 18-month treatment, K562/Adr/Shk, like K562/Shk, showed only a 2-fold resistance to shikonin and no cross-resistance to its analogues (Table 1), although K562/Adr/Shk kept a cross-resistance to paclitaxel, doxorubicin, and vincristine, similar to its parental cell (Table 2). These results further strengthen the notion that shikonin is an incompetent inducer of drug resistance.

### 7. Cells alternatively treated with shikonin and doxorubicin develop resistance to doxorubicin, vincristine, and paclitaxel, but not shikonin and its analogues

There are 2 possible consequences of drug-resistance induction by combined treatment of cells with shikonin and doxorubicin. First, since shikonin is incompetent to induce drug resistance, adding doxorubicin (a strong inducer of drug resistance) into drug resistance-inducing procedure would possibly help cells to acquire stronger resistance to shikonin. Conversely, since shikonin and doxorubicin kill cancer cells with distinct mechanism, the combined treatment with these 2 drugs may reciprocally reduce the magnitude of resistance.

For an 18-month alternative exposure to shikonin and doxorubicin, MCF-7/Shk-dox and K562/Shk-dox showed a cross resistance to paclitaxel, doxorubicin, and vincristine, but not to shikonin and its analogues.

The resistance to paclitaxel, doxorubicin, and vincristine was apparently induced by doxorubicin, because shikonin alone did not induce resistance to these drugs (Table 2), whereas doxorubicin had been confirmed to be a strong inducer of drug resistance with multiple mechanisms [48].

Obviously, the magnitude of resistance of MCF-7/Shk-dox and K562/Shk-dox in comparison to MCF-7/Adr and K562/Adr (2 cell lines derived by exposure of cells to doxorubicin only) was orders lower (Table 2). Cells either pulsed with high concentration or maintained in low concentration of doxorubicin alone developed fast and strong resistance [49], [50], [51], [52], [53], which could be mediated by P-gp, MRP1, DNA Topoisomerase II, among others [48], [49], [50], [51], [52], [53]. The results thus suggest that alternative treatment with shikonin and doxorubicin would reciprocally reduce the magnitude of drug resistance.

## Discussion

In summary, shikonin and likely its analogues are incompetent inducers of drug resistance. Different cell lines (K562, MCF-7, and MDR cell K562/Adr), treated with shikonin alone only developed about a 2-fold resistance to shikonin, cisplatin, and paclitaxel, without cross resistance to other shikonin analogues and anticancer drugs. Shikonin-induced resistance was associated with the up-regulation of βII-tubulin, which was a target by shikonin. Combined with our previous reports that shikonin and its analogues could circumvent drug transport and apoptosis associated cancer drug resistance [23], [24], [25], [26], [54], this class of compound deserves further attention.

The drug resistance induced by shikonin is insignificant, if known anticancer agents are used as references. In general, there are two major criteria to evaluate the capacity of a drug to induce cancer drug resistance, the time needed for the occurrence of resistance after exposure to the drug and the magnitude of resistance to the drug and cross resistance to other structurally and functionally similar or irrelevant drugs. This is routinely evaluated by exposure of cancer cell lines to tested drugs. It has been well documented that strong drug resistance can be induced by anticancer agents covering categories of platinum, Vinca alkaloids, anthracyclines, taxanes, antimetabolic agents, alkylating agents, topoisomerase inhibitors, tyrosine kinase inhibitors, and retinoids (Table S1).

One critical point is why shikonin and its analogues are incompetent inducer of cancer drug resistance. The ‘nasty’ nature of shikonin – targeting multiple important molecules – is probably a key to this solution. It has been shown that shikonin and its analogues targeted a plethora of important molecules including topoisomerase-I [7], [8], [9], PLK1, PTK [10], [11], pERK, JNK, and PKC-a [12], TRAP1 [13], caspases [14], [15], proteasome [16], PKM2 [30], among others. The broad-spectrum activity of shikonin may ‘confuse’ cancer cells to properly respond to the stimulation. Transcript profiling study provided indirect evidence to this proposed notion (Tables S2, S3, S4). Shikonin treatment did cause a global change of gene expression, but the change obviously did not contribute to the resistance, indicating that cancer cells were possibly ‘confused’ to make a decision how to properly mobilize defense machineries to the stimulation.

In summary, shikonin and its analogues have 3 merits, i.e., they are strong anticancer agents, weak inducer of cancer drug resistance, and can circumvent drug transporter and apoptotic defect mediated cancer drug resistance. The merits suggest the potential candidacy of this class of chemicals for cancer treatment.

## Supporting Information

Table S1 **Anticancer agents induce strong drug resistance.**
(PDF)

Table S2 **The list of significantly regulated genes in K562/Shk cell.** Cells were treated with shikonin for 18 months, control cells were treated with vehicle as described in Materials and Methods.
(PDF)

Table S3 **The common gene ontology terms of up regulated genes in K562/Shk cells.** Cells were treated with shikonin for 18 months, control cells were treated with vehicle as described in Materials and Methods.
(PDF)

Table S4 **The common gene ontology terms of down regulated genes in K562/Shk cells.** Cells were treated with shikonin for 18 months, control cells were treated with vehicle as described in Materials and Methods.
(PDF)
