# Supplementary material for: Anticancer Agent Shikonin Is an Incompetent Inducer of Cancer Drug Resistance
Source: PLoS One. 2013 Jan 3;8(1):e52706. doi: 10.1371/journal.pone.0052706 (PMC3536779; doi:10.1371/journal.pone.0052706)
Supplement: Table S1 — Anticancer agents induce strong drug resistance. (PDF) [file pone.0052706.s001.pdf]

Table S1. Anticancer agents induce strong drug resistance

Drugs	Cell lines	Induce time	Resisitance fold	Cross resisitance to	Possible Mechanisms	Ref.
cisplatin	CHO	At least 18 weeks	7-fold	Carboplatin (5-fold), CdSO4 (1.8-fold) and melphalan (2-fold)	Increased formation of DNA cross-links, increased glutathione 5-transferase (GST) activity.	(1)
	PC-3	At least 18 weeks	20-fold			
	A2780	6 months	17	5-fluorodeoxyuridine and 5-fluorouracil (2-fold)	Increase in both dTMP synthase activity and mRNA	(2)
	HSC-2	Over 12 months	3.65-fold	Not mentioned	Modification of oligosaccharides of A5B1 integrin	(3)
	G3361	14 months	8.7-fold	>10-fold resistant to carboplatin, 4-fold to 4-HC, and slightly (1.5-fold) to melphalan	The glutathione-S-transferase-π (GSTT) isoenzyme protein was elevated in resistant cells by 3- to 5-fold	(4)
	GCT27	15 months	5.6-fold	Carboplatin, iproplatin, chlorambucil and melphalan, and partial cross-resistance to tetraplatin, mitomycin C and methotrexate	Reduced intracellular accumulation, increased glutathione and metallothionein, and DNA binding/repair effects	(5)

vincristine	CCRF-CEM	2 years	at least 30-fold	Vinblastine, anthracyclines, adriamycin, daunorubicin, actinomycin D, VM-26 and etoposide	Increased expression of P-glycoprotein	(6)
	PC4	70 passages	215-fold	Vinblastine, etoposide, doxorubicin, and colchicine	mdr2 and mdr3 gene amplification	
	SKOV3	several weeks	1000	Vinblastin, adriamycin, colchicin, gramicidin D	Increased expression of P-glycoprotein	(7)
vinblastine	SKOV3	several weeks	10000	Vincristine, adriamycin, colchicin, gramicidin D	Increased expression of P-glycoprotein	(7)
taxol	a human ovarian carcinoma cell line, called 2008	several weeks	>1500.0	Adrumycin, camptothecin, etoposide, vincristine	Increased expression of P-glycoprotein	(8)
	MES-SA	7 days	36 to 93-fold	Doxorubicin, vinblastine, vincristine, and etoposide	Increased expression of P-glycoprotein and decreased total tublin expression.	(9)
doxorubicin	RPMI 8226	over 10 months	10 to 17-fold.	4-demethoxydaunorubicin, mitoxantrone, acronycine, vincristine, and etoposide.	Increased expression of P-glycoprotein	(10)
	HL-60	6 months	111	Daunorubicin, DHAD,vincristine, Vinblastine, Actinomycin D	Not mentioned	(11)

etoposide	KB	4, 5, 9, 6 months	29-, 145-, 186-, 287-fold	doxorubicin, m-AMSA, mitoxantrone, vincristine, methotrexate	Reduced levels of topo II, Reduced accumulation of drug	(12)
Retinoid	NB4	6 months	33-fold	doxorubicin and cisplatin	Release of cytochrome c, the activation of caspase-3, -7, -8, and -9, and deficient in the CD437-dependent activation of nuclear NFkb and AP1-binding activities and in the phosphorylation of the protein kinase Akt.	(13)
imatinib	AR230	3 months	10-fold on average	Not mentioned	Elevated expression and function of the Pgp *MDR-1* gene product	(14)
	LAMA84	3 months	10-fold on average	Not mentioned	Increased expression of P-glycoprotein	
	KCL-22	3 months	10-fold on average	Not mentioned		
	K562	3 months	10-fold on average	Not mentioned		
	32D	32-42 days		Not mentioned	Overexpression of Bcr-Abl and Abl kinase domain mutations, Glu450Gly substitution, and lacking Abl kinase domain mutations but overexpressed Bcr-Abl	(15)
	KCL-22	not mentioned	11.6-fold	Not mentioned	Upregulation of RASAP1 and RhoA gene	(16)

MTX	MOLT-3	10 months	30-fold	Dichloromethotrexate	Decreased rate of MTX transportation	(17)
	RAJI	10 months	290	trimetrexate, metoprine, homofolic acid, and CB3717	Not mentioned	(18)
	CCRF-CEM	10 months	210			
	TE-85	10 months	19			
	MG-63	10 months	8			
	SAOS-2	10 months	200	trimetrexate, metoprine, homofolic acid, and CB3717	Not mentioned	(18)
	Wl-L2/m4	10 months	13000			
5-FU	MCF-7, T47D	over 2 years	5.5 to 35.9 fold	Not mentioned	Key genes involved in 5-FU activation were significantly down-regulated (thymidine kinase, 2.9-fold; orotate phosphoribosyltransferase, 2.3-fold; uridine monophosphate kinase, 3.2-fold; pyrimidine nucleoside phosphorylase 3.6-fold) in resistant cells.	(19)
	HCT-8	six repeated 4 hour exposures and one 7-day continuous exposure	3~10	Not mentioned	Not mentioned	(20)
	SNU-769A	unavailable	3	not exhibit cross-resistance to cisplatin	Metabotropic Glutamate Receptor 4-Mediated	(21)

Camptothecin	PC-7	43 weeks	9.5	doxorubicin and cisplatin	Decreased total activity of topoisomerase I	(22)
Indolocarba-zole	SBC-3/P	20 weeks	4000-fold	CPT-11 (7.6-fold), SN-38 (22-fold) and ADM (3.24-fold)	Lower amount and decreased activity of DNA topoisomerase I	(23)
bleomycin	A-253	1 year	4, 9, 21-fold for different sublines.	peplomycin, talisomycin S100, bleomycinic acid, and X-irradiation	Decreased accumulation of bleomycin, greater DNA repair rate	(24)
bleomycin	B-16	20 passages	10-fold	2-fold to etoposide	Increased bleomycin hydrolase activity.	(25)
Cyclopho-sphamide	KHT-iv	8 cycles of exposure	3-fold	Not mentioned	GSH may "quench" the formation of cross-links following 4-OOHCP (an active form of CTX) exposure	(25)
4-hydroper-oxycyclophosphamide	G3361	14 months	10.7-fold	Not mentioned	The glutathione-S-transferase-π (GSTT) isoenzyme protein was elevated in resistant cells by 3- to 5-fold	(4)
4-hydroper-oxycyclophosphamide	Daoy	5 months	5.83-fold	Not mentioned	Elevation in GSH was accompanied by elevated levels of f-glutamyl transpeptidase.	(26)
4-hydroper-oxycyclophosphamide	D283 Med	5 months	7.9-fold	Not mentioned	Elevation in GSH was accompanied by elevated levels of f-glutamyl transpeptidase.	(26)
4-hydroper-oxycyclophosphamide	D341 Med	5 months	1.39-fold	Not mentioned	Elevation in GSH was accompanied by elevated levels of f-glutamyl transpeptidase.	(26)
carmustine	G3361	14 months	4.4-fold	Slightly (1.8-fold) resistant to melphalan.	The glutathione-S-transferase-π (GSTT) isoenzyme protein was elevated in resistant cells by 3- to 5-fold	(4)
melphalan	G3361	14 months	3.6-fold	Not mentioned	The glutathione-S-transferase-π (GSTT) isoenzyme protein was elevated in resistant cells by 3- to 5-fold	(4)

Refference

1. Saburi Y, Nakagawa M, Ono M, Sakai M, Muramatsu M, Kohno K, et al. Increased expression of glutathione S-transferase gene in cis-diamminedichloroplatinum(II)-resistant variants of a Chinese hamster ovary cell line. Cancer Res. 1989;49:7020-5.
2. Lu Y, Han J, Scanlon KJ. Biochemical and molecular properties of cisplatin-resistant A2780 cells grown in folinic acid. J Biol Chem. 1988;263:4891-4.
3. Nakahara S, Miyoshi E, Noda K, Ihara S, Gu J, Honke K, et al. Involvement of oligosaccharide changes in alpha5beta1 integrin in a cisplatin-resistant human squamous cell carcinoma cell line. Mol Cancer Ther. 2003;2:1207-14.
4. Wang YY, Teicher BA, Shea TC, Holden SA, Rosbe KW, al-Achi A, et al. Cross-resistance and glutathione-S-transferase-pi levels among four human melanoma cell lines selected for alkylating agent resistance. Cancer Res. 1989;49:6185-92.
5. Kelland LR, Mistry P, Abel G, Freidlos F, Loh SY, Roberts JJ, et al. Establishment and characterization of an in vitro model of acquired resistance to cisplatin in a human testicular nonseminomatous germ cell line. Cancer Res. 1992;52:1710-6.
6. Haber M, Norris MD, Kavallaris M, Bell DR, Davey RA, White L, et al. Atypical multidrug resistance in a therapy-induced drug-resistant human leukemia cell line (LALW-2): resistance to Vinca alkaloids independent of P-glycoprotein. Cancer Res. 1989;49:5281-7.
7. Bradley G, Naik M, Ling V. P-glycoprotein expression in multidrug-resistant human ovarian carcinoma cell lines. Cancer Res. 1989;49:2790-6.
8. Parekh H, Wiesen K, Simpkins H. Acquisition of taxol resistance via P-glycoprotein- and non-P-glycoprotein-mediated mechanisms in human ovarian carcinoma cells. Biochem Pharmacol. 1997;53:461-70.
9. Dumontet C, Duran GE, Steger KA, Beketic-Oreskovic L, Sikic BI. Resistance mechanisms in human sarcoma mutants derived by single-step exposure to paclitaxel (Taxol). Cancer Res. 1996;56:1091-7.
10. Dalton WS, Durie BG, Alberts DS, Gerlach JH, Cress AE. Characterization of a new drug-resistant human myeloma cell line that expresses P-glycoprotein. Cancer Res. 1986;46:5125-30.
11. Bhalla K, Hindenburg A, Taub RN, Grant S. Isolation and characterization of an anthracycline-resistant human leukemic cell line. Cancer Res. 1985;45:3657-62.
12. Ferguson PJ, Fisher MH, Stephenson J, Li DH, Zhou BS, Cheng YC. Combined modalities of resistance in etoposide-resistant human KB cell lines. Cancer Res. 1988;48:5956-64.
13. Ponzanelli I, Gianni M, Giavazzi R, Garofalo A, Nicoletti I, Reichert U, et al. Isolation and characterization of an acute promyelocytic leukemia cell line selectively resistant to the novel antileukemic and apoptogenic retinoid 6-[3-adamantyl-4-hydroxyphenyl]-2-naphthalene carboxylic acid. Blood. 2000;95:2672-82.
14. Mahon FX, Deininger MW, Schultheis B, Chabrol J, Reiffers J, Goldman JM, et al. Selection and characterization of BCR-ABL positive cell lines with differential sensitivity to the tyrosine kinase inhibitor STI571: diverse mechanisms of resistance. Blood. 2000;96:1070-9.
15. Barnes DJ, Palaiologou D, Panousopoulou E, Schultheis B, Yong AS, Wong A, et al. Bcr-Abl expression levels determine the rate of development of resistance to imatinib mesylate in chronic myeloid leukemia. Cancer Res. 2005;65:8912-9.
16. Ohmine K, Nagai T, Tarumoto T, Miyoshi T, Muroi K, Mano H, et al. Analysis of gene

expression profiles in an imatinib-resistant cell line, KCL22/SR. Stem Cells. 2003;21:315-21.
17. Ohnoshi T, Ohnuma T, Takahashi I, Scanlon K, Kamen BA, Holland JF. Establishment of methotrexate-resistant human acute lymphoblastic leukemia cells in culture and effects of folate antagonists. Cancer Res. 1982;42:1655-60.
18. Diddens H, Niethammer D, Jackson RC. Patterns of cross-resistance to the antifolate drugs trimetrexate, metoprine, homofolate, and CB3717 in human lymphoma and osteosarcoma cells resistant to methotrexate. Cancer Res. 1983;43:5286-92.
19. Wang W, Cassidy J, O'Brien V, Ryan KM, Collie-Duguid E. Mechanistic and predictive profiling of 5-Fluorouracil resistance in human cancer cells. Cancer Res. 2004;64:8167-76.
20. Sobrero AF, Aschele C, Guglielmi AP, Mori AM, Melioli GG, Rosso R, et al. Synergism and lack of cross-resistance between short-term and continuous exposure to fluorouracil in human colon adenocarcinoma cells. J Natl Cancer Inst. 1993;85:1937-44.
21. Yoo BC, Jeon E, Hong SH, Shin YK, Chang HJ, Park JG. Metabotropic glutamate receptor 4-mediated 5-Fluorouracil resistance in a human colon cancer cell line. Clin Cancer Res. 2004;10:4176-84.
22. Kanzawa F, Sugimoto Y, Minato K, Kasahara K, Bungo M, Nakagawa K, et al. Establishment of a camptothecin analogue (CPT-11)-resistant cell line of human non-small cell lung cancer: characterization and mechanism of resistance. Cancer Res. 1990;50:5919-24.
23. Kanzawa F, Nishio K, Kubota N, Saijo N. Antitumor activities of a new indolocarbazole substance, NB-506, and establishment of NB-506-resistant cell lines, SBC-3/NB. Cancer Res. 1995;55:2806-13.
24. Lazo JS, Braun ID, Labaree DC, Schisselbauer JC, Meandzija B, Newman RA, et al. Characteristics of bleomycin-resistant phenotypes of human cell sublines and circumvention of bleomycin resistance by liblomycin. Cancer Res. 1989;49:185-90.
25. Zuckerman JE, Raffin TA, Brown JM, Newman RA, Etiz BB, Sikic BI. In vitro selection and characterization of a bleomycin-resistant subline of B16 melanoma. Cancer Res. 1986;46:1748-53.
26. Friedman HS, Colvin OM, Kaufmann SH, Ludeman SM, Bullock N, Bigner DD, et al. Cyclophosphamide resistance in medulloblastoma. Cancer Res. 1992;52:5373-8.
