# Supplementary material for: Anticancer Agent Shikonin Is an Incompetent Inducer of Cancer Drug Resistance
Source: PLoS One. 2013 Jan 3;8(1):e52706. doi: 10.1371/journal.pone.0052706 (PMC3536779; doi:10.1371/journal.pone.0052706)
Supplement: Table S2 — The list of significantly regulated genes in K562/Shk cell. Cells were treated with shikonin for 18 months, control cells were treated with vehicle as described in Materials and Methods. (PDF) [file pone.0052706.s002.pdf]

Table S2. The list of significantly regulated genes in K562/Shk cell. Cells were treated with shikonin for 18 months, control cells were treated with vehicle as described in Materials and Methods

Probe Set ID	Gene Symbol	Gene Title	RefSeq Transcript ID	Score(d)	Numerat-or(r)	Denominat or(s+s0)	Fold Change	q-value(%)
Up reglulated								
232010_at	FSTL5	follistatin-like 5	NM_001128427 /// NM_001128428 /// NM_020116	17.2248	5.1119	0.2968	34.5804	0.0000
241133_at	TRBV27	T cell receptor beta variable 27	NA	6.3858	4.5695	0.7156	23.7436	0.0000
1565162_s_at	MGST1	microsomal glutathione S-transferase 1	NM_020300 /// NM_145764 /// NM_145791 /// NM_145792	21.1350	4.1979	0.1986	18.3527	0.0000
223168_at	RHOU	ras homolog gene family, member U	NM_021205	12.2198	4.0156	0.3286	16.1742	0.0000
224918_x_at	MGST1	microsomal glutathione S-transferase 1	NM_020300 /// NM_145764 /// NM_145791 /// NM_145792	21.3690	3.8939	0.1822	14.8654	0.0000
231736_x_at	MGST1	microsomal glutathione S-transferase 1	NM_020300 /// NM_145764 /// NM_145791 /// NM_145792	20.6080	3.7643	0.1827	13.5882	0.0000
206291_at	NTS	neurotensin	NM_006183	9.3340	3.5816	0.3837	11.9720	0.0000
213558_at	PCLO	piccolo (presynaptic cytomatrix protein)	NM_014510 /// NM_033026	14.9682	3.5148	0.2348	11.4302	0.0000
214023_x_at	TUBB2B	tubulin, beta 2B	NM_178012	7.2443	3.4547	0.4769	10.9641	0.0000
212865_s_at	COL14A1	collagen, type XIV, alpha 1	NM_021110	15.7276	3.4217	0.2176	10.7160	0.0000
1552950_at	C15orf26	chromosome 15 open reading frame 26	NM_173528	5.4581	3.3851	0.6202	10.4476	0.0000

Table S2. Continued

Probe Set ID	Gene Symbol	Gene Title	RefSeq Transcript ID	Score(d)	Numerat-or(r)	Denominator(s+s0)	Fold Change	q-value(%)
Up reglulated								
207638_at	TMPRSS15	transmembrane protease, serine 15	NM_002772	14.0754	3.2775	0.2329	9.6966	0.0000
212158_at	SDC2	syndecan 2	NM_002998	10.7393	2.9963	0.2790	7.9795	0.0000
209160_at	AKR1C3	aldo-keto reductase family 1, member C3 (3-alpha hydroxysteroid dehydrogenase, type II)	NM_003739	12.7426	2.9496	0.2315	7.7253	0.0000
228554_at	PGR	progesterone receptor	NM_000926	12.7556	2.9340	0.2300	7.6421	0.0000
212154_at	SDC2	syndecan 2	NM_002998	9.3582	2.9292	0.3130	7.6168	0.0000
223595_at	TMEM133	transmembrane protein 133	NM_032021	11.1942	2.9129	0.2602	7.5313	0.0000
231202_at	ALDH1L2	aldehyde dehydrogenase 1 family, member L2	NM_001034173 /// NR_027752	3.6472	2.8891	0.7921	7.4080	0.0000
233822_x_at	NA	NA	NA	13.6628	2.8744	0.2104	7.3328	0.0000
223655_at	CD163L1	CD163 molecule-like 1	NM_174941	11.1665	2.8723	0.2572	7.3221	0.0000
203849_s_at	KIF1A	kinesin family member 1A	NM_004321	4.3375	2.8601	0.6594	7.2608	0.0000
227238_at	MUC15	mucin 15, cell surface associated	NM_001135091 /// NM_001135092 /// NM_145650	11.9732	2.8304	0.2364	7.1126	0.0000
223058_at	FAM107B	family with sequence similarity 107, member B	NM_031453	9.1325	2.8254	0.3094	7.0883	0.0000
225330_at	IGF1R	insulin-like growth factor 1 receptor	NM_000875	9.5475	2.7691	0.2900	6.8166	0.0000
1552943_at	GABRG1	gamma-aminobutyric acid (GABA) A receptor, gamma 1	NM_173536	8.1343	2.7149	0.3338	6.5653	0.0000
223059_s_at	FAM107B	family with sequence similarity 107, member B	NM_031453	13.4801	2.6970	0.2001	6.4844	0.0000

Table S2. Continued

Probe Set ID	Gene Symbol	Gene Title	RefSeq Transcript ID	Score(d)	Numerat-or(r)	Denominator(s+s0)	Fold Change	q-value(%)
Up reglulated								
203735_x_at	PPFIBP1	PTPRF interacting protein, binding protein 1 (liprin beta 1)	NM_003622 /// NM_177444	7.2305	2.6640	0.3684	6.3380	0.0000
1561331_at	C1orf99	chromosome 1 open reading frame 99	NM_001012274	13.9966	2.6555	0.1897	6.3005	0.0000
227241_at	MUC15	mucin 15, cell surface associated	NM_001135091 /// NM_001135092 /// NM_145650	9.6369	2.6495	0.2749	6.2745	0.0000
230047_at	ARHGAP42	Rho GTPase activating protein 42	NM_152432	13.0919	2.6024	0.1988	6.0729	0.0000
225093_at	UTRN	utrophin	NM_007124	10.2595	2.5675	0.2503	5.9278	0.0000
202404_s_at	COL1A2	collagen, type I, alpha 2	NM_000089	8.7507	2.5644	0.2931	5.9152	0.0000
203628_at	IGF1R	insulin-like growth factor 1 receptor	NM_000875	13.1993	2.5585	0.1938	5.8909	0.0000
210517_s_at	AKAP12	A kinase (PRKA) anchor protein 12	NM_005100 /// NM_144497	8.4575	2.4680	0.2918	5.5326	0.0000
210116_at	SH2D1A	SH2 domain containing 1A	NM_001114937 /// NM_002351	5.8983	2.3830	0.4040	5.2162	0.0000
202336_s_at	PAM	peptidylglycine alpha-amidating monooxygenase	NM_000919 /// NM_001177306 /// NM_138766 /// NM_138821 /// NM_138822 /// NR_033440	11.0275	2.3745	0.2153	5.1854	0.0000
230748_at	SLC16A6	solute carrier family 16, member 6 (monocarboxylic acid transporter 7)	NM_001174166 /// NM_004694	7.7961	2.3438	0.3006	5.0764	0.0000

Table S2. Continued

Probe Set ID	Gene Symbol	Gene Title	RefSeq Transcript ID	Score(d)	Numerat-or(r)	Denominat or(s+s0)	Fold Change	q-value(%)
Up reglulated								
203736_s_at	PPFIBP1	PTPRF interacting protein, binding protein 1 (liprin beta 1)	NM_003622 /// NM_177444	7.2546	2.3354	0.3219	5.0469	0.0000
209529_at	PPAP2C	phosphatidic acid phosphatase type 2C	NM_003712 /// NM_177526 /// NM_177543	7.2070	2.3300	0.3233	5.0282	0.0000
225185_at	MRAS	muscle RAS oncogene homolog	NM_001085049 /// NM_012219	10.2391	2.2817	0.2228	4.8626	0.0000
227178_at	CELF2	CUGBP, Elav-like family member 2	NM_001025076 /// NM_001025077 /// NM_001083591 /// NM_006561	7.6771	2.2084	0.2877	4.6216	0.0000
209890_at	TSPAN5	tetraspanin 5	NM_005723	5.2812	2.1992	0.4164	4.5923	0.0000
209343_at	EFHD1	EF-hand domain family, member D1	NM_025202 /// NR_027663	8.6817	2.1806	0.2512	4.5334	0.0000
203627_at	IGF1R	insulin-like growth factor 1 receptor	NM_000875	7.1302	2.1637	0.3035	4.4807	0.0000
225387_at	TSPAN5	tetraspanin 5	NM_005723	6.5519	2.1631	0.3301	4.4788	0.0000
233847_x_at	NA	NA	NA	4.8902	2.1624	0.4422	4.4767	0.0000
202660_at	ITPR2	inositol 1,4,5-triphosphate receptor, type 2	NM_002223	7.0722	2.1592	0.3053	4.4667	0.0000
229830_at	PDGFA	Platelet-derived growth factor alpha polypeptide	NM_002607 /// NM_033023	10.6061	2.1371	0.2015	4.3989	0.0000
229070_at	C6orf105	chromosome 6 open reading frame 105	NM_001143948 /// NM_032744	4.7310	2.1068	0.4453	4.3075	0.0000

Table S2. Continued

Probe Set ID	Gene Symbol	Gene Title	RefSeq Transcript ID	Score(d)	Numerat-or(r)	Denominator(s+s0)	Fold Change	q-value(%)
Up reglulated								
204718_at	EPHB6	EPH receptor B6	NM_004445	4.3895	2.0733	0.4723	4.2084	0.0000
1557080_s_at	ITGBL1	integrin, beta-like 1 (with EGF-like repeat domains)	NM_004791	7.3813	2.0625	0.2794	4.1772	0.0000
227135_at	NAAA	N-acylethanolamine acid amidase	NM_001042402 /// NM_014435	9.5232	2.0582	0.2161	4.1645	0.0000
224354_at	NA	NA	NA	6.9492	2.0447	0.2942	4.1258	0.0000
1570561_at	NA	NA	NA	4.5879	2.0424	0.4452	4.1194	0.0000
212771_at	FAM171A1	family with sequence similarity 171, member A1	NM_001010924	7.9260	2.0183	0.2546	4.0512	0.0000
202156_s_at	CELF2	CUGBP, Elav-like family member 2	NM_001025076 /// NM_001025077 /// NM_001083591 /// NM_006561	12.4744	2.0120	0.1613	4.0335	0.0000
222857_s_at	KCNMB4	potassium large conductance calcium-activated channel, subfamily M, beta member 4	NM_014505	8.9814	2.0040	0.2231	4.0110	0.0000
1555854_at	AKR1C1 /// AKR1C2	aldo-keto reductase family 1, member C1 (dihydrodiol dehydrogenase 1; 20-alpha (3-alpha)-hydroxysteroid dehydrogenase) /// aldo-keto reductase family 1, member C2 (dihydrodiol dehydrogenase 2; bile acid binding protein; 3-alpha hydroxysteroid dehydrogenas	NM_001135241 /// NM_001353 /// NM_001354 /// NM_205845	6.6473	1.9896	0.2993	3.9714	0.0000

Table S2. Continued

Probe Set ID	Gene Symbol	Gene Title	RefSeq Transcript ID	Score(d)	Numerat-or(r)	Denominator(s+s0)	Fold Change	q-value(%)
Up reglulated								
212958_x_at	PAM	peptidylglycine alpha-amidating monooxygenase	NM_000919 /// NM_001177306 /// NM_138766 /// NM_138821 /// NM_138822 /// NR_033440	8.5372	1.9857	0.2326	3.9606	0.0000
211208_s_at	CASK	calcium/calmodulin-dependent serine protein kinase (MAGUK family)	NM_001126054 /// NM_001126055 /// NM_003688	7.5349	1.9741	0.2620	3.9288	0.0000
1563063_at	NA	NA	NA	7.7518	1.9647	0.2535	3.9034	0.0000
204105_s_at	NRCAM	neuronal cell adhesion molecule	NM_001037132 /// NM_001193582 /// NM_001193583 /// NM_001193584 /// NM_005010	8.8602	1.9588	0.2211	3.8874	0.0000
231993_at	ITGBL1	Integrin, beta-like 1 (with EGF-like repeat domains)	NM_004791	6.2923	1.9587	0.3113	3.8871	0.0000
230480_at	PIWIL4	piwi-like 4 (Drosophila)	NM_152431	9.3139	1.9541	0.2098	3.8748	0.0000
224480_s_at	AGPAT9	1-acylglycerol-3-phosphate O-acyltransferase 9	NM_032717	10.6983	1.9493	0.1822	3.8619	0.0000
205728_at	ODZ1	odz, odd Oz/ten-m homolog 1(Drosophila)	NM_001163278 /// NM_001163279 /// NM_014253	3.1322	1.9164	0.6118	3.7748	0.0852

Table S2. Continued

Probe Set ID	Gene Symbol	Gene Title	RefSeq Transcript ID	Score(d)	Numerat-or(r)	Denominator(s+s0)	Fold Change	q-value(%)
Up reglulated								
219882_at	TTLL7	tubulin tyrosine ligase-like family, member 7	NM_024686	11.9201	1.9163	0.1608	3.7745	0.0000
1561775_at	NA	NA	NA	7.4353	1.9073	0.2565	3.7511	0.0000
231270_at	CA13	carbonic anhydrase XIII	NM_198584	7.4079	1.9047	0.2571	3.7443	0.0000
217764_s_at	RAB31	RAB31, member RAS oncogene family	NM_006868	8.3472	1.9013	0.2278	3.7356	0.0000
208305_at	PGR	progesterone receptor	NM_000926	5.1783	1.8909	0.3652	3.7087	0.0000
217763_s_at	RAB31	RAB31, member RAS oncogene family	NM_006868	7.8728	1.8880	0.2398	3.7013	0.0000
210002_at	GATA6	GATA binding protein 6	NM_005257	5.9149	1.8840	0.3185	3.6910	0.0000
202158_s_at	CELF2	CUGBP, Elav-like family member 2	NM_001025076 /// NM_001025077 /// NM_001083591 /// NM_006561	10.0988	1.8779	0.1860	3.6754	0.0000
202403_s_at	COL1A2	collagen, type I, alpha 2	NM_000089	7.3423	1.8511	0.2521	3.6077	0.0000
224928_at	SETD7	SET domain containing (lysine methyltransferase) 7	NM_030648	9.9267	1.8423	0.1856	3.5858	0.0000
205767_at	EREG	epiregulin	NM_001432	3.9392	1.8365	0.4662	3.5715	0.0000
1555623_at	NA	NA	NA	5.5237	1.8310	0.3315	3.5579	0.0000
228523_at	NANOS1	nanos homolog 1 (Drosophila)	NM_199461	4.5993	1.8237	0.3965	3.5398	0.0000
212157_at	SDC2	syndecan 2	NM_002998	6.7701	1.8223	0.2692	3.5364	0.0000
226069_at	PRICKLE1	prickle homolog 1 (Drosophila)	NM_001144881 /// NM_001144882 /// NM_001144883 /// NM_153026	8.9514	1.8210	0.2034	3.5332	0.0000

Table S2. Continued

Probe Set ID	Gene Symbol	Gene Title	RefSeq Transcript ID	Score(d)	Numerat-or(r)	Denominator(s+s0)	Fold Change	q-value(%)
Up reglulated								
212614_at	ARID5B	AT rich interactive domain 5B (MRF1-like)	NM_032199	5.6005	1.8179	0.3246	3.5257	0.0000
203335_at	PHYH	phytanoyl-CoA 2-hydroxylase	NM_001037537 /// NM_006214	12.9282	1.8167	0.1405	3.5228	0.0000
214581_x_at	TNFRSF21	tumor necrosis factor receptor superfamily, member 21	NM_014452	13.0268	1.8163	0.1394	3.5218	0.0000
225482_at	KIF1A	kinesin family member 1A	NM_004321	7.6755	1.8104	0.2359	3.5074	0.0000
228750_at	NA	NA	NA	10.4783	1.8071	0.1725	3.4993	0.0000
226065_at	PRICKLE1	prickle homolog 1 (Drosophila)	NM_001144881 /// NM_001144882 /// NM_001144883 /// NM_153026	10.8675	1.8047	0.1661	3.4936	0.0000
228656_at	PROX1	prospero homeobox 1	NM_002763	9.7875	1.8000	0.1839	3.4821	0.0000
217762_s_at	RAB31	RAB31, member RAS oncogene family	NM_006868	7.9929	1.7937	0.2244	3.4671	0.0000
214927_at	ITGBL1	integrin, beta-like 1 (with EGF-like repeat domains)	NM_004791	9.7881	1.7866	0.1825	3.4500	0.0000
1556321_a_at	NA	NA	NA	6.0426	1.7855	0.2955	3.4473	0.0000
227197_at	ARHGEF26	Rho guanine nucleotide exchange factor (GEF) 26	NM_015595	4.1652	1.7822	0.4279	3.4395	0.0000
210102_at	VWA5A	von Willebrand factor A domain containing 5A	NM_001130142 /// NM_014622 /// NM_198315	5.8077	1.7764	0.3059	3.4258	0.0000

Table S2. Continued

Probe Set ID	Gene Symbol	Gene Title	RefSeq Transcript ID	Score(d)	Numerat-or(r)	Denominat or(s+s0)	Fold Change	q-value(%)
Up reglulated								
211653_x_at	AKR1C2	aldo-keto reductase family 1, member C2 (dihydrodiol dehydrogenase 2; bile acid binding protein; 3-alpha hydroxysteroid dehydrogenase, type III)	NM_001135241 /// NM_001354 /// NM_205845	8.0838	1.7708	0.2190	3.4123	0.0000
208712_at	CCND1	cyclin D1	NM_053056	5.9622	1.7673	0.2964	3.4042	0.0000
227404_s_at	EGR1	Early growth response 1	NM_001964	3.1182	1.7589	0.5641	3.3843	0.0852
227966_s_at	CCDC74A /// CCDC74B	coiled-coil domain containing 74A /// coiled-coil domain containing 74B	NM_138770 /// NM_207310	5.9566	1.7578	0.2951	3.3818	0.0000
234985_at	LDLRAD3	low density lipoprotein receptor class A domain containing 3	NM_174902	9.8192	1.7453	0.1777	3.3525	0.0000
209699_x_at	AKR1C2	aldo-keto reductase family 1, member C2 (dihydrodiol dehydrogenase 2; bile acid binding protein; 3-alpha hydroxysteroid dehydrogenase, type III)	NM_001135241 /// NM_001354 /// NM_205845	6.8067	1.7422	0.2560	3.3455	0.0000
202620_s_at	PLOD2	procollagen-lysine, 2-oxoglutarate 5-dioxygenase 2	NM_000935 /// NM_182943	10.9597	1.7378	0.1586	3.3353	0.0000
209189_at	FOS	FBJ murine osteosarcoma viral oncogene homolog	NM_005252	2.7741	1.7378	0.6264	3.3352	0.1046
204151_x_at	AKR1C1	aldo-keto reductase family 1, member C1 (dihydrodiol dehydrogenase 1; 20-alpha (3-alpha)-hydroxysteroid dehydrogenase)	NM_001353	6.6964	1.7285	0.2581	3.3138	0.0000
209803_s_at	PHLDA2	pleckstrin homology-like domain, family A, member 2	NM_003311	4.4940	1.7266	0.3842	3.3095	0.0000

Table S2. Continued

Probe Set ID	Gene Symbol	Gene Title	RefSeq Transcript ID	Score(d)	Numerat-or(r)	Denominator(s+s0)	Fold Change	q-value(%)
Up reglulated								
228194_s_at	SORCS1	sortilin-related VPS10 domain containing receptor 1	NM_001013031 /// NM_052918	5.2884	1.7265	0.3265	3.3093	0.0000
205659_at	HDAC9	histone deacetylase 9	NM_014707 /// NM_058176 /// NM_178423 /// NM_178425	6.7789	1.7253	0.2545	3.3064	0.0000
205304_s_at	KCNJ8	potassium inwardly-rectifying channel, subfamily J, member 8	NM_004982	11.6995	1.7247	0.1474	3.3052	0.0000
230763_at	SPATA17	spermatogenesis associated 17	NM_138796	5.0630	1.7157	0.3389	3.2845	0.0000
203562_at	FEZ1	fasciculation and elongation protein zeta 1 (zygin I)	NM_005103 /// NM_022549	5.3790	1.7084	0.3176	3.2680	0.0000
202429_s_at	PPP3CA	protein phosphatase 3, catalytic subunit, alpha isozyme	NM_000944 /// NM_001130691 /// NM_001130692	11.7555	1.7064	0.1452	3.2635	0.0000
212651_at	RHOBTB1	Rho-related BTB domain containing 1	NM_014836 /// NR_024554 /// NR_024555 /// NR_024556	8.7843	1.6984	0.1933	3.2453	0.0000
210650_s_at	PCLO	piccolo (presynaptic cytomatrix protein)	NM_014510 /// NM_033026	4.9404	1.6925	0.3426	3.2321	0.0000
225388_at	TSPAN5	tetraspanin 5	NM_005723	5.3462	1.6908	0.3163	3.2283	0.0000
229606_at	NA	NA	NA	10.8102	1.6901	0.1563	3.2269	0.0000
205191_at	RP2	retinitis pigmentosa 2 (X-linked recessive)	NM_006915	9.0024	1.6821	0.1868	3.2089	0.0000

Table S2. Continued

Probe Set ID	Gene Symbol	Gene Title	RefSeq Transcript ID	Score(d)	Numerat-or(r)	Denominat or(s+s0)	Fold Change	q-value(%)
Up reglulated								
202619_s_at	PLOD2	procollagen-lysine, 2-oxoglutarate 5-dioxygenase 2	NM_000935 /// NM_182943	7.2739	1.6789	0.2308	3.2018	0.0000
200636_s_at	PTPRF	protein tyrosine phosphatase, receptor type, F	NM_002840 /// NM_130440	5.2051	1.6720	0.3212	3.1865	0.0000
205406_s_at	SPA17	sperm autoantigenic protein 17	NM_017425	6.4653	1.6662	0.2577	3.1737	0.0000
1562102_at	AKR1C1	Aldo-keto reductase family 1, member C1 (dihydrodiol dehydrogenase 1; 20-alpha (3-alpha)-hydroxysteroid dehydrogenase)	NM_001353	5.7043	1.6631	0.2916	3.1671	0.0000
201468_s_at	NQO1	NAD(P)H dehydrogenase, quinone 1	NM_000903 /// NM_001025433 /// NM_001025434	6.8811	1.6521	0.2401	3.1428	0.0000
205801_s_at	RASGRP3	RAS guanyl releasing protein 3 (calcium and DAG-regulated)	NM_001139488 /// NM_015376 /// NM_170672	5.5889	1.6516	0.2955	3.1418	0.0000
223502_s_at	TNFSF13B	tumor necrosis factor (ligand) superfamily, member 13b	NM_001145645 /// NM_006573	8.7329	1.6453	0.1884	3.1281	0.0000
220658_s_at	ARNTL2	aryl hydrocarbon receptor nuclear translocator-like 2	NM_020183	9.5970	1.6364	0.1705	3.1088	0.0000
218700_s_at	RAB7L1	RAB7, member RAS oncogene family-like 1	NM_001135662 /// NM_001135663 /// NM_001135664 /// NM_003929	7.6841	1.6337	0.2126	3.1031	0.0000

Table S2. Continued

Probe Set ID	Gene Symbol	Gene Title	RefSeq Transcript ID	Score(d)	Numerat-or(r)	Denominator(s+s0)	Fold Change	q-value(%)
Up reglulated								
216594_x_at	AKR1C1	aldo-keto reductase family 1, member C1 (dihydrodiol dehydrogenase 1; 20-alpha (3-alpha)-hydroxysteroid dehydrogenase)	NM_001353	8.0917	1.6272	0.2011	3.0892	0.0000
225975_at	PCDH18	protocadherin 18	NM_019035	3.8125	1.6122	0.4229	3.0572	0.0000
218856_at	TNFRSF21	tumor necrosis factor receptor superfamily, member 21	NM_014452	6.8244	1.6118	0.2362	3.0564	0.0000
213110_s_at	COL4A5	collagen, type IV, alpha 5	NM_000495 /// NM_033380	7.3058	1.6115	0.2206	3.0557	0.0000
212481_s_at	TPM4	tropomyosin 4	NM_001145160 /// NM_003290	3.6953	1.6106	0.4359	3.0538	0.0000
205303_at	KCNJ8	potassium inwardly-rectifying channel, subfamily J, member 8	NM_004982	4.1036	1.6047	0.3910	3.0414	0.0000
204698_at	ISG20	interferon stimulated exonuclease gene 20kDa	NM_002201	10.4057	1.6038	0.1541	3.0393	0.0000
242625_at	RSAD2	radical S-adenosyl methionine domain containing 2	NM_080657	3.1565	1.6002	0.5070	3.0318	0.0852
1567107_s_at	TPM4	tropomyosin 4	NM_001145160 /// NM_003290	4.5918	1.5981	0.3480	3.0274	0.0000
235062_at	PIH1D2	PIH1 domain containing 2	NM_001082619 /// NM_138789	4.2790	1.5923	0.3721	3.0154	0.0000
202662_s_at	ITPR2	inositol 1,4,5-triphosphate receptor, type 2	NM_002223	7.2679	1.5888	0.2186	3.0080	0.0000

Table S2. Continued

Probe Set ID	Gene Symbol	Gene Title	RefSeq Transcript ID	Score(d)	Numerat-or(r)	Denominat or(s+s0)	Fold Change	q-value(%)
Up reglulated								
210145_at	PLA2G4A	phospholipase A2, group IVA (cytosolic, calcium-dependent)	NM_024420	3.4573	1.5849	0.4584	2.9999	0.0592
213022_s_at	UTRN	utrophin	NM_007124	8.4467	1.5816	0.1872	2.9931	0.0000
1555786_s_at	C14orf34	chromosome 14 open reading frame 34	NR_026796 /// NR_026797	8.2513	1.5808	0.1916	2.9913	0.0000
218699_at	RAB7L1	RAB7, member RAS oncogene family-like 1	NM_001135662 /// NM_001135663 /// NM_001135664 /// NM_003929	6.8330	1.5671	0.2293	2.9632	0.0000
209012_at	TRIO	triple functional domain (PTPRF interacting)	NM_007118	10.5265	1.5606	0.1483	2.9498	0.0000
224204_x_at	ARNTL2	aryl hydrocarbon receptor nuclear translocator-like 2	NM_020183	7.8367	1.5596	0.1990	2.9476	0.0000
207620_s_at	CASK	calcium/calmodulin-dependent serine protein kinase (MAGUK family)	NM_001126054 /// NM_001126055 /// NM_003688	5.0115	1.5572	0.3107	2.9428	0.0000
202457_s_at	PPP3CA	protein phosphatase 3, catalytic subunit, alpha isozyme	NM_000944 /// NM_001130691 /// NM_001130692	11.1788	1.5469	0.1384	2.9218	0.0000
241805_at	GABRG1	gamma-aminobutyric acid (GABA) A receptor, gamma 1	NM_173536	3.6294	1.5435	0.4253	2.9150	0.0592

Table S2. Continued

Probe Set ID	Gene Symbol	Gene Title	RefSeq Transcript ID	Score(d)	Numerat-or(r)	Denominat or(s+s0)	Fold Change	q-value(%)
Up reglulated								
213478_at	KAZ	kazrin	NM_001017999 /// NM_001018000 /// NM_001018001 /// NM_015209 /// NM_201628	8.0688	1.5434	0.1913	2.9148	0.0000
210519_s_at	NQO1	NAD(P)H dehydrogenase, quinone 1	NM_000903 /// NM_001025433 /// NM_001025434	6.5614	1.5385	0.2345	2.9050	0.0000
204540_at	EEF1A2	eukaryotic translation elongation factor 1 alpha 2	NM_001958	6.3082	1.5373	0.2437	2.9025	0.0000
209276_s_at	GLRX	glutaredoxin (thioltransferase)	NM_001118890 /// NM_002064	8.8666	1.5358	0.1732	2.8994	0.0000
213797_at	RSAD2	radical S-adenosyl methionine domain containing 2	NM_080657	6.7986	1.5350	0.2258	2.8979	0.0000
202157_s_at	CELF2	CUGBP, Elav-like family member 2	NM_001025076 /// NM_001025077 /// NM_001083591 /// NM_006561	7.3356	1.5273	0.2082	2.8824	0.0000
219655_at	C7orf10	chromosome 7 open reading frame 10	NM_001193311 /// NM_001193312 /// NM_001193313 /// NM_024728	7.6037	1.5259	0.2007	2.8797	0.0000
230438_at	TBX15	T-box 15	NM_152380	3.9198	1.5214	0.3881	2.8707	0.0000

Table S2. Continued

Probe Set ID	Gene Symbol	Gene Title	RefSeq Transcript ID	Score(d)	Numerat-or(r)	Denominat or(s+s0)	Fold Change	q-value(%)
Up reglulated								
226576_at	ARHGAP26	Rho GTPase activating protein 26	NM_001135608 /// NM_015071	6.0780	1.5206	0.2502	2.8691	0.0000
221185_s_at	IQCG	IQ motif containing G	NM_001134435 /// NM_032263	5.9837	1.5195	0.2539	2.8668	0.0000
208981_at	PECAM1	platelet/endothelial cell adhesion molecule	NM_000442	7.4730	1.5185	0.2032	2.8650	0.0000
213456_at	SOSTDC1	sclerostin domain containing 1	NM_015464	4.4221	1.5172	0.3431	2.8624	0.0000
230708_at	PRICKLE1	prickle homolog 1 (Drosophila)	NM_001144881 /// NM_001144882 /// NM_001144883 /// NM_153026	5.3991	1.5171	0.2810	2.8622	0.0000
212762_s_at	TCF7L2	transcription factor 7-like 2 (T-cell specific, HMG-box)	NM_001146274 /// NM_001146283 /// NM_001146284 /// NM_001146285 /// NM_001146286 /// NM_001198525 /// NM_001198526 /// NM_001198527 /// NM_001198528 /// NM_001198529 /// NM_001198530 /// NM_001198531 /// NM_030756	8.9865	1.5099	0.1680	2.8478	0.0000

Table S2. Continued

Probe Set ID	Gene Symbol	Gene Title	RefSeq Transcript ID	Score(d)	Numerat-or(r)	Denominat or(s+s0)	Fold Change	q-value(%)
Up reglulated								
209344_at	TPM4	tropomyosin 4	NM_001145160 /// NM_003290	4.4125	1.5063	0.3414	2.8408	0.0000
212510_at	GPD1L	glycerol-3-phosphate dehydrogenase 1-like	NM_015141	6.9024	1.5017	0.2176	2.8317	0.0000
212254_s_at	DST	dystonin	NM_001723 /// NM_015548	6.8051	1.4995	0.2203	2.8275	0.0000
203642_s_at	COBLL1	COBL-like 1	NM_014900	2.7060	1.4984	0.5537	2.8252	0.1046
205422_s_at	ITGBL1	integrin, beta-like 1 (with EGF-like repeat domains)	NM_004791	4.3425	1.4931	0.3438	2.8149	0.0000
229376_at	PROX1	prospero homeobox 1	NM_002763	8.4485	1.4892	0.1763	2.8073	0.0000
208869_s_at	GABARAPL 1	GABA(A) receptor-associated protein like 1	NM_031412	4.4330	1.4854	0.3351	2.8000	0.0000
226208_at	ZSWIM6	zinc finger, SWIM-type containing 6	NM_020928	6.9272	1.4807	0.2138	2.7909	0.0000
215016_x_at	DST	dystonin	NM_001723 /// NM_015548	7.7200	1.4774	0.1914	2.7844	0.0000
226084_at	MAP1B	microtubule-associated protein 1B	NM_005909	5.0645	1.4749	0.2912	2.7796	0.0000
214745_at	PLCH1	phospholipase C, eta 1	NM_001130960 /// NM_001130961 /// NM_014996	6.2754	1.4673	0.2338	2.7651	0.0000
1554445_at	ZNF85	zinc finger protein 85	NM_003429 /// NR_034060	3.5965	1.4631	0.4068	2.7571	0.0592

Table S2. Continued

Probe Set ID	Gene Symbol	Gene Title	RefSeq Transcript ID	Score(d)	Numerat-or(r)	Denominator(s+s0)	Fold Change	q-value(%)
Up reglulated								
229144_at	KAZ	kazrin	NM_001017999 /// NM_001018000 /// NM_001018001 /// NM_015209 /// NM_201628	4.7174	1.4574	0.3089	2.7462	0.0000
1569218_at	NA	NA	NA	4.4887	1.4568	0.3245	2.7449	0.0000
212761_at	TCF7L2	transcription factor 7-like 2 (T-cell specific, HMG-box)	NM_001146274 /// NM_001146283 /// NM_001146284 /// NM_001146285 /// NM_001146286 /// NM_001198525 /// NM_001198526 /// NM_001198527 /// NM_001198528 /// NM_001198529 /// NM_001198530 /// NM_001198531 /// NM_030756	7.5063	1.4556	0.1939	2.7427	0.0000
235094_at	NA	NA	NA	4.7285	1.4553	0.3078	2.7421	0.0000
227688_at	LRCH2	leucine-rich repeats and calponin homology (CH) domain containing 2	NM_020871	4.4312	1.4534	0.3280	2.7385	0.0000
235422_at	NA	NA	NA	5.5924	1.4494	0.2592	2.7310	0.0000

Table S2. Continued

Probe Set ID	Gene Symbol	Gene Title	RefSeq Transcript ID	Score(d)	Numerat-or(r)	Denominator(s+s0)	Fold Change	q-value(%)
Up reglulated								
223501_at	TNFSF13B	tumor necrosis factor (ligand) superfamily, member 13b	NM_001145645 /// NM_006573	8.0022	1.4469	0.1808	2.7262	0.0000
214765_s_at	NAAA	N-acylethanolamine acid amidase	NM_001042402 /// NM_014435	5.4613	1.4444	0.2645	2.7214	0.0000
228621_at	HFE2	hemochromatosis type 2 (juvenile)	NM_145277 /// NM_202004 /// NM_213652 /// NM_213653	6.2693	1.4440	0.2303	2.7207	0.0000
207712_at	BAGE	B melanoma antigen	NM_001187	5.9609	1.4422	0.2420	2.7174	0.0000
227080_at	ZNF697	zinc finger protein 697	NM_001080470	4.6601	1.4295	0.3067	2.6935	0.0000
201467_s_at	NQO1	NAD(P)H dehydrogenase, quinone 1	NM_000903 /// NM_001025433 /// NM_001025434	6.2382	1.4270	0.2287	2.6889	0.0000
208178_x_at	TRIO	triple functional domain (PTPRF interacting)	NM_007118	3.9588	1.4247	0.3599	2.6845	0.0000
213023_at	UTRN	utrophin	NM_007124	5.4597	1.4149	0.2592	2.6665	0.0000
214577_at	MAP1B	microtubule-associated protein 1B	NM_005909	3.9475	1.4122	0.3577	2.6614	0.0000
1556156_at	ESRRB	estrogen-related receptor beta	NM_004452	3.2288	1.4092	0.4364	2.6559	0.0852
223264_at	MESDC1	mesoderm development candidate 1	NM_022566	5.2006	1.4065	0.2705	2.6510	0.0000
206662_at	GLRX	glutaredoxin (thioltransferase)	NM_001118890 /// NM_002064	6.4884	1.4061	0.2167	2.6503	0.0000
213186_at	DZIP3	DAZ interacting protein 3, zinc finger	NM_014648	5.0706	1.4045	0.2770	2.6472	0.0000

Table S2. Continued

Probe Set ID	Gene Symbol	Gene Title	RefSeq Transcript ID	Score(d)	Numerat-or(r)	Denominat or(s+s0)	Fold Change	q-value(%)
Up reglulated								
207528_s_at	SLC7A11	solute carrier family 7, (cationic amino acid transporter, y+ system) member 11	NM_014331	2.6685	1.4027	0.5257	2.6440	0.1046
202664_at	WIPF1	WAS/WASL interacting protein family, member 1	NM_001077269 /// NM_003387	4.1048	1.3985	0.3407	2.6362	0.0000
205463_s_at	PDGFA	platelet-derived growth factor alpha polypeptide	NM_002607 /// NM_033023	4.2909	1.3943	0.3249	2.6285	0.0000
226905_at	FAM101B	family with sequence similarity 101, member B	NM_182705	4.3136	1.3847	0.3210	2.6113	0.0000
220273_at	IL17B	interleukin 17B	NM_014443	4.3679	1.3827	0.3166	2.6076	0.0000
209011_at	TRIO	triple functional domain (PTPRF interacting)	NM_007118	4.3892	1.3792	0.3142	2.6013	0.0000
207446_at	TLR6	toll-like receptor 6	NM_006068	5.0853	1.3724	0.2699	2.5891	0.0000
234111_at	NA	NA	NA	4.4260	1.3679	0.3091	2.5810	0.0000
206632_s_at	APOBEC3B	apolipoprotein B mRNA editing enzyme, catalytic polypeptide-like 3B	NM_004900	6.6687	1.3668	0.2050	2.5790	0.0000
204099_at	SMARCD3	SWI/SNF related, matrix associated, actin dependent regulator of chromatin, subfamily d, member 3	NM_001003801 /// NM_001003802 /// NM_003078	6.4904	1.3629	0.2100	2.5720	0.0000
202686_s_at	AXL	AXL receptor tyrosine kinase	NM_001699 /// NM_021913	2.8653	1.3617	0.4752	2.5699	0.1046
213182_x_at	CDKN1C	cyclin-dependent kinase inhibitor 1C (p57, Kip2)	NM_000076 /// NM_001122630 /// NM_001122631	3.0782	1.3617	0.4424	2.5699	0.0852

Table S2. Continued

Probe Set ID	Gene Symbol	Gene Title	RefSeq Transcript ID	Score(d)	Numerat-or(r)	Denominator(s+s0)	Fold Change	q-value(%)
Up reglulated								
213959_s_at	RPGRIP1L	RPGRIP1-like	NM_001127897 /// NM_015272	5.0951	1.3602	0.2670	2.5673	0.0000
202295_s_at	CTSH	cathepsin H	NM_004390	4.5156	1.3563	0.3004	2.5602	0.0000
228771_at	ADRBK2	adrenergic, beta, receptor kinase 2	NM_005160	4.8770	1.3528	0.2774	2.5540	0.0000
1552643_at	ZNF626	zinc finger protein 626	NM_001076675 /// NM_145297	5.0411	1.3526	0.2683	2.5536	0.0000
1553646_at	HDX	highly divergent homeobox	NM_001177478 /// NM_001177479 /// NM_144657	7.7051	1.3518	0.1754	2.5523	0.0000
217562_at	FAM5C	family with sequence similarity 5, member C	NM_199051	2.9817	1.3500	0.4528	2.5492	0.1046
232127_at	CLCN5	chloride channel 5	NM_000084 /// NM_001127898 /// NM_001127899	2.8384	1.3456	0.4741	2.5414	0.1046
221773_at	ELK3	ELK3, ETS-domain protein (SRF accessory protein 2)	NM_005230	5.2153	1.3429	0.2575	2.5365	0.0000
236154_at	QKI	quaking homolog, KH domain RNA binding (mouse)	NM_006775 /// NM_206853 /// NM_206854 /// NM_206855	5.3581	1.3390	0.2499	2.5297	0.0000
243358_at	IGF1R	insulin-like growth factor 1 receptor	NM_000875	5.7914	1.3374	0.2309	2.5270	0.0000
202388_at	RGS2	regulator of G-protein signaling 2, 24kDa	NM_002923	5.4815	1.3355	0.2436	2.5236	0.0000

Table S2. Continued

Probe Set ID	Gene Symbol	Gene Title	RefSeq Transcript ID	Score(d)	Numerat-or(r)	Denominator(s+s0)	Fold Change	q-value(%)
Up reglulated								
218309_at	CAMK2N1	calcium/calmodulin-dependent protein kinase II inhibitor 1	NM_018584	5.2702	1.3337	0.2531	2.5204	0.0000
1553199_at	DCAF4L2	DDB1 and CUL4 associated factor 4-like 2	NM_152418	6.5925	1.3327	0.2022	2.5188	0.0000
232267_at	GPR133	G protein-coupled receptor 133	NM_198827	7.7157	1.3314	0.1726	2.5164	0.0000
204500_s_at	AGTPBP1	ATP/GTP binding protein 1	NM_015239	3.1382	1.3301	0.4239	2.5142	0.0852
213348_at	CDKN1C	cyclin-dependent kinase inhibitor 1C (p57, Kip2)	NM_000076 /// NM_001122630 /// NM_001122631	3.6346	1.3206	0.3633	2.4977	0.0592
208949_s_at	LGALS3	lectin, galactoside-binding, soluble, 3	NM_001177388 /// NM_002306 /// NR_003225	4.6258	1.3195	0.2853	2.4959	0.0000
1553976_a_at	DPCD	deleted in primary ciliary dyskinesia homolog (mouse)	NM_015448	8.0517	1.3182	0.1637	2.4935	0.0000
219654_at	PTPLA	protein tyrosine phosphatase-like (proline instead of catalytic arginine), member A	NM_014241	3.9837	1.3180	0.3308	2.4932	0.0000
213836_s_at	WIPI1	WD repeat domain, phosphoinositide interacting 1	NM_017983	3.4889	1.3168	0.3774	2.4911	0.0592
234623_x_at	NA	NA	NA	2.5998	1.3168	0.5065	2.4911	0.1519
242447_at	C3orf70	chromosome 3 open reading frame 70	NM_001025266	7.8756	1.3127	0.1667	2.4840	0.0000
205011_at	VWA5A	von Willebrand factor A domain containing 5A	NM_001130142 /// NM_014622 /// NM_198315	4.9137	1.3124	0.2671	2.4835	0.0000

Table S2. Continued

Probe Set ID	Gene Symbol	Gene Title	RefSeq Transcript ID	Score(d)	Numerat-or(r)	Denominat or(s+s0)	Fold Change	q-value(%)
Up reglulated								
211071_s_at	MLLT11	myeloid/lymphoid or mixed-lineage leukemia (trithorax homolog, Drosophila); translocated to, 11	NM_006818	4.6732	1.3103	0.2804	2.4799	0.0000
211458_s_at	GABARAPL1 /// GABARAPL3	GABA(A) receptor-associated protein like 1 /// GABA(A) receptors associated protein like 3, pseudogene	NM_031412 /// NR_028287	4.7657	1.3091	0.2747	2.4779	0.0000
209295_at	TNFRSF10B	tumor necrosis factor receptor superfamily, member 10b	NM_003842 /// NM_147187 /// NR_027140	6.4270	1.3041	0.2029	2.4694	0.0000
219576_at	MAP7D3	MAP7 domain containing 3	NM_001173516 /// NM_001173517 /// NM_024597	5.3540	1.2998	0.2428	2.4620	0.0000
225524_at	ANTXR2	anthrax toxin receptor 2	NM_001145794 /// NM_058172	4.0585	1.2971	0.3196	2.4573	0.0000
209883_at	GLT25D2	glycosyltransferase 25 domain containing 2	NM_015101	2.6653	1.2950	0.4859	2.4537	0.1046
203473_at	SLCO2B1	solute carrier organic anion transporter family, member 2B1	NM_001145211 /// NM_001145212 /// NM_007256	4.3189	1.2880	0.2982	2.4419	0.0000
1553197_at	DCAF4L2	DDB1 and CUL4 associated factor 4-like 2	NM_152418	6.2058	1.2827	0.2067	2.4329	0.0000
205194_at	PSPH	phosphoserine phosphatase	NM_004577	4.6055	1.2823	0.2784	2.4322	0.0000

Table S2. Continued

Probe Set ID	Gene Symbol	Gene Title	RefSeq Transcript ID	Score(d)	Numerat-or(r)	Denominat or(s+s0)	Fold Change	q-value(%)
Up reglulated								
228573_at	ANTXR2	anthrax toxin receptor 2	NM_001145794 /// NM_058172	4.4690	1.2819	0.2869	2.4316	0.0000
224391_s_at	SIAE	sialic acid acetylesterase	NM_170601	6.1638	1.2785	0.2074	2.4258	0.0000
201341_at	ENC1	ectodermal-neural cortex 1 (with BTB-like domain)	NM_003633	3.8613	1.2757	0.3304	2.4212	0.0000
235498_at	LRRIQ3	leucine-rich repeats and IQ motif containing 3	NM_001105659	3.9293	1.2725	0.3238	2.4158	0.0000
223741_s_at	TTYH2	tweety homolog 2 (Drosophila)	NM_032646 /// NM_052869	4.8594	1.2663	0.2606	2.4054	0.0000
201005_at	CD9	CD9 molecule	NM_001769	3.8325	1.2628	0.3295	2.3996	0.0000
209135_at	ASPH	aspartate beta-hydroxylase	NM_001164750 /// NM_001164751 /// NM_001164752 /// NM_001164753 /// NM_001164754 /// NM_001164755 /// NM_001164756 /// NM_004318 /// NM_020164 /// NM_032466 /// NM_032467 /// NM_032468	6.3271	1.2626	0.1996	2.3993	0.0000

Table S2. Continued

Probe Set ID	Gene Symbol	Gene Title	RefSeq Transcript ID	Score(d)	Numerat-or(r)	Denominator(s+s0)	Fold Change	q-value(%)
Up reglulated								
219737_s_at	PCDH9	protocadherin 9	NM_020403 /// NM_203487	5.1691	1.2612	0.2440	2.3970	0.0000
203438_at	STC2	stanniocalcin 2	NM_003714	3.1392	1.2585	0.4009	2.3924	0.0852
228724_at	TTLL7	tubulin tyrosine ligase-like family, member 7	NM_024686	4.3664	1.2554	0.2875	2.3874	0.0000
232128_s_at	CLCN5	chloride channel 5	NM_000084 /// NM_001127898 /// NM_001127899	2.6915	1.2497	0.4643	2.3779	0.1046
214240_at	GAL	galanin prepropeptide	NM_015973	5.4373	1.2477	0.2295	2.3746	0.0000
224797_at	ARRDC3	arrestin domain containing 3	NM_020801	3.6948	1.2451	0.3370	2.3703	0.0000
219132_at	PELI2	pellino homolog 2 (Drosophila)	NM_021255	2.8777	1.2408	0.4312	2.3634	0.1046
207180_s_at	HTATIP2	HIV-1 Tat interactive protein 2, 30kDa	NM_001098520 /// NM_001098521 /// NM_001098522 /// NM_001098523 /// NM_006410	5.2504	1.2403	0.2362	2.3624	0.0000
203641_s_at	COBLL1	COBL-like 1	NM_014900	4.5572	1.2367	0.2714	2.3565	0.0000
200635_s_at	PTPRF	protein tyrosine phosphatase, receptor type, F	NM_002840 /// NM_130440	5.0713	1.2345	0.2434	2.3529	0.0000
213624_at	SMPDL3A	sphingomyelin phosphodiesterase, acid-like 3A	NM_006714	6.2556	1.2300	0.1966	2.3457	0.0000

Table S2. Continued

Probe Set ID	Gene Symbol	Gene Title	RefSeq Transcript ID	Score(d)	Numerat-or(r)	Denominat or(s+s0)	Fold Change	q-value(%)
Up reglulated								
206704_at	CLCN5	chloride channel 5	NM_000084 /// NM_001127898 /// NM_001127899	2.5665	1.2217	0.4760	2.3323	0.1519
209298_s_at	ITSN1	intersectin 1 (SH3 domain protein)	NM_001001132 /// NM_003024	3.6647	1.2199	0.3329	2.3294	0.0000
203427_at	ASF1A	ASF1 anti-silencing function 1 homolog A (S. cerevisiae)	NM_014034	5.7649	1.2187	0.2114	2.3273	0.0000
227176_at	SLC2A13	solute carrier family 2 (facilitated glucose transporter), member 13	NM_052885	6.0155	1.2130	0.2016	2.3182	0.0000
203914_x_at	HPGD	hydroxyprostaglandin dehydrogenase 15-(NAD)	NM_000860 /// NM_001145816 /// NR_027332	4.7177	1.2107	0.2566	2.3145	0.0000
219534_x_at	CDKN1C	cyclin-dependent kinase inhibitor 1C (p57, Kip2)	NM_000076 /// NM_001122630 /// NM_001122631	3.0457	1.2081	0.3967	2.3103	0.0852
203913_s_at	HPGD	hydroxyprostaglandin dehydrogenase 15-(NAD)	NM_000860 /// NM_001145816 /// NR_027332	4.6828	1.2042	0.2571	2.3040	0.0000
234432_at	NA	NA	NA	2.8754	1.2039	0.4187	2.3036	0.1046
216620_s_at	ARHGEF10	Rho guanine nucleotide exchange factor (GEF) 10	NM_014629	5.3031	1.2006	0.2264	2.2984	0.0000
221606_s_at	HMGN5	high-mobility group nucleosome binding domain 5	NM_030763	2.9304	1.1979	0.4088	2.2941	0.1046

Table S2. Continued

Probe Set ID	Gene Symbol	Gene Title	RefSeq Transcript ID	Score(d)	Numerat-or(r)	Denominator(s+s0)	Fold Change	q-value(%)
Up reglulated								
219506_at	C1orf54	chromosome 1 open reading frame 54	NM_024579	4.5707	1.1901	0.2604	2.2816	0.0000
227963_at	NA	NA	NA	4.0319	1.1898	0.2951	2.2812	0.0000
213392_at	IQCK	IQ motif containing K	NM_153208	4.4160	1.1891	0.2693	2.2802	0.0000
218829_s_at	CHD7	chromodomain helicase DNA binding protein 7	NM_017780	6.8199	1.1885	0.1743	2.2792	0.0000
201858_s_at	SRGN	serglycin	NM_002727 /// NR_036430	4.0511	1.1882	0.2933	2.2786	0.0000
231018_at	PALM3	paralemmin 3	NM_001145028	4.1820	1.1871	0.2839	2.2770	0.0000
209732_at	CLEC2B	C-type lectin domain family 2, membe B	NM_005127	3.5972	1.1870	0.3300	2.2768	0.0592
210113_s_at	NLRP1	NLR family, pyrin domain containing 1	NM_001033053 /// NM_014922 /// NM_033004 /// NM_033006 /// NM_033007	8.6279	1.1861	0.1375	2.2753	0.0000
244667_at	NA	NA	NA	5.8595	1.1838	0.2020	2.2718	0.0000
208983_s_at	PECAM1	platelet/endothelial cell adhesion molecule	NM_000442	3.7279	1.1818	0.3170	2.2685	0.0000
202108_at	PEPD	peptidase D	NM_000285 /// NM_001166056 /// NM_001166057	6.5715	1.1803	0.1796	2.2663	0.0000
239768_x_at	GPATCH2	G patch domain containing 2	NM_018040	5.1797	1.1793	0.2277	2.2646	0.0000
228152_s_at	DDX60L	DEAD (Asp-Glu-Ala-Asp) box polypeptide 60-like	NM_001012967	5.8163	1.1775	0.2024	2.2619	0.0000

Table S2. Continued

Probe Set ID	Gene Symbol	Gene Title	RefSeq Transcript ID	Score(d)	Numerat-or(r)	Denominat or(s+s0)	Fold Change	q-value(%)
Up reglulated								
226009_at	DPCD	deleted in primary ciliary dyskinesia homolog (mouse)	NM_015448	6.9523	1.1771	0.1693	2.2612	0.0000
219557_s_at	NRIP3	nuclear receptor interacting protein 3	NM_020645	3.0441	1.1765	0.3865	2.2603	0.0852
225265_at	RBMS1	RNA binding motif, single stranded interacting protein 1	NM_002897 /// NM_016836	3.5936	1.1743	0.3268	2.2569	0.0592
202668_at	EFNB2	ephrin-B2	NM_004093	5.4299	1.1725	0.2159	2.2540	0.0000
211824_x_at	NLRP1	NLR family, pyrin domain containing 1	NM_001033053 /// NM_014922 /// NM_033004 /// NM_033006 /// NM_033007	4.4029	1.1697	0.2657	2.2497	0.0000
210176_at	TLR1	toll-like receptor 1	NM_003263	4.6240	1.1693	0.2529	2.2490	0.0000
219626_at	MAP7D3	MAP7 domain containing 3	NM_001173516 /// NM_001173517 /// NM_024597	7.3253	1.1662	0.1592	2.2442	0.0000
219522_at	FJX1	four jointed box 1 (Drosophila)	NM_014344	5.5979	1.1640	0.2079	2.2408	0.0000
217269_s_at	TMPRSS15	transmembrane protease, serine 15	NM_002772	5.5323	1.1620	0.2100	2.2377	0.0000
203827_at	WIPI1	WD repeat domain, phosphoinositide interacting 1	NM_017983	2.7714	1.1588	0.4181	2.2328	0.1046
1559867_at	NA	NA	NA	5.2473	1.1588	0.2208	2.2327	0.0000
238050_at	ANTXR2	anthrax toxin receptor 2	NM_001145794 /// NM_058172	3.9710	1.1582	0.2917	2.2317	0.0000
219078_at	GPATCH2	G patch domain containing 2	NM_018040	6.5585	1.1573	0.1765	2.2304	0.0000

Table S2. Continued

Probe Set ID	Gene Symbol	Gene Title	RefSeq Transcript ID	Score(d)	Numerat-or(r)	Denominat or(s+s0)	Fold Change	q-value(%)
Up reglulated								
208906_at	BSCL2	Berardinelli-Seip congenital lipodystrophy 2 (seipin)	NM_001122955 /// NM_001130702 /// NM_032667	5.2469	1.1564	0.2204	2.2290	0.0000
1558882_at	LOC401233	HIV-1 Tat specific factor 1 pseudogene	NR_033884	3.0447	1.1556	0.3796	2.2278	0.0852
210405_x_at	TNFRSF10B	tumor necrosis factor receptor superfamily, member 10b	NM_003842 /// NM_147187 /// NR_027140	4.4520	1.1548	0.2594	2.2265	0.0000
1556134_a_at	B3GNT5	UDP-GlcNAc:betaGal beta-1,3-N-acetylglucosaminyltransferase 5	NM_032047	3.8645	1.1507	0.2978	2.2202	0.0000
207038_at	SLC16A6	solute carrier family 16, member 6 (monocarboxylic acid transporter 7)	NM_001174166 /// NM_004694	4.0212	1.1503	0.2861	2.2196	0.0000
33304_at	ISG20	interferon stimulated exonuclease gene 20kDa	NM_002201	6.4069	1.1493	0.1794	2.2181	0.0000
223169_s_at	RHOU	ras homolog gene family, member U	NM_021205	5.1057	1.1427	0.2238	2.2079	0.0000
227817_at	PRKCB	protein kinase C, beta	NM_002738 /// NM_212535	4.9930	1.1418	0.2287	2.2065	0.0000
227824_at	PRKCB	protein kinase C, beta	NM_002738 /// NM_212535	4.4077	1.1386	0.2583	2.2017	0.0000
207401_at	PROX1	prospero homeobox 1	NM_002763	3.1630	1.1352	0.3589	2.1965	0.0852
226273_at	CLCN5	chloride channel 5	NM_000084 /// NM_001127898 /// NM_001127899	3.3617	1.1347	0.3375	2.1957	0.0592

Table S2. Continued

Probe Set ID	Gene Symbol	Gene Title	RefSeq Transcript ID	Score(d)	Numerat-or(r)	Denominator(s+s0)	Fold Change	q-value(%)
Up reglulated								
216894_x_at	CDKN1C	cyclin-dependent kinase inhibitor 1C (p57, Kip2)	NM_000076 /// NM_001122630 /// NM_001122631	3.0649	1.1346	0.3702	2.1955	0.0852
222494_at	FOXN3	forkhead box N3	NM_001085471 /// NM_005197	2.6235	1.1345	0.4324	2.1954	0.1519
226123_at	CHD7	chromodomain helicase DNA binding protein 7	NM_017780	5.4693	1.1316	0.2069	2.1911	0.0000
209283_at	CRYAB	crystallin, alpha B	NM_001885	3.5772	1.1298	0.3158	2.1883	0.0592
219489_s_at	NXN	nucleoredoxin	NM_022463	2.6427	1.1292	0.4273	2.1874	0.1519
218380_at	LOC728392 /// NLRP1	hypothetical protein LOC728392 /// NLR family, pyrin domain containing 1	NM_001033053 /// NM_001162371 /// NM_014922 /// NM_033004 /// NM_033006 /// NM_033007	5.2913	1.1247	0.2126	2.1806	0.0000
208982_at	PECAM1	platelet/endothelial cell adhesion molecule	NM_000442	4.7000	1.1246	0.2393	2.1803	0.0000
214430_at	GLA	galactosidase, alpha	NM_000169	4.6965	1.1227	0.2390	2.1775	0.0000
235143_at	SLC10A7	solute carrier family 10 (sodium/bile acid cotransporter family), member 7	NM_001029998 /// NM_032128	8.4716	1.1212	0.1323	2.1752	0.0000
204059_s_at	ME1	malic enzyme 1, NADP(+)-dependent, cytosolic	NM_002395	5.1774	1.1159	0.2155	2.1673	0.0000
221156_x_at	CCPG1	cell cycle progression 1	NM_004748 /// NM_020739	3.8890	1.1151	0.2867	2.1662	0.0000

Table S2. Continued

Probe Set ID	Gene Symbol	Gene Title	RefSeq Transcript ID	Score(d)	Numerat-or(r)	Denominator(s+s0)	Fold Change	q-value(%)
Up reglulated								
1559393_at	ALDH1L2	aldehyde dehydrogenase 1 family, member L2	NM_001034173 /// NR_027752	4.0929	1.1139	0.2722	2.1643	0.0000
233011_at	ANXA1	Annexin A1	NM_000700	3.2993	1.1112	0.3368	2.1603	0.0592
200637_s_at	PTPRF	protein tyrosine phosphatase, receptor type, F	NM_002840 /// NM_130440	3.4497	1.1109	0.3220	2.1599	0.0592
209448_at	HTATIP2	HIV-1 Tat interactive protein 2, 30kDa	NM_001098520 /// NM_001098521 /// NM_001098522 /// NM_001098523 /// NM_006410	6.0396	1.1100	0.1838	2.1585	0.0000
203946_s_at	ARG2	arginase, type II	NM_001172	3.1010	1.1084	0.3574	2.1560	0.0852
200730_s_at	PTP4A1	protein tyrosine phosphatase type IVA, member 1	NM_003463	2.8365	1.1065	0.3901	2.1532	0.1046
209013_x_at	TRIO	triple functional domain (PTPRF interacting)	NM_007118	9.6683	1.1059	0.1144	2.1524	0.0000
219155_at	PITPNC1	phosphatidylinositol transfer protein, cytoplasmic 1	NM_012417 /// NM_181671	6.4533	1.1023	0.1708	2.1470	0.0000
201340_s_at	ENC1	ectodermal-neural cortex 1 (with BTB-like domain)	NM_003633	3.0177	1.1002	0.3646	2.1439	0.1046
206005_s_at	KIAA1009	KIAA1009	NM_014895	3.7712	1.0987	0.2913	2.1417	0.0000
203650_at	PROCR	protein C receptor, endothelial	NM_006404	3.2840	1.0969	0.3340	2.1389	0.0592
200632_s_at	NDRG1	N-myc downstream regulated 1	NM_001135242 /// NM_006096	4.6057	1.0960	0.2380	2.1376	0.0000

Table S2. Continued

Probe Set ID	Gene Symbol	Gene Title	RefSeq Transcript ID	Score(d)	Numerat-or(r)	Denominator(s+s0)	Fold Change	q-value(%)
Up reglulated								
224036_s_at	LMBR1	limb region 1 homolog (mouse)	NM_022458	2.9462	1.0929	0.3710	2.1330	0.1046
1552695_a_at	SLC2A13	solute carrier family 2 (facilitated glucose transporter), member 13	NM_052885	3.7892	1.0913	0.2880	2.1307	0.0000
216833_x_at	GYPB	glycophorin B (MNS blood group)	NM_002100	3.5272	1.0895	0.3089	2.1280	0.0592
219403_s_at	HPSE	heparanase	NM_001098540 /// NM_001166498 /// NM_006665	2.9394	1.0894	0.3706	2.1279	0.1046
212063_at	CD44	CD44 molecule (Indian blood group)	NM_000610 /// NM_001001389 /// NM_001001390 /// NM_001001391 /// NM_001001392	2.6938	1.0885	0.4041	2.1265	0.1046
211822_s_at	NLRP1	NLR family, pyrin domain containing 1	NM_001033053 /// NM_014922 /// NM_033004 /// NM_033006 /// NM_033007	4.8689	1.0869	0.2232	2.1242	0.0000
228795_at	PRKCB	protein kinase C, beta	NM_002738 /// NM_212535	4.2316	1.0849	0.2564	2.1212	0.0000
212636_at	QKI	quaking homolog, KH domain RNA binding (mouse)	NM_006775 /// NM_206853 /// NM_206854 /// NM_206855	6.9604	1.0823	0.1555	2.1174	0.0000

Table S2. Continued

Probe Set ID	Gene Symbol	Gene Title	RefSeq Transcript ID	Score(d)	Numerat-or(r)	Denominat or(s+s0)	Fold Change	q-value(%)
Up reglulated								
210553_x_at	PCSK6	proprotein convertase subtilisin/kexin type 6	NM_002570 /// NM_138319 /// NM_138320 /// NM_138321 /// NM_138322 /// NM_138323 /// NM_138324 /// NM_138325	5.0046	1.0809	0.2160	2.1153	0.0000
219963_at	DUSP13	dual specificity phosphatase 13	NM_001007271 /// NM_001007272 /// NM_001007273 /// NM_016364	6.2203	1.0793	0.1735	2.1130	0.0000
222900_at	NRIP3	nuclear receptor interacting protein 3	NM_020645	5.4979	1.0770	0.1959	2.1096	0.0000
230336_at	NA	NA	NA	4.2483	1.0724	0.2524	2.1030	0.0000

Table S2. Continued

Probe Set ID	Gene Symbol	Gene Title	RefSeq Transcript ID	Score(d)	Numerat-or(r)	Denominat or(s+s0)	Fold Change	q-value(%)
Up reglulated								
216035_x_at	TCF7L2	transcription factor 7-like 2 (T-cell specific, HMG-box)	NM_001146274 /// NM_001146283 /// NM_001146284 /// NM_001146285 /// NM_001146286 /// NM_001198525 /// NM_001198526 /// NM_001198527 /// NM_001198528 /// NM_001198529 /// NM_001198530 /// NM_001198531 /// NM_030756	4.5084	1.0721	0.2378	2.1025	0.0000
221946_at	C9orf116	chromosome 9 open reading frame 116	NM_001048265 /// NM_144654	4.0787	1.0699	0.2623	2.0993	0.0000
207459_x_at	GYPB	glycophorin B (MNS blood group)	NM_002100	3.4491	1.0686	0.3098	2.0974	0.0592
228539_at	NA	NA	NA	4.3731	1.0682	0.2443	2.0968	0.0000
229544_at	NA	NA	NA	4.2764	1.0671	0.2495	2.0952	0.0000
224168_at	TXNDC2	thioredoxin domain containing 2 (spermatozoa)	NM_001098529 /// NM_032243	4.6358	1.0643	0.2296	2.0911	0.0000

Table S2. Continued

Probe Set ID	Gene Symbol	Gene Title	RefSeq Transcript ID	Score(d)	Numerat-or(r)	Denominator(s+s0)	Fold Change	q-value(%)
Up reglulated								
223694_at	TRIM7	tripartite motif-containing 7	NM_033342 /// NM_203293 /// NM_203294 /// NM_203295 /// NM_203296 /// NM_203297	5.0856	1.0621	0.2088	2.0879	0.0000
221556_at	CDC14B	CDC14 cell division cycle 14 homolog B (S. cerevisiae)	NM_001077181 /// NM_003671 /// NM_033331	6.9101	1.0600	0.1534	2.0850	0.0000
215388_s_at	CFH /// CFHR1	complement factor H /// complement factor H-related 1	NM_000186 /// NM_001014975 /// NM_002113	7.0279	1.0566	0.1503	2.0800	0.0000
205356_at	USP13	ubiquitin specific peptidase 13 (isopeptidase T-3)	NM_003940	6.3254	1.0522	0.1663	2.0737	0.0000
223586_at	ARNTL2	aryl hydrocarbon receptor nuclear translocator-like 2	NM_020183	6.0467	1.0520	0.1740	2.0734	0.0000
226274_at	CLCN5	chloride channel 5	NM_000084 /// NM_001127898 /// NM_001127899	6.3687	1.0518	0.1652	2.0732	0.0000
205122_at	TMEFF1	transmembrane protein with EGF-like and two follistatin-like domains 1	NM_003692	2.5674	1.0517	0.4096	2.0729	0.1519
209288_s_at	CDC42EP3	CDC42 effector protein (Rho GTPase binding) 3	NM_006449	4.5057	1.0476	0.2325	2.0671	0.0000

Table S2. Continued

Probe Set ID	Gene Symbol	Gene Title	RefSeq Transcript ID	Score(d)	Numerat-or(r)	Denominator(s+s0)	Fold Change	q-value(%)
Up reglulated								
1554569_a_at	CELF2	CUGBP, Elav-like family member 2	NM_001025076 /// NM_001025077 /// NM_001083591 /// NM_006561	3.8535	1.0473	0.2718	2.0667	0.0000
232586_x_at	OR7E126P	olfactory receptor, family 7, subfamily E, member 126 pseudogene	NA	4.7400	1.0468	0.2208	2.0660	0.0000
206538_at	MRAS	muscle RAS oncogene homolog	NM_001085049 /// NM_012219	4.5541	1.0467	0.2298	2.0657	0.0000
200872_at	S100A10	S100 calcium binding protein A10	NM_002966	4.8198	1.0442	0.2166	2.0622	0.0000
228372_at	C10orf128	chromosome 10 open reading frame 128	NM_001010863	3.1408	1.0432	0.3321	2.0608	0.0852
216511_s_at	TCF7L2	transcription factor 7-like 2 (T-cell specific, HMG-box)	NM_001146274 /// NM_001146283 /// NM_001146284 /// NM_001146285 /// NM_001146286 /// NM_001198525 /// NM_001198526 /// NM_001198527 /// NM_001198528 /// NM_001198529 /// NM_001198530 /// NM_001198531 /// NM_030756	3.5166	1.0413	0.2961	2.0580	0.0592

Table S2. Continued

Probe Set ID	Gene Symbol	Gene Title	RefSeq Transcript ID	Score(d)	Numerat-or(r)	Denominat or(s+s0)	Fold Change	q-value(%)
Up reglulated								
239001_at	MGST1	Microsomal glutathione S-transferase 1	NM_020300 /// NM_145764 /// NM_145791 /// NM_145792	4.3037	1.0407	0.2418	2.0572	0.0000
243001_at	RBFA	ribosome binding factor A (putative)	NM_001171967 /// NM_024805	3.1393	1.0370	0.3303	2.0520	0.0852
236026_at	GPATCH2	G patch domain containing 2	NM_018040	4.5945	1.0368	0.2257	2.0517	0.0000
210138_at	RGS20	regulator of G-protein signaling 20	NM_003702 /// NM_170587	4.2570	1.0342	0.2429	2.0480	0.0000
210896_s_at	ASPH	aspartate beta-hydroxylase	NM_001164750 /// NM_001164751 /// NM_001164752 /// NM_001164753 /// NM_001164754 /// NM_001164755 /// NM_001164756 /// NM_004318 /// NM_020164 /// NM_032466 /// NM_032467 /// NM_032468	3.5308	1.0323	0.2924	2.0452	0.0592
207854_at	GYPE	glycophorin E (MNS blood group)	NM_002102 /// NM_198682	3.2712	1.0318	0.3154	2.0446	0.0592

Table S2. Continued

Probe Set ID	Gene Symbol	Gene Title	RefSeq Transcript ID	Score(d)	Numerat-or(r)	Denominator(s+s0)	Fold Change	q-value(%)
Up reglulated								
215127_s_at	RBMS1	RNA binding motif, single stranded interacting protein 1	NM_002897 /// NM_016836	5.1762	1.0301	0.1990	2.0422	0.0000
207957_s_at	PRKCB	protein kinase C, beta	NM_002738 /// NM_212535	6.7787	1.0288	0.1518	2.0403	0.0000
230766_at	GART	phosphoribosylglycinamide formyltransferase, phosphoribosylglycinamide synthetase, phosphoribosylaminoimidazole synthetase	NM_000819 /// NM_001136005 /// NM_001136006 /// NM_175085	8.9083	1.0279	0.1154	2.0391	0.0000
1555536_at	ANTXR2	anthrax toxin receptor 2	NM_001145794 /// NM_058172	2.6429	1.0246	0.3877	2.0344	0.1519
214830_at	SLC38A6	solute carrier family 38, member 6	NM_001172702 /// NM_153811 /// NR_033344	4.8202	1.0236	0.2123	2.0330	0.0000
230009_at	FAM118B	family with sequence similarity 118, member B	NM_024556	2.9839	1.0232	0.3429	2.0324	0.1046
212233_at	MAP1B	microtubule-associated protein 1B	NM_005909	3.8672	1.0225	0.2644	2.0314	0.0000
241853_at	NA	NA	NA	3.2662	1.0205	0.3124	2.0286	0.0592

Table S2. Continued

Probe Set ID	Gene Symbol	Gene Title	RefSeq Transcript ID	Score(d)	Numerat-or(r)	Denominat or(s+s0)	Fold Change	q-value(%)
Up reglulated								
216037_x_at	TCF7L2	transcription factor 7-like 2 (T-cell specific, HMG-box)	NM_001146274 /// NM_001146283 /// NM_001146284 /// NM_001146285 /// NM_001146286 /// NM_001198525 /// NM_001198526 /// NM_001198527 /// NM_001198528 /// NM_001198529 /// NM_001198530 /// NM_001198531 /// NM_030756	3.9278	1.0164	0.2588	2.0228	0.0000
1554465_s_at	ZNF673 /// ZNF674	zinc finger family member 673 /// zinc finger protein 674	NM_001039891 /// NM_001129898 /// NM_001129899 /// NM_001129900 /// NM_001146291 /// NM_001190417 /// NM_017776	3.6676	1.0082	0.2749	2.0114	0.0000
1556129_at	LOC642533	hypothetical LOC642533	XR_016325 /// XR_041225 /// XR_041226	4.7333	1.0074	0.2128	2.0103	0.0000

Table S2. Continued

Probe Set ID	Gene Symbol	Gene Title	RefSeq Transcript ID	Score(d)	Numerat-or(r)	Denominator(s+s0)	Fold Change	q-value(%)
Up reglulated								
220336_s_at	GP6	glycoprotein VI (platelet)	NM_001083899 /// NM_016363	2.6958	1.0037	0.3723	2.0051	0.1046
219477_s_at	THSD1 /// THSD1P1	thrombospondin, type I, domain containing 1 /// thrombospondin, type I, domain containing 1 pseudogene 1	NM_018676 /// NM_199263 /// NR_002816	5.2659	1.0031	0.1905	2.0043	0.0000
230968_at	NA	NA	NA	3.0763	0.9981	0.3245	1.9974	0.0852
209294_x_at	TNFRSF10B	tumor necrosis factor receptor superfamily, member 10b	NM_003842 /// NM_147187 /// NR_027140	3.0829	0.9962	0.3231	1.9948	0.0852
214407_x_at	GYPB	glycophorin B (MNS blood group)	NM_002100	3.4551	0.9944	0.2878	1.9922	0.0592
229285_at	RNASEL	ribonuclease L (2',5'-oligoisoadenylate synthetase-dependent)	NM_021133	4.6739	0.9920	0.2122	1.9890	0.0000
227766_at	LIG4	ligase IV, DNA, ATP-dependent	NM_001098268 /// NM_002312 /// NM_206937	4.7863	0.9915	0.2072	1.9883	0.0000
242228_at	NA	NA	NA	3.5090	0.9900	0.2821	1.9862	0.0592
229869_at	NA	NA	NA	5.5412	0.9886	0.1784	1.9842	0.0000
233527_at	LOC100129069	Hypothetical protein LOC100129069	XM_001717421 /// XM_001726472 /// XM_001726502	4.6236	0.9885	0.2138	1.9841	0.0000
206170_at	ADRB2	adrenergic, beta-2-, receptor, surface	NM_000024	4.5445	0.9880	0.2174	1.9834	0.0000
221669_s_at	ACAD8	acyl-CoA dehydrogenase family, member 8	NM_014384	4.6810	0.9863	0.2107	1.9811	0.0000

Table S2. Continued

Probe Set ID	Gene Symbol	Gene Title	RefSeq Transcript ID	Score(d)	Numerat-or(r)	Denominat or(s+s0)	Fold Change	q-value(%)
Up reglulated								
206254_at	EGF	epidermal growth factor	NM_001178130 /// NM_001178131 /// NM_001963	4.4851	0.9856	0.2197	1.9801	0.0000
235810_at	ZNF182	zinc finger protein 182	NM_001007088 /// NM_001178099 /// NM_006962	4.4180	0.9854	0.2230	1.9799	0.0000
204058_at	ME1	malic enzyme 1, NADP(+)-dependent, cytosolic	NM_002395	3.9371	0.9851	0.2502	1.9794	0.0000
203151_at	MAP1A	microtubule-associated protein 1A	NM_002373	5.4723	0.9832	0.1797	1.9769	0.0000
223204_at	FAM198B	family with sequence similarity 198, member B	NM_001031700 /// NM_001128424 /// NM_016613	3.3039	0.9828	0.2975	1.9763	0.0592
1569369_at	ZFYVE28	zinc finger, FYVE domain containing 28	NM_001172656 /// NM_001172657 /// NM_001172658 /// NM_001172659 /// NM_001172660 /// NM_020972	3.2191	0.9819	0.3050	1.9750	0.0852
214374_s_at	PPFIBP1	PTPRF interacting protein, binding protein 1 (liprin beta 1)	NM_003622 /// NM_177444	3.2910	0.9777	0.2971	1.9693	0.0592
207232_s_at	DZIP3	DAZ interacting protein 3, zinc finger	NM_014648	2.7786	0.9771	0.3517	1.9685	0.1046
59437_at	C9orf116	chromosome 9 open reading frame 116	NM_001048265 /// NM_144654	2.9745	0.9771	0.3285	1.9685	0.1046

Table S2. Continued

Probe Set ID	Gene Symbol	Gene Title	RefSeq Transcript ID	Score(d)	Numerat-or(r)	Denominat or(s+s0)	Fold Change	q-value(%)
Up reglulated								
212572_at	STK38L	serine/threonine kinase 38 like	NM_015000	5.9986	0.9763	0.1628	1.9674	0.0000
1570445_a_at	LOC643201	centrosomal protein 192kDa pseudogene	NR_036494	2.9607	0.9757	0.3295	1.9665	0.1046
219287_at	KCNMB4	potassium large conductance calcium-activated channel, subfamily M, beta member 4	NM_014505	2.9644	0.9739	0.3285	1.9642	0.1046
222506_at	LMBR1	limb region 1 homolog (mouse)	NM_022458	2.9086	0.9722	0.3342	1.9618	0.1046
230917_at	NA	NA	NA	3.4383	0.9657	0.2809	1.9531	0.0592
242557_at	NCRNA00171	Non-protein coding RNA 171	NR_026751	4.6237	0.9652	0.2088	1.9524	0.0000
1569459_a_at	NA	NA	NA	4.5811	0.9639	0.2104	1.9505	0.0000
243977_at	LOC541472	hypothetical LOC541472	XR_108749 /// XR_110651 /// XR_112985 /// XR_114001	3.3653	0.9540	0.2835	1.9373	0.0592
229170_s_at	TTC18	tetratricopeptide repeat domain 18	NM_145170	5.2997	0.9539	0.1800	1.9371	0.0000
200696_s_at	GSN	gelsolin	NM_000177 /// NM_001127662 /// NM_001127663 /// NM_001127664 /// NM_001127665 /// NM_001127666 /// NM_001127667 /// NM_198252	3.4950	0.9514	0.2722	1.9338	0.0592

Table S2. Continued

Probe Set ID	Gene Symbol	Gene Title	RefSeq Transcript ID	Score(d)	Numerat-or(r)	Denominator(s+s0)	Fold Change	q-value(%)
Up reglulated								
225008_at	ASPH	aspartate beta-hydroxylase	NM_001164750 /// NM_001164751 /// NM_001164752 /// NM_001164753 /// NM_001164754 /// NM_001164755 /// NM_001164756 /// NM_004318 /// NM_020164 /// NM_032466 /// NM_032467 /// NM_032468	4.4359	0.9400	0.2119	1.9185	0.0000
202083_s_at	SEC14L1	SEC14-like 1 (S. cerevisiae)	NM_001039573 /// NM_001143998 /// NM_001143999 /// NM_001144001 /// NM_003003	2.5906	0.9397	0.3627	1.9181	0.1519
203282_at	GBE1	glucan (1,4-alpha-), branching enzyme 1	NM_000158	2.7366	0.9397	0.3434	1.9181	0.1046
239515_at	NA	NA	NA	3.5965	0.9395	0.2612	1.9178	0.0592
235987_at	PRKXP1	protein kinase, X-linked, pseudogene 1	NA	3.7079	0.9367	0.2526	1.9142	0.0000
218297_at	FAM188A	family with sequence similarity 188, member A	NM_024948	3.8067	0.9357	0.2458	1.9128	0.0000

Table S2. Continued

Probe Set ID	Gene Symbol	Gene Title	RefSeq Transcript ID	Score(d)	Numerat-or(r)	Denominat or(s+s0)	Fold Change	q-value(%)
Up reglulated								
228540_at	QKI	quaking homolog, KH domain RNA binding (mouse)	NM_006775 /// NM_206853 /// NM_206854 /// NM_206855	2.7394	0.9501	0.3468	1.9320	0.1046
209685_s_at	PRKCB	protein kinase C, beta	NM_002738 /// NM_212535	3.8656	0.9497	0.2457	1.9315	0.0000
236545_at	NA	NA	NA	2.5722	0.9497	0.3692	1.9315	0.1519
242268_at	CELF2	CUGBP, Elav-like family member 2	NM_001025076 /// NM_001025077 /// NM_001083591 /// NM_006561	3.0607	0.9496	0.3103	1.9313	0.0852
222881_at	HPSE	heparanase	NM_001098540 /// NM_001166498 /// NM_006665	2.5897	0.9495	0.3666	1.9312	0.1519
225123_at	NA	NA	NA	3.6005	0.9420	0.2616	1.9212	0.0592
201666_at	TIMP1	TIMP metallopeptidase inhibitor 1	NM_003254	5.1373	0.9406	0.1831	1.9193	0.0000
1552988_at	C11orf65	chromosome 11 open reading frame 65	NM_152587	3.9652	0.9404	0.2372	1.9191	0.0000
239021_at	TLR6	toll-like receptor 6	NM_006068	3.9787	0.9403	0.2363	1.9190	0.0000

Table S2. Continued

Probe Set ID	Gene Symbol	Gene Title	RefSeq Transcript ID	Score(d)	Numerat-or(r)	Denominator(s+s0)	Fold Change	q-value(%)
Up reglulated								
222154_s_at	SPATS2L	spermatogenesis associated, serine-rich 2-like	NM_001100422 /// NM_001100423 /// NM_001100424 /// NM_015535	3.7183	0.9356	0.2516	1.9127	0.0000
242224_at	GPATCH2	G patch domain containing 2	NM_018040	4.7492	0.9354	0.1970	1.9124	0.0000
225955_at	METRNL	meteorin, glial cell differentiation regulator-like	NM_001004431	6.9253	0.9321	0.1346	1.9081	0.0000
224996_at	ASPH	aspartate beta-hydroxylase	NM_001164750 /// NM_001164751 /// NM_001164752 /// NM_001164753 /// NM_001164754 /// NM_001164755 /// NM_001164756 /// NM_004318 /// NM_020164 /// NM_032466 /// NM_032467 /// NM_032468	5.0499	0.9268	0.1835	1.9011	0.0000
230927_at	NA	NA	NA	3.3921	0.9263	0.2731	1.9004	0.0592
235871_at	LIPH	lipase, member H	NM_139248	2.7210	0.9257	0.3402	1.8996	0.1046
228696_at	SLC45A3	solute carrier family 45, member 3	NM_033102	5.4952	0.9254	0.1684	1.8992	0.0000
1552971_at	SGCZ	sarcoglycan, zeta	NM_139167	5.3619	0.9251	0.1725	1.8988	0.0000

Table S2. Continued

Probe Set ID	Gene Symbol	Gene Title	RefSeq Transcript ID	Score(d)	Numerat-or(r)	Denominator(s+s0)	Fold Change	q-value(%)
Up reglulated								
237034_at	NA	NA	NA	3.9285	0.9239	0.2352	1.8972	0.0000
1554007_at	NA	NA	NA	5.9350	0.9219	0.1553	1.8946	0.0000
207543_s_at	P4HA1	prolyl 4-hydroxylase, alpha polypeptide I	NM_000917 /// NM_001017962 /// NM_001142595 /// NM_001142596	2.7064	0.9201	0.3400	1.8923	0.1046
201859_at	SRGN	serglycin	NM_002727 /// NR_036430	4.2230	0.9191	0.2176	1.8909	0.0000
219738_s_at	PCDH9	protocadherin 9	NM_020403 /// NM_203487	6.2019	0.9182	0.1481	1.8898	0.0000
226018_at	C7orf41	chromosome 7 open reading frame 41	NM_152793	3.0044	0.9164	0.3050	1.8874	0.1046
212558_at	SPRY1	sprouty homolog 1, antagonist of FGF signaling (Drosophila)	NM_005841 /// NM_199327	3.6185	0.9138	0.2525	1.8840	0.0592
238937_at	ZNF420	zinc finger protein 420	NM_144689	6.0169	0.9138	0.1519	1.8840	0.0000
229968_at	NA	NA	NA	3.6240	0.9138	0.2521	1.8839	0.0592
202872_at	ATP6V1C1	ATPase, H+ transporting, lysosomal 42kDa, V1 subunit C1	NM_001695	5.4002	0.9128	0.1690	1.8826	0.0000
202425_x_at	PPP3CA	protein phosphatase 3, catalytic subunit, alpha isozyme	NM_000944 /// NM_001130691 /// NM_001130692	4.1619	0.9124	0.2192	1.8822	0.0000
218471_s_at	BBS1	Bardet-Biedl syndrome 1	NM_024649	4.9040	0.9123	0.1860	1.8820	0.0000
210560_at	GBX2	gastrulation brain homeobox 2	NM_001485	2.9487	0.9117	0.3092	1.8813	0.1046

Table S2. Continued

Probe Set ID	Gene Symbol	Gene Title	RefSeq Transcript ID	Score(d)	Numerat-or(r)	Denominat or(s+s0)	Fold Change	q-value(%)
Up reglulated								
1556283_s_at	FGFR1OP2	FGFR1 oncogene partner 2	NM_001171887 /// NM_001171888 /// NM_015633	3.7003	0.9104	0.2460	1.8795	0.0000
1567703_at	NA	NA	NA	4.6318	0.9103	0.1965	1.8794	0.0000
227530_at	AKAP12	A kinase (PRKA) anchor protein 12	NM_005100 /// NM_144497	4.2013	0.9086	0.2163	1.8772	0.0000
224959_at	SLC26A2	solute carrier family 26 (sulfate transporter), member 2	NM_000112	3.6119	0.9084	0.2515	1.8769	0.0592
213800_at	CFH	complement factor H	NM_000186 /// NM_001014975	2.5790	0.9047	0.3508	1.8722	0.1519
206805_at	SEMA3A	sema domain, immunoglobulin domain (Ig), short basic domain, secreted, (semaphorin) 3A	NM_006080	5.3034	0.9047	0.1706	1.8721	0.0000
200811_at	CIRBP	cold inducible RNA binding protein	NM_001280 /// NR_023312 /// NR_023313	3.3690	0.9046	0.2685	1.8720	0.0592
209211_at	KLF5	Kruppel-like factor 5 (intestinal)	NM_001730	5.1299	0.9021	0.1759	1.8688	0.0000
225639_at	SKAP2	src kinase associated phosphoprotein 2	NM_003930	5.1473	0.9020	0.1752	1.8686	0.0000
220182_at	SLC25A23	solute carrier family 25 (mitochondrial carrier; phosphate carrier), member 23	NM_024103	3.1916	0.9005	0.2822	1.8668	0.0852
235610_at	ALKBH8	alkB, alkylation repair homolog 8 (E. coli)	NM_138775	4.1507	0.8962	0.2159	1.8612	0.0000
229243_at	NA	NA	NA	3.8958	0.8962	0.2300	1.8611	0.0000
1553494_at	TDH	L-threonine dehydrogenase	NR_001578	2.7643	0.8931	0.3231	1.8571	0.1046

Table S2. Continued

Probe Set ID	Gene Symbol	Gene Title	RefSeq Transcript ID	Score(d)	Numerat-or(r)	Denominat or(s+s0)	Fold Change	q-value(%)
Up reglulated								
227013_at	LATS2	LATS, large tumor suppressor, homolog 2 (Drosophila)	NM_014572	2.5671	0.8911	0.3471	1.8546	0.1519
225651_at	UBE2E2	ubiquitin-conjugating enzyme E2E 2 (UBC4/5 homolog, yeast)	NM_152653	3.7699	0.8911	0.2364	1.8546	0.0000
244393_x_at	NA	NA	NA	2.5823	0.8907	0.3449	1.8540	0.1519
238034_at	CANX	calnexin	NM_001024649 /// NM_001746	3.0362	0.8889	0.2928	1.8518	0.0852
226152_at	TTC7B	tetratricopeptide repeat domain 7B	NM_001010854	2.9667	0.8853	0.2984	1.8471	0.1046
226902_at	USP13	ubiquitin specific peptidase 13 (isopeptidase T-3)	NM_003940	5.1892	0.8831	0.1702	1.8443	0.0000
217955_at	BCL2L13	BCL2-like 13 (apoptosis facilitator)	NM_015367	2.7604	0.8828	0.3198	1.8440	0.1046
208328_s_at	MEF2A	myocyte enhancer factor 2A	NM_001130926 /// NM_001130927 /// NM_001130928 /// NM_001171894 /// NM_005587	4.2109	0.8817	0.2094	1.8425	0.0000
211210_x_at	SH2D1A	SH2 domain containing 1A	NM_001114937 /// NM_002351	3.6025	0.8780	0.2437	1.8378	0.0592
211557_x_at	SLCO2B1	solute carrier organic anion transporter family, member 2B1	NM_001145211 /// NM_001145212 /// NM_007256	2.9234	0.8759	0.2996	1.8351	0.1046
210032_s_at	SPAG6	sperm associated antigen 6	NM_012443 /// NM_172242	2.9077	0.8749	0.3009	1.8339	0.1046

Table S2. Continued

Probe Set ID	Gene Symbol	Gene Title	RefSeq Transcript ID	Score(d)	Numerat-or(r)	Denominator(s+s0)	Fold Change	q-value(%)
Up reglulated								
1556913_a_at	GIT2	G protein-coupled receptor kinase interacting ArfGAP 2	NM_001135213 /// NM_001135214 /// NM_014776 /// NM_057169 /// NM_057170 /// NM_139201	3.3504	0.8715	0.2601	1.8296	0.0592
204499_at	AGTPBP1	ATP/GTP binding protein 1	NM_015239	3.5351	0.8712	0.2464	1.8291	0.0592
208022_s_at	CDC14B	CDC14 cell division cycle 14 homolog B (S. cerevisiae)	NM_001077181 /// NM_003671 /// NM_033331	2.8932	0.8711	0.3011	1.8291	0.1046
237675_at	NA	NA	NA	3.2258	0.8698	0.2696	1.8274	0.0852
226099_at	ELL2	elongation factor, RNA polymerase II, 2	NM_012081	4.3620	0.8694	0.1993	1.8269	0.0000
227278_at	NA	NA	NA	2.7752	0.8662	0.3121	1.8228	0.1046
229958_at	CLN8	ceroid-lipofuscinosis, neuronal [illegible] (epilepsy, progressive with mental retardation)	NM_018941	3.6493	0.8659	0.2373	1.8224	0.0000
203814_s_at	NQO2	NAD(P)H dehydrogenase, quinone 2	NM_000904	3.9856	0.8644	0.2169	1.8205	0.0000
210976_s_at	PFKM	phosphofructokinase, muscle	NM_000289 /// NM_001166686 /// NM_001166687 /// NM_001166688	4.4913	0.8627	0.1921	1.8184	0.0000
228908_s_at	LOC642852	hypothetical LOC642852	NR_026943	6.4301	0.8602	0.1338	1.8153	0.0000

Table S2. Continued

Probe Set ID	Gene Symbol	Gene Title	RefSeq Transcript ID	Score(d)	Numerat-or(r)	Denominat or(s+s0)	Fold Change	q-value(%)
Up reglulated								
225146_at	C9orf25	chromosome 9 open reading frame 25	NM_001184940 /// NM_001184941 /// NM_001184942 /// NM_001184943 /// NM_001184944 /// NM_001184945 /// NM_147202	5.1208	0.8589	0.1677	1.8137	0.0000
205278_at	GAD1	glutamate decarboxylase 1 (brain, 67kDa)	NM_000817 /// NM_013445	3.3231	0.8561	0.2576	1.8102	0.0592
235333_at	B4GALT6	UDP-Gal:betaGlcNAc beta 1,4-galactosyltransferase, polypeptide 6	NM_004775	3.6865	0.8556	0.2321	1.8095	0.0000
223609_at	ROPN1L	ropporin 1-like	NM_031916	3.0840	0.8553	0.2773	1.8092	0.0852
209264_s_at	TSPAN4	tetraspanin 4	NM_001025234 /// NM_001025235 /// NM_001025236 /// NM_001025237 /// NM_001025238 /// NM_001025239 /// NM_003271	2.9915	0.8538	0.2854	1.8072	0.1046
225502_at	DOCK8	dedicator of cytokinesis 8	NM_001190458 /// NM_001193536 /// NM_203447	4.9341	0.8538	0.1730	1.8072	0.0000

Table S2. Continued

Probe Set ID	Gene Symbol	Gene Title	RefSeq Transcript ID	Score(d)	Numerat-or(r)	Denominator(s+s0)	Fold Change	q-value(%)
Up reglulated								
208868_s_at	GABARAPL 1	GABA(A) receptor-associated protein like 1	NM_031412	3.0103	0.8530	0.2833	1.8062	0.1046
1555037_a_at	IDH1	isocitrate dehydrogenase 1 (NADP+), soluble	NM_005896	5.3482	0.8529	0.1595	1.8061	0.0000
235392_at	NA	NA	NA	3.7914	0.8527	0.2249	1.8059	0.0000
1558953_s_at	CEP164	centrosomal protein 164kDa	NM_014956	4.9970	0.8522	0.1705	1.8052	0.0000
214724_at	DIXDC1	DIX domain containing 1	NM_001037954 /// NM_033425	2.9945	0.8506	0.2841	1.8033	0.1046
223405_at	NPL	N-acetylneuraminate pyruvate lyase (dihydrodipicolinate synthase)	NM_030769	3.8880	0.8505	0.2188	1.8032	0.0000
204613_at	PLCG2	phospholipase C, gamma 2 (phosphatidylinositol-specific)	NM_002661	3.6566	0.8490	0.2322	1.8012	0.0000
229228_at	CREB5	cAMP responsive element binding protein 5	NM_001011666 /// NM_004904 /// NM_182898 /// NM_182899	3.4298	0.8486	0.2474	1.8007	0.0592
202856_s_at	SLC16A3	solute carrier family 16, member 3 (monocarboxylic acid transporter 4)	NM_001042422 /// NM_001042423 /// NM_004207	2.5899	0.8468	0.3270	1.7985	0.1519
221029_s_at	WNT5B	wingless-type MMTV integration site family, member 5B	NM_030775 /// NM_032642	4.2701	0.8444	0.1978	1.7956	0.0000
223380_s_at	LATS2	LATS, large tumor suppressor, homolog 2 (Drosophila)	NM_014572	2.5551	0.8444	0.3305	1.7955	0.1519

Table S2. Continued

Probe Set ID	Gene Symbol	Gene Title	RefSeq Transcript ID	Score(d)	Numerat-or(r)	Denominat or(s+s0)	Fold Change	q-value(%)
Up reglulated								
218209_s_at	RPRD1A	regulation of nuclear pre-mRNA domain containing 1A	NM_018170	6.5913	0.8443	0.1281	1.7954	0.0000
222402_at	POMP	proteasome maturation protein	NM_015932	4.3719	0.8425	0.1927	1.7932	0.0000
227226_at	MRAP2	melanocortin 2 receptor accessory protein 2	NM_138409	5.5248	0.8415	0.1523	1.7919	0.0000
205097_at	SLC26A2	solute carrier family 26 (sulfate transporter), member 2	NM_000112	3.0125	0.8403	0.2790	1.7905	0.1046
243762_at	NA	NA	NA	2.7524	0.8398	0.3051	1.7898	0.1046
202962_at	KIF13B	kinesin family member 13B	NM_015254	3.0667	0.8393	0.2737	1.7892	0.0852
204255_s_at	VDR	vitamin D (1,25- dihydroxyvitamin D3) receptor	NM_000376 /// NM_001017535	2.8392	0.8388	0.2954	1.7886	0.1046
229465_s_at	NA	NA	NA	3.5351	0.8378	0.2370	1.7874	0.0592
213552_at	GLCE	glucuronic acid epimerase	NM_015554	3.8485	0.8363	0.2173	1.7855	0.0000
218775_s_at	WWC2	WW and C2 domain containing 2	NM_024949	3.4139	0.8347	0.2445	1.7835	0.0592
242418_at	NA	NA	NA	2.8534	0.8346	0.2925	1.7834	0.1046
233329_s_at	KRCC1	lysine-rich coiled-coil 1	NM_016618	3.7544	0.8335	0.2220	1.7820	0.0000
207057_at	SLC16A7	solute carrier family 16, member 7 (monocarboxylic acid transporter 2)	NM_004731	2.6480	0.8332	0.3146	1.7816	0.1046
238098_at	NA	NA	NA	3.0864	0.8330	0.2699	1.7814	0.0852
235899_at	CA13	carbonic anhydrase XIII	NM_198584	3.5245	0.8311	0.2358	1.7790	0.0592

Table S2. Continued

Probe Set ID	Gene Symbol	Gene Title	RefSeq Transcript ID	Score(d)	Numerat-or(r)	Denominat or(s+s0)	Fold Change	q-value(%)
Up reglulated								
200924_s_at	SLC3A2	solute carrier family 3 (activators of dibasic and neutral amino acid transport), member 2	NM_001012661 /// NM_001012662 /// NM_001012663 /// NM_001012664 /// NM_001013251 /// NM_002394	3.3758	0.8308	0.2461	1.7787	0.0592
223386_at	FAM118B	family with sequence similarity 118, member B	NM_024556	2.8421	0.8297	0.2919	1.7773	0.1046
212509_s_at	MXRA7	matrix-remodelling associated 7	NM_001008528 /// NM_001008529 /// NM_198530	3.6072	0.8283	0.2296	1.7755	0.0592
223551_at	PKIB	protein kinase (cAMP-dependent, catalytic) inhibitor beta	NM_032471 /// NM_181794 /// NM_181795	3.6000	0.8262	0.2295	1.7730	0.0592
209827_s_at	IL16	interleukin 16 (lymphocyte chemoattractant factor)	NM_001172128 /// NM_004513 /// NM_172217	3.9552	0.8261	0.2089	1.7729	0.0000
226435_at	PAPLN	papilin, proteoglycan-like sulfated glycoprotein	NM_173462	3.1083	0.8240	0.2651	1.7703	0.0852
238635_at	C5orf28	chromosome 5 open reading frame 28	NM_022483	3.2292	0.8232	0.2549	1.7693	0.0852
231557_at	NA	NA	NA	3.0356	0.8194	0.2699	1.7646	0.0852
205837_s_at	GYPA	glycophorin A (MNS blood group)	NM_002099	3.2489	0.8193	0.2522	1.7645	0.0592

Table S2. Continued

Probe Set ID	Gene Symbol	Gene Title	RefSeq Transcript ID	Score(d)	Numerat-or(r)	Denominator(s+s0)	Fold Change	q-value(%)
Up reglulated								
204484_at	PIK3C2B	phosphoinositide-3-kinase, class 2, beta polypeptide	NM_002646	3.4478	0.8191	0.2376	1.7643	0.0592
228667_at	AGPAT4	1-acylglycerol-3-phosphate O-acyltransferase 4 (lysophosphatidic acid acyltransferase, delta)	NM_020133	3.1199	0.8184	0.2623	1.7635	0.0852
228867_at	TATDN3	TatD DNase domain containing 3	NM_001042552 /// NM_001042553 /// NM_001146169 /// NM_001146170 /// NM_001146171	3.8954	0.8174	0.2098	1.7622	0.0000
228700_at	CXorf38	chromosome X open reading frame 38	NM_144970	3.8631	0.8168	0.2114	1.7615	0.0000
240201_at	NA	NA	NA	5.6880	0.8166	0.1436	1.7612	0.0000
226050_at	TMCO3	transmembrane and coiled-coil domains 3	NM_017905	3.4017	0.8160	0.2399	1.7605	0.0592
219079_at	CYB5R4	cytochrome b5 reductase 4	NM_016230	4.4158	0.8147	0.1845	1.7589	0.0000
226501_at	XPNPEP3	X-prolyl aminopeptidase (aminopeptidase P) 3, putative	NM_022098	4.2704	0.8101	0.1897	1.7533	0.0000
213608_s_at	SRRD	SRR1 domain containing	NM_001013694	3.9457	0.8093	0.2051	1.7523	0.0000
Down regulated								
1552304_at	ALG10	asparagine-linked glycosylation 10, alpha-1,2-glucosyltransferase homolog (S. pombe)	NM_032834	-4.7596	-0.8712	0.1830	0.5467	0.0000
1552365_at	SCIN	scinderin	NM_001112706 /// NM_033128	-4.4648	-2.9770	0.6668	0.1270	0.0000

Table S2. Continued

Probe Set ID	Gene Symbol	Gene Title	RefSeq Transcript ID	Score(d)	Numerat-or(r)	Denominator(s+s0)	Fold Change	q-value(%)
Down reglulated								
1552367_a_at	SCIN	scinderin	NM_001112706 /// NM_033128	-4.8119	-2.0817	0.4326	0.2362	0.0000
1552519_at	ACVR1C	activin A receptor, type IC	NM_001111031 /// NM_001111032 /// NM_001111033 /// NM_145259	-5.1838	-1.8258	0.3522	0.2821	0.0000
1552682_a_at	CASC5	cancer susceptibility candidate 5	NM_144508 /// NM_170589	-3.8085	-1.2073	0.3170	0.4331	0.0000
1552906_at	FMR1NB	fragile X mental retardation 1 neighbor	NM_152578	-4.2196	-1.6245	0.3850	0.3243	0.0000
1552912_a_at	IL23R	interleukin 23 receptor	NM_144701	-3.4880	-1.4964	0.4290	0.3544	0.0000
1552927_at	TAB3	TGF-beta activated kinase 1/MAP3K7 binding protein 3	NM_152787	-3.1934	-0.8861	0.2775	0.5411	0.0622
1552928_s_at	TAB3	TGF-beta activated kinase 1/MAP3K7 binding protein 3	NM_152787	-4.2027	-1.1053	0.2630	0.4648	0.0000
1553102_a_at	CCDC69	coiled-coil domain containing 69	NM_015621	-9.1461	-2.3008	0.2516	0.2030	0.0000
1553132_a_at	TC2N	tandem C2 domains, nuclear	NM_001128595 /// NM_001128596 /// NM_152332	-5.8402	-2.7460	0.4702	0.1491	0.0000
1553219_a_at	AMMECR1	Alport syndrome, mental retardation, midface hypoplasia and elliptocytosis chromosomal region gene 1	NM_001025580 /// NM_001171689 /// NM_015365	-2.5795	-0.8244	0.3196	0.5647	0.1081
1553244_at	FANCB	Fanconi anemia, complementation group B	NM_001018113 /// NM_152633	-6.0593	-1.0877	0.1795	0.4705	0.0000

Table S2. Continued

Probe Set ID	Gene Symbol	Gene Title	RefSeq Transcript ID	Score(d)	Numerat-or(r)	Denominator(s+s0)	Fold Change	q-value(%)
Down reglulated								
1553587_a_at	POLE4	polymerase (DNA-directed), epsilon 4 (p12 subunit)	NM_019896	-5.2931	-1.0004	0.1890	0.4999	0.0000
1553604_at	ABCA13	ATP-binding cassette, sub-family A (ABC1), member 13	NM_152701	-3.0634	-1.2349	0.4031	0.4249	0.0622
1553605_a_at	ABCA13	ATP-binding cassette, sub-family A (ABC1), member 13	NM_152701	-6.8380	-2.9602	0.4329	0.1285	0.0000
1553696_s_at	ZNF569	zinc finger protein 569	NM_152484	-15.3284	-2.3285	0.1519	0.1991	0.0000
1553815_a_at	TCEANC	transcription elongation factor A (SII) N-terminal and central domain containing	NM_152634	-3.4507	-1.4671	0.4252	0.3617	0.0000
1554248_at	ZNF638	zinc finger protein 638	NM_001014972 /// NM_014497	-3.1765	-0.8178	0.2575	0.5673	0.0622
1554486_a_at	C6orf114	chromosome 6 open reading frame 114	NM_033069	-3.5794	-1.2661	0.3537	0.4158	0.0000
1554493_s_at	THADA	thyroid adenoma associated	NM_001083953 /// NM_022065	-3.2952	-0.8428	0.2558	0.5576	0.0000
1554609_at	LOC100287896	Similar to cag	XM_002343118 /// XM_003119510 /// XM_003120708	-3.8041	-0.9461	0.2487	0.5190	0.0000
1554821_a_at	ZBED1	zinc finger, BED-type containing 1	NM_001171135 /// NM_001171136 /// NM_004729	-6.3036	-0.9607	0.1524	0.5138	0.0000
1554830_a_at	STEAP3	STEAP family member 3	NM_001008410 /// NM_018234 /// NM_182915	-5.9562	-1.4262	0.2394	0.3721	0.0000

Table S2. Continued

Probe Set ID	Gene Symbol	Gene Title	RefSeq Transcript ID	Score(d)	Numerat-or(r)	Denominat or(s+s0)	Fold Change	q-value(%)
Down reglulated								
1554835_a_at	B3GNT5	UDP-GlcNAc:betaGal beta-1,3-N-acetylglucosaminyltransferase 5	NM_032047	-4.1260	-0.8203	0.1988	0.5663	0.0000
1555120_at	CD96	CD96 molecule	NM_005816 /// NM_198196	-15.9413	-3.2905	0.2064	0.1022	0.0000
1555357_at	DDX53	DEAD (Asp-Glu-Ala-Asp) box polypeptide 53	NM_182699	-3.5903	-0.9958	0.2774	0.5015	0.0000
1555538_s_at	FAM9B	family with sequence similarity 9, member B	NM_205849	-10.5763	-2.2170	0.2096	0.2151	0.0000
1555728_a_at	MS4A4A	membrane-spanning 4-domains, subfamily A, member 4	NM_024021 /// NM_148975	-6.5094	-2.3631	0.3630	0.1944	0.0000
1555731_a_at	AP1S3	adaptor-related protein complex 1, sigma 3 subunit	NM_001039569	-6.6625	-1.5494	0.2326	0.3417	0.0000
1555733_s_at	AP1S3	adaptor-related protein complex 1, sigma 3 subunit	NM_001039569	-3.4185	-0.8369	0.2448	0.5598	0.0000
1555745_a_at	LYZ	lysozyme	NM_000239	-8.3551	-1.3243	0.1585	0.3993	0.0000
1555793_a_at	ZFP82	zinc finger protein 82 homolog (mouse)	NM_133466	-10.7255	-1.9339	0.1803	0.2617	0.0000
1555812_a_at	ARHGDIB	Rho GDP dissociation inhibitor (GDI) beta	NM_001175	-4.9037	-0.9034	0.1842	0.5346	0.0000
1555829_at	ESYT2	extended synaptotagmin-like protein 2	NM_020728	-5.8618	-1.5029	0.2564	0.3528	0.0000
1555864_s_at	PDHA1	pyruvate dehydrogenase (lipoamide) alpha 1	NM_000284 /// NM_001173454 /// NM_001173455 /// NM_001173456	-11.0780	-1.3044	0.1178	0.4049	0.0000
1556033_at	FLJ39739	hypothetical FLJ39739	NR_027468	-10.5832	-2.4748	0.2338	0.1799	0.0000

Table S2. Continued

Probe Set ID	Gene Symbol	Gene Title	RefSeq Transcript ID	Score(d)	Numerat-or(r)	Denominator(s+s0)	Fold Change	q-value(%)
Down reglulated								
1556195_a_at	NA	NA	NA	-3.9509	-0.9020	0.2283	0.5351	0.0000
1556201_at	RNASET2	ribonuclease T2	NM_003730	-3.8768	-1.0325	0.2663	0.4889	0.0000
1556236_at	NA	NA	NA	-5.2186	-1.2464	0.2388	0.4215	0.0000
1556613_s_at	DPY19L4	dpy-19-like 4 (C. elegans)	NM_181787	-4.7175	-1.0363	0.2197	0.4876	0.0000
1557137_at	TMEM17	transmembrane protein 17	NM_198276	-4.0367	-1.1344	0.2810	0.4555	0.0000
1557155_a_at	NA	NA	NA	-7.4165	-1.1314	0.1525	0.4565	0.0000
1557167_at	HCG11	HLA complex group 11	NR_026790	-3.7704	-0.8666	0.2298	0.5484	0.0000
1557169_x_at	HCG11	HLA complex group 11	NR_026790	-5.3395	-0.9452	0.1770	0.5194	0.0000
1557174_a_at	NA	NA	NA	-3.3721	-1.1139	0.3303	0.4620	0.0000
1557217_a_at	FANCB	Fanconi anemia, complementation group B	NM_001018113 /// NM_152633	-4.5920	-1.1722	0.2553	0.4437	0.0000
1557218_s_at	FANCB	Fanconi anemia, complementation group B	NM_001018113 /// NM_152633	-5.7189	-0.9592	0.1677	0.5143	0.0000
1557261_at	WHAMML1 /// WHAMML2	WAS protein homolog associated with actin, golgi membranes and microtubules-like 1 (pseudogene) /// WAS protein homolog associated with actin, golgi membranes and microtubules-like 2 (pseudogene)	NR_003521 /// NR_026589	-4.5848	-1.4186	0.3094	0.3741	0.0000
1557411_s_at	SLC25A43	solute carrier family 25, member 43	NM_145305	-13.6023	-3.0977	0.2277	0.1168	0.0000
1557954_at	TXLNG	taxilin gamma	NM_001168683 /// NM_018360	-4.9237	-1.2044	0.2446	0.4340	0.0000
1558166_at	MGC16275	hypothetical protein MGC16275	NR_026914	-3.9830	-1.0028	0.2518	0.4990	0.0000

Table S2. Continued

Probe Set ID	Gene Symbol	Gene Title	RefSeq Transcript ID	Score(d)	Numerat-or(r)	Denominator(s+s0)	Fold Change	q-value(%)
Down reglulated								
1558201_s_at	SLC4A1AP	solute carrier family 4 (anion exchanger), member 1, adaptor protein	NM_018158	-10.2927	-1.3597	0.1321	0.3897	0.0000
1558216_at	AFAP1-AS	AFAP1 antisense RNA (non-protein coding)	NR_026892	-6.9535	-1.8399	0.2646	0.2793	0.0000
1558290_a_at	PVT1	Pvt1 oncogene (non-protein coding)	NR_003367	-9.8921	-1.6417	0.1660	0.3205	0.0000
1558508_a_at	C1orf53	chromosome 1 open reading frame 53	NM_001024594	-3.0366	-0.8582	0.2826	0.5516	0.0622
1558896_at	C1orf69	chromosome 1 open reading frame 69	NM_001010867	-3.8450	-0.8601	0.2237	0.5509	0.0000
1559705_s_at	PHKA2	phosphorylase kinase, alpha 2 (liver)	NM_000292	-4.2122	-1.1527	0.2737	0.4498	0.0000
1559776_at	NA	NA	NA	-3.0490	-1.6531	0.5422	0.3180	0.0622
1561180_at	NA	NA	NA	-3.2938	-1.0088	0.3063	0.4970	0.0000
1561721_a_at	C12orf40	chromosome 12 open reading frame 40	NM_001031748	-10.5829	-3.2873	0.3106	0.1024	0.0000
1561757_a_at	LOC283352	hypothetical protein LOC283352	NA	-27.8989	-4.1326	0.1481	0.0570	0.0000
1561853_a_at	IL23R	interleukin 23 receptor	NM_144701	-2.7298	-1.3954	0.5112	0.3801	0.0852
1562337_at	OR7D2	olfactory receptor, family 7, subfamily D, member 2	NM_175883	-2.8261	-0.9565	0.3384	0.5153	0.0622
1563357_at	NA	NA	NA	-2.7490	-1.3820	0.5027	0.3837	0.0852
1564220_a_at	NA	NA	NA	-8.4707	-2.1154	0.2497	0.2308	0.0000
1566764_at	NA	NA	NA	-3.0351	-1.1317	0.3729	0.4564	0.0622
1567183_s_at	NA	NA	NA	-3.4661	-0.8294	0.2393	0.5628	0.0000

Table S2. Continued

Probe Set ID	Gene Symbol	Gene Title	RefSeq Transcript ID	Score(d)	Numerat-or(r)	Denominat or(s+s0)	Fold Change	q-value(%)
Down reglulated								
1568609_s_at	FAM91A2 /// FLJ39739 /// LOC100286793 /// LOC728855 /// LOC728875	family with sequence similarity 91, member A2 /// hypothetical FLJ39739 /// hypothetical LOC100286793 /// hypothetical LOC728855 /// hypothetical LOC728875	NR_024510 /// NR_024511 /// NR_024584 /// NR_027468 /// NR_027469 /// XR_040635	-2.4254	-1.0741	0.4428	0.4750	0.1081
1568619_s_at	ITPRIPL2	inositol 1,4,5-triphosphate receptor interacting protein-like 2	NM_001034841 /// NR_028028	-2.5060	-0.9552	0.3812	0.5158	0.1081
1568640_at	LOC400960	Hypothetical gene supported by BC040598	NR_033872	-5.5125	-1.0527	0.1910	0.4821	0.0000
1568933_at	LOC646627	phospholipase inhibitor	NM_001085474	-4.2964	-1.1259	0.2620	0.4582	0.0000
1569366_a_at	ZNF569	zinc finger protein 569	NM_152484	-3.3030	-1.4634	0.4431	0.3626	0.0000
1569454_a_at	LOC283352	hypothetical protein LOC283352	NA	-18.9957	-5.6772	0.2989	0.0195	0.0000
1569629_x_at	LOC389906	hypothetical LOC389906	NR_034031	-3.9975	-1.1411	0.2855	0.4534	0.0000
1569867_at	EME2	essential meiotic endonuclease 1 homolog 2 (S. pombe)	NM_001010865	-2.1898	-0.8891	0.4060	0.5400	0.1519
1569878_at	CCNYL2	cyclin Y-like 2	XR_110546 /// XR_111061 /// XR_114355	-4.9150	-1.3424	0.2731	0.3944	0.0000
1569886_a_at	GLB1L3	galactosidase, beta 1-like 3	NM_001080407	-8.2770	-2.2834	0.2759	0.2054	0.0000
1569955_at	NA	NA	NA	-4.9293	-1.4382	0.2918	0.3690	0.0000

Table S2. Continued

Probe Set ID	Gene Symbol	Gene Title	RefSeq Transcript ID	Score(d)	Numerat-or(r)	Denominator(s+s0)	Fold Change	q-value(%)
Down reglulated								
200621_at	CSRP1	cysteine and glycine-rich protein 1	NM_001193570 /// NM_001193571 /// NM_001193572 /// NM_004078	-5.7164	-1.3995	0.2448	0.3791	0.0000
200665_s_at	SPARC	secreted protein, acidic, cysteine-rich (osteonectin)	NM_003118	-9.3684	-2.8070	0.2996	0.1429	0.0000
200824_at	GSTP1	glutathione S-transferase pi 1	NM_000852	-6.0462	-0.8734	0.1445	0.5458	0.0000
200878_at	EPAS1	endothelial PAS domain protein 1	NM_001430	-7.7100	-1.6646	0.2159	0.3154	0.0000
200923_at	LGALS3BP	lectin, galactoside-binding, soluble, 3 binding protein	NM_005567	-2.6986	-0.9081	0.3365	0.5329	0.0852
200979_at	PDHA1	pyruvate dehydrogenase (lipoamide) alpha 1	NM_000284 /// NM_001173454 /// NM_001173455 /// NM_001173456	-6.3536	-1.8066	0.2843	0.2859	0.0000
200980_s_at	PDHA1	pyruvate dehydrogenase (lipoamide) alpha 1	NM_000284 /// NM_001173454 /// NM_001173455 /// NM_001173456	-11.4379	-1.4007	0.1225	0.3788	0.0000
201017_at	EIF1AX	eukaryotic translation initiation factor 1A, X-linked	NM_001412	-4.2210	-1.0286	0.2437	0.4902	0.0000
201042_at	TGM2	transglutaminase 2 (C polypeptide, protein-glutamine-gamma-glutamyltransferase)	NM_004613 /// NM_198951	-2.5915	-0.9004	0.3474	0.5357	0.1081

Table S2. Continued

Probe Set ID	Gene Symbol	Gene Title	RefSeq Transcript ID	Score(d)	Numerat-or(r)	Denominator(s+s0)	Fold Change	q-value(%)
Down reglulated								
201092_at	RBBP7	retinoblastoma binding protein 7	NM_001198719 /// NM_002893	-9.0806	-1.4283	0.1573	0.3716	0.0000
201188_s_at	ITPR3	inositol 1,4,5-triphosphate receptor, type 3	NM_002224	-4.1479	-1.0188	0.2456	0.4935	0.0000
201189_s_at	ITPR3	inositol 1,4,5-triphosphate receptor, type 3	NM_002224	-4.7140	-1.0380	0.2202	0.4870	0.0000
201212_at	LGMN	legumain	NM_001008530 /// NM_005606	-2.2932	-1.0930	0.4766	0.4688	0.1081
201272_at	AKR1B1	aldo-keto reductase family 1, member B1 (aldose reductase)	NM_001628	-9.9866	-3.9726	0.3978	0.0637	0.0000
201278_at	DAB2	disabled homolog 2, mitogen-responsive phosphoprotein (Drosophila)	NM_001343	-7.3299	-1.1580	0.1580	0.4481	0.0000
201279_s_at	DAB2	disabled homolog 2, mitogen-responsive phosphoprotein (Drosophila)	NM_001343	-4.3575	-1.1498	0.2639	0.4507	0.0000
201280_s_at	DAB2	disabled homolog 2, mitogen-responsive phosphoprotein (Drosophila)	NM_001343	-2.6787	-1.1165	0.4168	0.4612	0.0852
201288_at	ARHGDIB	Rho GDP dissociation inhibitor (GDI) beta	NM_001175	-6.5296	-1.3811	0.2115	0.3839	0.0000
201315_x_at	IFITM2	interferon induced transmembrane protein 2 (1-8D)	NM_006435	-3.1126	-1.0306	0.3311	0.4895	0.0622
201427_s_at	SEPP1	selenoprotein P, plasma, 1	NM_001085486 /// NM_001093726 /// NM_005410	-22.2715	-6.4594	0.2900	0.0114	0.0000

Table S2. Continued

Probe Set ID	Gene Symbol	Gene Title	RefSeq Transcript ID	Score(d)	Numerat-or(r)	Denominator(s+s0)	Fold Change	q-value(%)
Down reglulated								
201449_at	TIA1	TIA1 cytotoxic granule-associated RNA binding protein	NM_022037 /// NM_022173	-2.2992	-0.8150	0.3545	0.5684	0.1081
201502_s_at	NFKBIA	nuclear factor of kappa light polypeptide gene enhancer in B-cells inhibitor, alpha	NM_020529	-5.3854	-0.8846	0.1643	0.5416	0.0000
201508_at	IGFBP4	insulin-like growth factor binding protein 4	NM_001552	-4.9747	-1.0261	0.2063	0.4910	0.0000
201622_at	SND1	staphylococcal nuclease and tudor domain containing 1	NM_014390	-6.9784	-0.9970	0.1429	0.5011	0.0000
201650_at	KRT19	keratin 19	NM_002276	-3.3401	-1.2323	0.3690	0.4256	0.0000
201669_s_at	MARCKS	myristoylated alanine-rich protein kinase C substrate	NM_002356	-4.9350	-1.3773	0.2791	0.3849	0.0000
201801_s_at	SLC29A1	solute carrier family 29 (nucleoside transporters), member 1	NM_001078174 /// NM_001078175 /// NM_001078176 /// NM_001078177 /// NM_004955	-4.2169	-0.8722	0.2068	0.5463	0.0000
201850_at	CAPG	capping protein (actin filament), gelsolin-like	NM_001747	-8.3902	-1.7692	0.2109	0.2934	0.0000
201868_s_at	TBL1X	transducin (beta)-like 1X-linked	NM_001139466 /// NM_001139467 /// NM_001139468 /// NM_005647	-2.7420	-0.8260	0.3013	0.5641	0.0852
201876_at	PON2	paraoxonase 2	NM_000305 /// NM_001018161	-18.5892	-4.5527	0.2449	0.0426	0.0000

Table S2. Continued

Probe Set ID	Gene Symbol	Gene Title	RefSeq Transcript ID	Score(d)	Numerat-or(r)	Denominator(s+s0)	Fold Change	q-value(%)
Down reglulated								
201889_at	FAM3C	family with sequence similarity 3, member C	NM_001040020 /// NM_014888	-4.2401	-0.8915	0.2102	0.5391	0.0000
201904_s_at	CTDSPL	CTD (carboxy-terminal domain, RNA polymerase II, polypeptide A) small phosphatase-like	NM_001008392 /// NM_005808	-6.3187	-1.9417	0.3073	0.2603	0.0000
201905_s_at	CTDSPL	CTD (carboxy-terminal domain, RNA polymerase II, polypeptide A) small phosphatase-like	NM_001008392 /// NM_005808	-5.8349	-1.3826	0.2370	0.3835	0.0000
201906_s_at	CTDSPL	CTD (carboxy-terminal domain, RNA polymerase II, polypeptide A) small phosphatase-like	NM_001008392 /// NM_005808	-13.9626	-2.1000	0.1504	0.2333	0.0000
202068_s_at	LDLR	low density lipoprotein receptor	NM_000527 /// NM_001195798 /// NM_001195799 /// NM_001195800 /// NM_001195802 /// NM_001195803	-7.2691	-0.8709	0.1198	0.5468	0.0000
202092_s_at	ARL2BP	ADP-ribosylation factor-like 2 binding protein	NM_012106	-4.7322	-0.9684	0.2046	0.5111	0.0000
202119_s_at	CPNE3	copine III	NM_003909	-3.2490	-0.8638	0.2659	0.5495	0.0000
202260_s_at	STXBP1	syntaxin binding protein 1	NM_001032221 /// NM_003165	-5.5435	-0.8730	0.1575	0.5460	0.0000

Table S2. Continued

Probe Set ID	Gene Symbol	Gene Title	RefSeq Transcript ID	Score(d)	Numerat-or(r)	Denominat or(s+s0)	Fold Change	q-value(%)
Down reglulated								
202481_at	DHRS3	dehydrogenase/reductase (SDR family) member 3	NM_004753	-3.9418	-0.9501	0.2410	0.5176	0.0000
202581_at	HSPA1A /// HSPA1B	heat shock 70kDa protein 1A /// heat shock 70kDa protein 1B	NM_005345 /// NM_005346	-3.4957	-1.8743	0.5362	0.2728	0.0000
202636_at	RNF103	ring finger protein 103	NM_005667	-3.6636	-0.9331	0.2547	0.5237	0.0000
202651_at	LPGAT1	lysophosphatidylglycerol acyltransferas 1	NM_014873	-5.2906	-0.9000	0.1701	0.5359	0.0000
202656_s_at	SERTAD2	SERTA domain containing 2	NM_014755	-2.9503	-0.8476	0.2873	0.5557	0.0622
202657_s_at	SERTAD2	SERTA domain containing 2	NM_014755	-4.1628	-0.9330	0.2241	0.5238	0.0000
202712_s_at	CKMT1A /// CKMT1B	creatine kinase, mitochondrial 1A /// creatine kinase, mitochondrial 1B	NM_001015001 /// NM_020990	-8.7315	-2.8485	0.3262	0.1388	0.0000
202813_at	TARBP1	TAR (HIV-1) RNA binding protein 1	NM_005646	-3.8947	-0.9638	0.2475	0.5127	0.0000
202838_at	FUCA1	fucosidase, alpha-L- 1, tissue	NM_000147	-4.4676	-1.2936	0.2896	0.4079	0.0000
202902_s_at	CTSS	cathepsin S	NM_004079	-6.1539	-2.0499	0.3331	0.2415	0.0000
202905_x_at	NBN	nibrin	NM_002485	-4.4493	-0.9871	0.2219	0.5045	0.0000
202906_s_at	NBN	nibrin	NM_002485	-5.8594	-0.9675	0.1651	0.5114	0.0000
202907_s_at	NBN	nibrin	NM_002485	-5.4521	-0.9320	0.1709	0.5241	0.0000
202957_at	HCLS1	hematopoietic cell-specific Lyn substrate 1	NM_005335	-9.9434	-2.0185	0.2030	0.2468	0.0000
202993_at	ILVBL	ilvB (bacterial acetolactate synthase)-like	NM_006844	-5.3607	-1.0574	0.1973	0.4805	0.0000
203043_at	ZBED1	zinc finger, BED-type containing 1	NM_001171135 /// NM_001171136 /// NM_004729	-4.2778	-0.9316	0.2178	0.5243	0.0000

Table S2. Continued

Probe Set ID	Gene Symbol	Gene Title	RefSeq Transcript ID	Score(d)	Numerat-or(r)	Denominator(s+s0)	Fold Change	q-value(%)
Down reglulated								
203065_s_at	CAV1	caveolin 1, caveolae protein, 22kDa	NM_001172895 /// NM_001172896 /// NM_001172897 /// NM_001753	-4.5424	-1.7941	0.3950	0.2884	0.0000
203126_at	IMPA2	inositol(myo)-1(or 4)-monophosphatase 2	NM_014214	-5.0012	-1.7959	0.3591	0.2880	0.0000
203139_at	DAPK1	death-associated protein kinase 1	NM_004938	-8.7894	-2.4738	0.2814	0.1800	0.0000
203143_s_at	KIAA0040	KIAA0040	NM_001162893 /// NM_001162894 /// NM_001162895 /// NM_014656	-7.1043	-1.6138	0.2272	0.3267	0.0000
203314_at	GTPBP6 /// LOC100508214 /// LOC100510565	GTP binding protein 6 (putative) /// putative GTP-binding protein 6-like /// putative GTP-binding protein 6-like	NM_012227 /// XM_003119188 /// XM_003119189 /// XM_003119198 /// XM_003119199 /// XM_003120137 /// XM_003121024	-4.2666	-0.8887	0.2083	0.5401	0.0000
203315_at	NCK2	NCK adaptor protein 2	NM_001004720 /// NM_001004722 /// NM_003581	-10.6472	-1.4395	0.1352	0.3687	0.0000
203317_at	PSD4	pleckstrin and Sec7 domain containing 4	NM_012455	-4.3897	-0.8498	0.1936	0.5549	0.0000
203323_at	CAV2	caveolin 2	NM_001233 /// NM_198212	-5.2194	-1.1218	0.2149	0.4595	0.0000

Table S2. Continued

Probe Set ID	Gene Symbol	Gene Title	RefSeq Transcript ID	Score(d)	Numerat-or(r)	Denominator(s+s0)	Fold Change	q-value(%)
Down reglulated								
203324_s_at	CAV2	caveolin 2	NM_001233 /// NM_198212	-4.3867	-0.8101	0.1847	0.5703	0.0000
203336_s_at	ITGB1BP1	integrin beta 1 binding protein 1	NM_004763 /// NM_022334	-6.7658	-0.9201	0.1360	0.5285	0.0000
203381_s_at	APOE	apolipoprotein E	NM_000041	-3.6411	-1.4101	0.3873	0.3763	0.0000
203382_s_at	APOE	apolipoprotein E	NM_000041	-2.3152	-0.9206	0.3976	0.5283	0.1081
203386_at	TBC1D4	TBC1 domain family, member 4	NM_014832	-6.3912	-1.1808	0.1848	0.4411	0.0000
203394_s_at	HES1	hairy and enhancer of split 1 (Drosophila)	NM_005524	-2.4891	-0.8430	0.3387	0.5575	0.1081
203401_at	PRPS2	phosphoribosyl pyrophosphate synthetase 2	NM_001039091 /// NM_002765	-29.5059	-6.8528	0.2323	0.0087	0.0000
203414_at	MMD	monocyte to macrophage differentiation-associated	NM_012329	-5.1565	-1.0139	0.1966	0.4952	0.0000
203458_at	SPR	sepiapterin reductase (7,8-dihydrobiopterin:NADP+ oxidoreductase)	NM_003124	-6.4332	-0.9309	0.1447	0.5245	0.0000
203508_at	TNFRSF1B	tumor necrosis factor receptor superfamily, member 1B	NM_001066	-4.9570	-1.2906	0.2604	0.4088	0.0000
203557_s_at	PCBD1	pterin-4 alpha-carbinolamine dehydratase/dimerization cofactor of hepatocyte nuclear factor 1 alpha	NM_000281	-5.7984	-0.8981	0.1549	0.5366	0.0000
203561_at	FCGR2A	Fc fragment of IgG, low affinity IIa, receptor (CD32)	NM_001136219 /// NM_021642	-4.9946	-1.2074	0.2417	0.4331	0.0000
203569_s_at	OFD1	oral-facial-digital syndrome 1	NM_003611	-6.3037	-1.1356	0.1802	0.4551	0.0000
203583_at	UNC50	unc-50 homolog (C. elegans)	NM_014044	-4.0815	-0.8115	0.1988	0.5698	0.0000

Table S2. Continued

Probe Set ID	Gene Symbol	Gene Title	RefSeq Transcript ID	Score(d)	Numerat-or(r)	Denominator(s+s0)	Fold Change	q-value(%)
Down reglulated								
203608_at	ALDH5A1	aldehyde dehydrogenase 5 family, member A1	NM_001080 /// NM_170740	-37.4356	-6.1253	0.1636	0.0143	0.0000
203620_s_at	FCHSD2	FCH and double SH3 domains 2	NM_014824	-4.8346	-1.2987	0.2686	0.4065	0.0000
203637_s_at	MID1	midline 1 (Opitz/BBB syndrome)	NM_000381 /// NM_001098624 /// NM_001193277 /// NM_001193278 /// NM_001193279 /// NM_001193280 /// NM_001193281 /// NM_033289 /// NM_033290	-3.1962	-0.9620	0.3010	0.5134	0.0622
203685_at	BCL2	B-cell CLL/lymphoma 2	NM_000633 /// NM_000657	-6.4146	-1.2786	0.1993	0.4122	0.0000
203745_at	HCCS	holocytochrome c synthase	NM_001122608 /// NM_001171991 /// NM_005333	-3.4415	-1.0608	0.3082	0.4794	0.0000
203746_s_at	HCCS	holocytochrome c synthase	NM_001122608 /// NM_001171991 /// NM_005333	-4.9418	-0.9212	0.1864	0.5281	0.0000
203767_s_at	STS	steroid sulfatase (microsomal), isozyme s	NM_000351	-2.5074	-1.4550	0.5803	0.3647	0.1081
203841_x_at	MAPRE3	microtubule-associated protein, RP/EB family, member 3	NM_012326	-4.5955	-1.1635	0.2532	0.4464	0.0000

Table S2. Continued

Probe Set ID	Gene Symbol	Gene Title	RefSeq Transcript ID	Score(d)	Numerat-or(r)	Denominator(s+s0)	Fold Change	q-value(%)
Down reglulated								
203911_at	RAP1GAP	RAP1 GTPase activating protein	NM_001145657 /// NM_001145658 /// NM_002885	-3.5449	-0.8435	0.2380	0.5573	0.0000
203921_at	CHST2	carbohydrate (N-acetylglucosamine-6-O) sulfotransferase 2	NM_004267	-7.5269	-1.9058	0.2532	0.2669	0.0000
204012_s_at	LCMT2	leucine carboxyl methyltransferase 2	NM_014793	-6.0715	-0.9671	0.1593	0.5115	0.0000
204019_s_at	SH3YL1	SH3 domain containing, Ysc84-like 1 (S. cerevisiae)	NM_001159597 /// NM_015677	-3.0144	-0.9166	0.3041	0.5297	0.0622
204060_s_at	PRKX /// PRKY	protein kinase, X-linked /// protein kinase, Y-linked	NM_005044 /// NR_028062	-4.5976	-1.2422	0.2702	0.4227	0.0000
204061_at	PRKX	protein kinase, X-linked	NM_005044	-4.7614	-1.2956	0.2721	0.4074	0.0000
204068_at	STK3	serine/threonine kinase 3	NM_006281	-6.1203	-0.9053	0.1479	0.5339	0.0000
204149_s_at	GSTM4	glutathione S-transferase mu 4	NM_000850 /// NM_147148 /// NR_024538	-5.0242	-0.9216	0.1834	0.5279	0.0000
204192_at	CD37	CD37 molecule	NM_001040031 /// NM_001774	-6.4110	-2.0273	0.3162	0.2453	0.0000
204220_at	GMFG	glia maturation factor, gamma	NM_004877	-6.4268	-1.4641	0.2278	0.3625	0.0000
204230_s_at	SLC17A7	solute carrier family 17 (sodium-dependent inorganic phosphate cotransporter), member 7	NM_020309	-2.4541	-0.9221	0.3757	0.5278	0.1081

Table S2. Continued

Probe Set ID	Gene Symbol	Gene Title	RefSeq Transcript ID	Score(d)	Numerat-or(r)	Denominator(s+s0)	Fold Change	q-value(%)
Down reglulated								
204279_at	PSMB9	proteasome (prosome, macropain) subunit, beta type, 9 (large multifunctional peptidase 2)	NM_002800	-3.9408	-0.9363	0.2376	0.5226	0.0000
204373_s_at	CEP350	centrosomal protein 350kDa	NM_014810	-3.2117	-0.8168	0.2543	0.5677	0.0622
204446_s_at	ALOX5	arachidonate 5-lipoxygenase	NM_000698	-2.9086	-1.1218	0.3857	0.4595	0.0622
204466_s_at	SNCA	synuclein, alpha (non A4 component of amyloid precursor)	NM_000345 /// NM_001146054 /// NM_001146055 /// NM_007308	-12.6593	-2.1793	0.1721	0.2208	0.0000
204467_s_at	SNCA	synuclein, alpha (non A4 component of amyloid precursor)	NM_000345 /// NM_001146054 /// NM_001146055 /// NM_007308	-5.1692	-1.3587	0.2628	0.3899	0.0000
204492_at	ARHGAP11A	Rho GTPase activating protein 11A	NM_014783 /// NM_199357	-5.1061	-0.8349	0.1635	0.5606	0.0000
204639_at	ADA	adenosine deaminase	NM_000022	-3.0997	-0.8377	0.2703	0.5595	0.0622
204661_at	CD52	CD52 molecule	NM_001803	-6.4771	-1.6851	0.2602	0.3110	0.0000

Table S2. Continued

Probe Set ID	Gene Symbol	Gene Title	RefSeq Transcript ID	Score(d)	Numerat-or(r)	Denominat or(s+s0)	Fold Change	q-value(%)
Down reglulated								
204820_s_at	BTN3A2 /// BTN3A3	butyrophilin, subfamily 3, member A2 /// butyrophilin, subfamily 3, member A3	NM_001197246 /// NM_001197247 /// NM_001197248 /// NM_001197249 /// NM_006994 /// NM_007047 /// NM_197974	-3.9568	-2.0496	0.5180	0.2416	0.0000
204835_at	POLA1	polymerase (DNA directed), alpha 1, catalytic subunit	NM_016937	-5.6326	-0.9736	0.1729	0.5092	0.0000
204912_at	IL10RA	interleukin 10 receptor, alpha	NM_001558 /// NR_026691	-5.5107	-2.7892	0.5061	0.1447	0.0000
204920_at	CPS1	carbamoyl-phosphate synthase 1, mitochondrial	NM_001122633 /// NM_001122634 /// NM_001875	-4.8770	-1.8763	0.3847	0.2724	0.0000
204976_s_at	AMMECR1	Alport syndrome, mental retardation, midface hypoplasia and elliptocytosis chromosomal region gene 1	NM_001025580 /// NM_001171689 /// NM_015365	-2.7026	-0.8226	0.3044	0.5654	0.0852
204995_at	CDK5R1	cyclin-dependent kinase 5, regulatory subunit 1 (p35)	NM_003885	-2.5285	-0.9160	0.3623	0.5300	0.1081
205024_s_at	RAD51	RAD51 homolog (RecA homolog, E. coli) (S. cerevisiae)	NM_001164269 /// NM_001164270 /// NM_002875 /// NM_133487	-5.0274	-0.8554	0.1701	0.5527	0.0000

Table S2. Continued

Probe Set ID	Gene Symbol	Gene Title	RefSeq Transcript ID	Score(d)	Numerat-or(r)	Denominator(s+s0)	Fold Change	q-value(%)
Down reglulated								
205034_at	CCNE2	cyclin E2	NM_057749	-3.0102	-0.9294	0.3087	0.5251	0.0622
205070_at	ING3	inhibitor of growth family, member 3	NM_019071 /// NM_198267	-3.2390	-0.8482	0.2619	0.5555	0.0000
205074_at	SLC22A5	solute carrier family 22 (organic cation/carnitine transporter), member 5	NM_003060	-3.3823	-1.0658	0.3151	0.4777	0.0000
205110_s_at	FGF13	fibroblast growth factor 13	NM_001139498 /// NM_001139500 /// NM_001139501 /// NM_001139502 /// NM_004114 /// NM_033642	-12.3321	-2.6863	0.2178	0.1554	0.0000
205281_s_at	PIGA	phosphatidylinositol glycan anchor biosynthesis, class A	NM_002641 /// NM_020473 /// NR_033835 /// NR_033836	-5.4601	-1.0096	0.1849	0.4967	0.0000
205285_s_at	FYB	FYN binding protein	NM_001465 /// NM_199335	-7.8322	-1.6957	0.2165	0.3087	0.0000
205541_s_at	GSPT2	G1 to S phase transition 2	NM_018094	-14.0261	-3.7714	0.2689	0.0732	0.0000
205542_at	STEAP1	six transmembrane epithelial antigen of the prostate 1	NM_012449	-2.8263	-1.9385	0.6859	0.2609	0.0622
205564_at	PAGE4	P antigen family, member 4 (prostate associated)	NM_007003	-4.3399	-0.9383	0.2162	0.5218	0.0000

Table S2. Continued

Probe Set ID	Gene Symbol	Gene Title	RefSeq Transcript ID	Score(d)	Numerat-or(r)	Denominat or(s+s0)	Fold Change	q-value(%)
Down reglulated								
205590_at	RASGRP1	RAS guanyl releasing protein 1 (calcium and DAG-regulated)	NM_001128602 /// NM_005739	-3.7470	-1.1471	0.3061	0.4515	0.0000
205771_s_at	AKAP7	A kinase (PRKA) anchor protein 7	NM_004842 /// NM_016377 /// NM_138633	-4.1023	-1.7106	0.4170	0.3055	0.0000
205830_at	CLGN	calmegin	NM_001130675 /// NM_004362	-6.9351	-1.7323	0.2498	0.3010	0.0000
205896_at	SLC22A4	solute carrier family 22 (organic cation/ergothioneine transporter), member 4	NM_003059	-3.5569	-1.5342	0.4313	0.3453	0.0000
205932_s_at	MSX1	msh homeobox 1	NM_002448	-10.2233	-2.2949	0.2245	0.2038	0.0000
205938_at	PPM1E	protein phosphatase, Mg2+/Mn2+ dependent, 1E	NM_014906	-2.7164	-1.7700	0.6516	0.2932	0.0852
205950_s_at	CA1	carbonic anhydrase I	NM_001128829 /// NM_001128830 /// NM_001128831 /// NM_001164830 /// NM_001738	-2.2852	-1.3219	0.5785	0.4000	0.1081
206023_at	NMU	neuromedin U	NM_006681	-3.5688	-1.1610	0.3253	0.4472	0.0000
206036_s_at	REL	v-rel reticuloendotheliosis viral oncogene homolog (avian)	NM_002908	-5.1752	-1.2390	0.2394	0.4237	0.0000
206059_at	ZNF91	zinc finger protein 91	NM_003430	-11.5537	-2.1727	0.1881	0.2218	0.0000

Table S2. Continued

Probe Set ID	Gene Symbol	Gene Title	RefSeq Transcript ID	Score(d)	Numerat-or(r)	Denominator(s+s0)	Fold Change	q-value(%)
Down reglulated								
206103_at	RAC3	ras-related C3 botulinum toxin substrate 3 (rho family, small GTP binding protein Rac3)	NM_005052	-4.5373	-0.9054	0.1996	0.5339	0.0000
206116_s_at	TPM1	tropomyosin 1 (alpha)	NM_000366 /// NM_001018004 /// NM_001018005 /// NM_001018006 /// NM_001018007 /// NM_001018008 /// NM_001018020	-7.0009	-1.2276	0.1753	0.4270	0.0000
206120_at	CD33	CD33 molecule	NM_001082618 /// NM_001177608 /// NM_001772	-6.3353	-1.2069	0.1905	0.4332	0.0000
206147_x_at	SCML2	sex comb on midleg-like 2 (Drosophila)	NM_006089 /// NR_033717	-8.9998	-2.2816	0.2535	0.2057	0.0000
206218_at	MAGEB2	melanoma antigen family B, 2	NM_002364	-14.2924	-2.3578	0.1650	0.1951	0.0000
206219_s_at	VAV1	vav 1 guanine nucleotide exchange factor	NM_005428	-4.3000	-1.2288	0.2858	0.4267	0.0000
206295_at	IL18	interleukin 18 (interferon-gamma-inducing factor)	NM_001562	-4.3356	-1.2663	0.2921	0.4157	0.0000
206373_at	ZIC1	Zic family member 1 (odd-paired homolog, Drosophila)	NM_003412	-7.9683	-2.3250	0.2918	0.1996	0.0000
206385_s_at	ANK3	ankyrin 3, node of Ranvier (ankyrin G)	NM_001149 /// NM_020987	-7.7065	-2.0118	0.2610	0.2480	0.0000

Table S2. Continued

Probe Set ID	Gene Symbol	Gene Title	RefSeq Transcript ID	Score(d)	Numerat-or(r)	Denominator(s+s0)	Fold Change	q-value(%)
Down reglulated								
206412_at	FER	fer (fps/fes related) tyrosine kinase	NM_005246	-6.2766	-1.7609	0.2806	0.2951	0.0000
206414_s_at	ASAP2	ArfGAP with SH3 domain, ankyrin repeat and PH domain 2	NM_001135191 /// NM_003887	-7.0745	-2.3363	0.3302	0.1980	0.0000
206424_at	CYP26A1	cytochrome P450, family 26, subfamily A, polypeptide 1	NM_000783 /// NM_057157	-6.5238	-2.5041	0.3838	0.1763	0.0000
206464_at	BMX	BMX non-receptor tyrosine kinase	NM_001721 /// NM_203281	-5.9761	-2.3436	0.3922	0.1970	0.0000
206499_s_at	RCC1	regulator of chromosome condensation 1	NM_001048194 /// NM_001048195 /// NM_001048199 /// NM_001269 /// NR_030725 /// NR_030726	-4.8285	-0.8700	0.1802	0.5471	0.0000
206554_x_at	SETMAR	SET domain and mariner transposase fusion gene	NM_006515 /// NR_024022	-5.2001	-0.9349	0.1798	0.5231	0.0000
206609_at	MAGEC1	melanoma antigen family C, 1	NM_005462	-3.6312	-0.9270	0.2553	0.5259	0.0000
206626_x_at	SSX1	synovial sarcoma, X breakpoint 1	NM_005635	-34.5526	-5.8366	0.1689	0.0175	0.0000
206627_s_at	SSX1	synovial sarcoma, X breakpoint 1	NM_005635	-19.4768	-5.2828	0.2712	0.0257	0.0000
206761_at	CD96	CD96 molecule	NM_005816 /// NM_198196	-3.3901	-1.7192	0.5071	0.3037	0.0000
206793_at	PNMT	phenylethanolamine N-methyltransferase	NM_002686	-3.9135	-1.0108	0.2583	0.4963	0.0000
206834_at	HBD	hemoglobin, delta	NM_000519	-2.5060	-1.0409	0.4154	0.4860	0.1081

Table S2. Continued

Probe Set ID	Gene Symbol	Gene Title	RefSeq Transcript ID	Score(d)	Numerat-or(r)	Denominat or(s+s0)	Fold Change	q-value(%)
Down reglulated								
206892_at	AMHR2	anti-Mullerian hormone receptor, type II	NM_001164690 /// NM_001164691 /// NM_020547	-4.8270	-0.9996	0.2071	0.5001	0.0000
206897_at	PAGE1	P antigen family, member 1 (prostate associated)	NM_003785	-28.2358	-5.5588	0.1969	0.0212	0.0000
206905_s_at	MATN1	matrilin 1, cartilage matrix protein	NM_002379	-4.4152	-1.3126	0.2973	0.4026	0.0000
206915_at	NKX2-2	NK2 homeobox 2	NM_002509	-10.2911	-1.9296	0.1875	0.2625	0.0000
207002_s_at	PLAGL1	pleiomorphic adenoma gene-like 1	NM_001080951 /// NM_001080952 /// NM_001080953 /// NM_001080954 /// NM_001080955 /// NM_001080956 /// NM_002656 /// NM_006718	-17.9622	-4.8925	0.2724	0.0337	0.0000
207169_x_at	DDR1	discoidin domain receptor tyrosine kinase 1	NM_001954 /// NM_013993 /// NM_013994	-6.0746	-0.8911	0.1467	0.5392	0.0000

Table S2. Continued

Probe Set ID	Gene Symbol	Gene Title	RefSeq Transcript ID	Score(d)	Numerat-or(r)	Denominat or(s+s0)	Fold Change	q-value(%)
Down reglulated								
207247_s_at	ZFX /// ZFY	zinc finger protein, X-linked /// zinc finger protein, Y-linked	NM_001145275 /// NM_001145276 /// NM_001178084 /// NM_001178085 /// NM_001178086 /// NM_001178095 /// NM_003410 /// NM_003411	-4.2777	-1.0284	0.2404	0.4903	0.0000
207281_x_at	VCX2	variable charge, X-linked 2	NM_016378	-6.6170	-1.7833	0.2695	0.2905	0.0000
207387_s_at	GK	glycerol kinase	NM_000167 /// NM_001128127 /// NM_203391	-17.5529	-2.8920	0.1648	0.1347	0.0000
207493_x_at	SSX2 /// SSX2B	synovial sarcoma, X breakpoint 2 /// synovial sarcoma, X breakpoint 2B	NM_001164417 /// NM_003147 /// NM_175698	-9.1569	-2.8862	0.3152	0.1353	0.0000
207524_at	ST7	suppression of tumorigenicity 7	NM_018412 /// NM_021908	-3.3105	-0.8257	0.2494	0.5642	0.0000
207534_at	MAGEB1	melanoma antigen family B, 1	NM_002363 /// NM_177404 /// NM_177415	-29.6072	-5.7712	0.1949	0.0183	0.0000

Table S2. Continued

Probe Set ID	Gene Symbol	Gene Title	RefSeq Transcript ID	Score(d)	Numerat-or(r)	Denominator(s+s0)	Fold Change	q-value(%)
Down reglulated								
207542_s_at	AQP1	aquaporin 1 (Colton blood group)	NM_001185060 /// NM_001185061 /// NM_001185062 /// NM_198098	-4.1368	-1.4964	0.3617	0.3544	0.0000
207551_s_at	MSL3	male-specific lethal 3 homolog (Drosophila)	NM_001193270 /// NM_006800 /// NM_078628 /// NM_078629	-4.1417	-0.8312	0.2007	0.5621	0.0000
207666_x_at	SSX3	synovial sarcoma, X breakpoint 3	NM_021014 /// NM_175711	-8.5053	-2.8842	0.3391	0.1355	0.0000
207717_s_at	PKP2	plakophilin 2	NM_001005242 /// NM_004572	-9.4220	-2.6666	0.2830	0.1575	0.0000
207732_s_at	DLG3	discs, large homolog 3 (Drosophila)	NM_001166278 /// NM_020730 /// NM_021120	-2.9189	-1.2370	0.4238	0.4242	0.0622
207749_s_at	PPP2R3A	protein phosphatase 2, regulatory subunit B", alpha	NM_001190447 /// NM_002718 /// NM_181897	-2.6803	-0.9132	0.3407	0.5310	0.0852
207775_at	MGC4859	hypothetical LOC79150	NA	-6.6520	-0.8947	0.1345	0.5379	0.0000
207843_x_at	CYB5A	cytochrome b5 type A (microsomal)	NM_001190807 /// NM_001914 /// NM_148923	-24.1832	-3.7351	0.1544	0.0751	0.0000

Table S2. Continued

Probe Set ID	Gene Symbol	Gene Title	RefSeq Transcript ID	Score(d)	Numerat-or(r)	Denominator(s+s0)	Fold Change	q-value(%)
Down reglulated								
207847_s_at	MUC1	mucin 1, cell surface associated	NM_001018016 /// NM_001018017 /// NM_001044390 /// NM_001044391 /// NM_001044392 /// NM_001044393 /// NM_002456	-3.3219	-0.8099	0.2438	0.5704	0.0000
207858_s_at	PKLR	pyruvate kinase, liver and RBC	NM_000298 /// NM_181871	-2.8300	-0.8607	0.3041	0.5507	0.0622
207883_s_at	TFR2	transferrin receptor 2	NM_003227	-4.0223	-0.8457	0.2102	0.5565	0.0000
207920_x_at	ZFX	zinc finger protein, X-linked	NM_001178084 /// NM_001178085 /// NM_001178086 /// NM_001178095 /// NM_003410	-5.8747	-1.1162	0.1900	0.4613	0.0000
207943_x_at	PLAGL1	pleiomorphic adenoma gene-like 1	NM_001080951 /// NM_001080952 /// NM_001080953 /// NM_001080954 /// NM_001080955 /// NM_001080956 /// NM_002656 /// NM_006718	-18.3110	-3.9190	0.2140	0.0661	0.0000

Table S2. Continued

Probe Set ID	Gene Symbol	Gene Title	RefSeq Transcript ID	Score(d)	Numerat-or(r)	Denominator(s+s0)	Fold Change	q-value(%)
Down reglulated								
208002_s_at	ACOT7	acyl-CoA thioesterase 7	NM_007274 /// NM_181864 /// NM_181865 /// NM_181866	-4.2822	-0.9211	0.2151	0.5281	0.0000
208119_s_at	ZNF93	zinc finger protein 93	NM_031218	-5.2917	-1.3930	0.2632	0.3808	0.0000
208165_s_at	PRSS16	protease, serine, 16 (thymus)	NM_005865	-4.6112	-2.1568	0.4677	0.2242	0.0000
208451_s_at	C4A /// C4B /// LOC100509001	complement component 4A (Rodgers blood group) /// complement component 4B (Chido blood group) /// complement C4-B-like	NM_001002029 /// NM_007293 /// XM_003120291 /// XM_003120292	-3.3475	-0.8074	0.2412	0.5714	0.0000
208650_s_at	CD24	CD24 molecule	NM_013230	-2.5812	-2.4695	0.9567	0.1806	0.1081

Table S2. Continued

Probe Set ID	Gene Symbol	Gene Title	RefSeq Transcript ID	Score(d)	Numerat-or(r)	Denominator(s+s0)	Fold Change	q-value(%)
Down reglulated								
208812_x_at	HLA-C	major histocompatibility complex, class I, C	NM_002117 /// XM_003119244 /// XM_003119245 /// XM_003119246 /// XM_003119247 /// XM_003119248 /// XM_003119249 /// XM_003119250 /// XM_003119251 /// XM_003119252 /// XM_003119253 /// XM_003119254 /// XM_003119255 /// XM_003119256 /// XM_003119257 /// XM_003119359 /// XM_003119360 /// XM_003119361 /// XM_003119362 /// XM_003119363 /// XM_003119364 /// XM_003119365 /// XM_003119366 /// XM_003119367 /// XM_003119368 /// XM_003119369 /// XM_003119370 /// XM_003119371 ///	-6.2268	-1.3108	0.2105	0.4031	0.0000

Table S2. Continued

Probe Set ID	Gene Symbol	Gene Title	RefSeq Transcript ID	Score(d)	Numerat-or(r)	Denominator(s+s0)	Fold Change	q-value(%)
Down reglulated								
208829_at	TAPBP	TAP binding protein (tapasin)	NM_003190 /// NM_172208 /// NM_172209	-4.5580	-1.3041	0.2861	0.4050	0.0000
208885_at	LCP1	lymphocyte cytosolic protein 1 (L-plastin)	NM_002298	-21.5674	-4.0168	0.1862	0.0618	0.0000
208920_at	SRI	sorcin	NM_003130 /// NM_198901	-4.8817	-0.9266	0.1898	0.5261	0.0000
208955_at	DUT	deoxyuridine triphosphatase	NM_001025248 /// NM_001025249 /// NM_001948	-6.4130	-1.0788	0.1682	0.4734	0.0000
209006_s_at	C1orf63	chromosome 1 open reading frame 63	NM_020317	-3.0754	-0.9058	0.2945	0.5337	0.0622
209007_s_at	C1orf63	chromosome 1 open reading frame 63	NM_020317	-5.1776	-0.8784	0.1697	0.5440	0.0000
209014_at	MAGED1	melanoma antigen family D, 1	NM_001005332 /// NM_001005333 /// NM_006986	-12.7135	-3.5189	0.2768	0.0872	0.0000
209043_at	PAPSS1	3'-phosphoadenosine 5'-phosphosulfate synthase 1	NM_005443	-4.4222	-0.8410	0.1902	0.5583	0.0000
209047_at	AQP1	aquaporin 1 (Colton blood group)	NM_001185060 /// NM_001185061 /// NM_001185062 /// NM_198098	-5.6213	-1.9301	0.3434	0.2624	0.0000
209193_at	PIM1	pim-1 oncogene	NM_002648	-3.6501	-1.1762	0.3222	0.4425	0.0000

Table S2. Continued

Probe Set ID	Gene Symbol	Gene Title	RefSeq Transcript ID	Score(d)	Numerat-or(r)	Denominator(s+s0)	Fold Change	q-value(%)
Down reglulated								
209269_s_at	SYK	spleen tyrosine kinase	NM_001135052 /// NM_001174167 /// NM_001174168 /// NM_003177	-5.8880	-0.8717	0.1481	0.5465	0.0000
209289_at	NFIB	nuclear factor I/B	NM_001190737 /// NM_001190738 /// NM_005596	-4.4444	-1.6178	0.3640	0.3258	0.0000
209290_s_at	NFIB	nuclear factor I/B	NM_001190737 /// NM_001190738 /// NM_005596	-5.1514	-1.7899	0.3475	0.2892	0.0000
209318_x_at	PLAGL1	pleiomorphic adenoma gene-like 1	NM_001080951 /// NM_001080952 /// NM_001080953 /// NM_001080954 /// NM_001080955 /// NM_001080956 /// NM_002656 /// NM_006718	-16.8752	-5.0721	0.3006	0.0297	0.0000
209321_s_at	ADCY3	adenylate cyclase 3	NM_004036	-4.7619	-0.8974	0.1885	0.5369	0.0000
209366_x_at	CYB5A	cytochrome b5 type A (microsomal)	NM_001190807 /// NM_001914 /// NM_148923	-18.8015	-3.5392	0.1882	0.0860	0.0000
209369_at	ANXA3	annexin A3	NM_005139	-5.9110	-1.3355	0.2259	0.3963	0.0000

Table S2. Continued

Probe Set ID	Gene Symbol	Gene Title	RefSeq Transcript ID	Score(d)	Numerat-or(r)	Denominat or(s+s0)	Fold Change	q-value(%)
Down reglulated								
209425_at	AMACR	alpha-methylacyl-CoA racemase	NM_001167595 /// NM_001167596 /// NM_001167597 /// NM_001167598 /// NM_014324 /// NM_203382	-4.1293	-0.9555	0.2314	0.5157	0.0000
209431_s_at	PATZ1	POZ (BTB) and AT hook containing zinc finger 1	NM_014323 /// NM_032050 /// NM_032051 /// NM_032052	-4.7218	-0.9363	0.1983	0.5226	0.0000
209438_at	PHKA2	phosphorylase kinase, alpha 2 (liver)	NM_000292	-6.2431	-1.2702	0.2035	0.4146	0.0000
209439_s_at	PHKA2	phosphorylase kinase, alpha 2 (liver)	NM_000292	-5.2885	-1.2466	0.2357	0.4215	0.0000
209545_s_at	RIPK2	receptor-interacting serine-threonine kinase 2	NM_003821	-3.6093	-1.0298	0.2853	0.4898	0.0000
209550_at	NDN	necdin homolog (mouse)	NM_002487	-10.6799	-3.3203	0.3109	0.1001	0.0000
209615_s_at	PAK1	p21 protein (Cdc42/Rac)-activated kinase 1	NM_001128620 /// NM_002576	-2.8188	-0.9121	0.3236	0.5314	0.0622
209688_s_at	CCDC93	coiled-coil domain containing 93	NM_019044	-3.7806	-0.9860	0.2608	0.5049	0.0000
209723_at	SERPINB9	serpin peptidase inhibitor, clade B (ovalbumin), member 9	NM_004155	-3.6724	-1.3968	0.3804	0.3798	0.0000
209734_at	NCKAP1L	NCK-associated protein 1-like	NM_001184976 /// NM_005337	-4.1373	-1.1004	0.2660	0.4664	0.0000

Table S2. Continued

Probe Set ID	Gene Symbol	Gene Title	RefSeq Transcript ID	Score(d)	Numerat-or(r)	Denominat or(s+s0)	Fold Change	q-value(%)
Down reglulated								
209735_at	ABCG2	ATP-binding cassette, sub-family G (WHITE), member 2	NM_004827	-5.2178	-1.0989	0.2106	0.4669	0.0000
209737_at	MAGI2	membrane associated guanylate kinase, WW and PDZ domain containing 2	NM_012301	-2.8824	-1.1646	0.4040	0.4461	0.0622
209739_s_at	PNPLA4	patatin-like phospholipase domain containing 4	NM_001142389 /// NM_001172672 /// NM_004650	-5.6001	-1.1932	0.2131	0.4373	0.0000
209740_s_at	PNPLA4	patatin-like phospholipase domain containing 4	NM_001142389 /// NM_001172672 /// NM_004650	-2.4528	-0.8302	0.3385	0.5624	0.1081
209771_x_at	CD24	CD24 molecule	NM_013230	-2.3626	-1.8796	0.7955	0.2718	0.1081
209846_s_at	BTN3A2	butyrophilin, subfamily 3, member A2	NM_001197246 /// NM_001197247 /// NM_001197248 /// NM_001197249 /// NM_007047	-5.6273	-2.0553	0.3652	0.2406	0.0000
209871_s_at	APBA2	amyloid beta (A4) precursor protein-binding, family A, member 2	NM_001130414 /// NM_005503	-2.5985	-0.8472	0.3260	0.5559	0.1081
209872_s_at	PKP3	plakophilin 3	NM_007183	-5.2503	-1.1680	0.2225	0.4450	0.0000
209873_s_at	PKP3	plakophilin 3	NM_007183	-5.5407	-1.0056	0.1815	0.4981	0.0000
209879_at	SELPLG	selectin P ligand	NM_003006	-3.9900	-0.9248	0.2318	0.5267	0.0000

Table S2. Continued

Probe Set ID	Gene Symbol	Gene Title	RefSeq Transcript ID	Score(d)	Numerat-or(r)	Denominator(s+s0)	Fold Change	q-value(%)
Down reglulated								
209898_x_at	ITSN2	intersectin 2	NM_006277 /// NM_019595 /// NM_147152	-4.0061	-0.8751	0.2184	0.5452	0.0000
209901_x_at	AIF1	allograft inflammatory factor 1	NM_001623 /// NM_004847 /// NM_032955	-2.3331	-0.9439	0.4046	0.5198	0.1081
209907_s_at	ITSN2	intersectin 2	NM_006277 /// NM_019595 /// NM_147152	-6.0355	-1.1058	0.1832	0.4646	0.0000
210022_at	PCGF1	polycomb group ring finger 1	NM_032673	-4.0207	-0.9306	0.2315	0.5246	0.0000
210023_s_at	PCGF1	polycomb group ring finger 1	NM_032673	-5.3515	-1.4443	0.2699	0.3675	0.0000
210026_s_at	CARD10	caspase recruitment domain family, member 10	NM_014550	-4.9604	-1.6674	0.3361	0.3148	0.0000
210117_at	SPAG1	sperm associated antigen 1	NM_003114 /// NM_172218	-3.5134	-1.2402	0.3530	0.4233	0.0000
210232_at	CDC42	cell division cycle 42 (GTP binding protein, 25kDa)	NM_001039802 /// NM_001791 /// NM_044472	-3.0280	-0.8108	0.2678	0.5701	0.0622
210296_s_at	PEX2	peroxisomal biogenesis factor 2	NM_000318 /// NM_001079867 /// NM_001172086 /// NM_001172087	-3.9866	-0.8824	0.2213	0.5425	0.0000

Table S2. Continued

Probe Set ID	Gene Symbol	Gene Title	RefSeq Transcript ID	Score(d)	Numerat-or(r)	Denominat or(s+s0)	Fold Change	q-value(%)
Down reglulated								
210375_at	PTGER3	prostaglandin E receptor 3 (subtype EP3)	NM_001126044 /// NM_198714 /// NM_198715 /// NM_198716 /// NM_198717 /// NM_198718 /// NM_198719 /// NR_028292 /// NR_028293 /// NR_028294	-4.4130	-0.9830	0.2227	0.5059	0.0000
210394_x_at	SSX4 /// SSX4B	synovial sarcoma, X breakpoint 4 /// synovial sarcoma, X breakpoint 4B	NM_001034832 /// NM_001040612 /// NM_005636 /// NM_175729	-18.1540	-4.7838	0.2635	0.0363	0.0000
210437_at	MAGEA9 /// MAGEA9B	melanoma antigen family A, 9 /// melanoma antigen family A, 9B	NM_001080790 /// NM_005365	-19.3312	-3.0784	0.1592	0.1184	0.0000
210495_x_at	FN1	fibronectin 1	NM_002026 /// NM_054034 /// NM_212474 /// NM_212476 /// NM_212478 /// NM_212482	-3.7245	-1.1702	0.3142	0.4444	0.0000

Table S2. Continued

Probe Set ID	Gene Symbol	Gene Title	RefSeq Transcript ID	Score(d)	Numerat-or(r)	Denominat or(s+s0)	Fold Change	q-value(%)
Down reglulated								
210497_x_at	SSX2 /// SSX2B	synovial sarcoma, X breakpoint 2 /// synovial sarcoma, X breakpoint 2B	NM_001164417 /// NM_003147 /// NM_175698	-13.8566	-4.1008	0.2959	0.0583	0.0000
210529_s_at	FAM115A	family with sequence similarity 115, member A	NM_014719	-7.0521	-0.9785	0.1387	0.5075	0.0000
210613_s_at	SYNGR1	synaptogyrin 1	NM_004711 /// NM_145731 /// NM_145738	-4.4911	-1.0844	0.2414	0.4716	0.0000
210620_s_at	GTF3C2	general transcription factor IIIC, polypeptide 2, beta 110kDa	NM_001035521 /// NM_001521	-5.2164	-1.0488	0.2011	0.4834	0.0000
210624_s_at	ILVBL	ilvB (bacterial acetolactate synthase)-like	NM_006844	-3.5300	-0.9320	0.2640	0.5241	0.0000
210627_s_at	MOGS	mannosyl-oligosaccharide glucosidase	NM_001146158 /// NM_006302	-3.9319	-0.9280	0.2360	0.5256	0.0000
210635_s_at	KLHL20	kelch-like 20 (Drosophila)	NM_014458	-5.1299	-0.8384	0.1634	0.5593	0.0000
210662_at	KYNU	kynureninase (L-kynurenine hydrolase)	NM_001032998 /// NM_003937	-3.7099	-1.3812	0.3723	0.3839	0.0000

Table S2. Continued

Probe Set ID	Gene Symbol	Gene Title	RefSeq Transcript ID	Score(d)	Numerat-or(r)	Denominator(s+s0)	Fold Change	q-value(%)
Down reglulated								
210694_s_at	MID1	midline 1 (Opitz/BBB syndrome)	NM_000381 /// NM_001098624 /// NM_001193277 /// NM_001193278 /// NM_001193279 /// NM_001193280 /// NM_001193281 /// NM_033289 /// NM_033290	-4.6332	-0.9446	0.2039	0.5196	0.0000
210749_x_at	DDR1	discoidin domain receptor tyrosine kinase 1	NM_001954 /// NM_013993 /// NM_013994	-4.8565	-0.8109	0.1670	0.5700	0.0000
210757_x_at	DAB2	disabled homolog 2, mitogen-responsive phosphoprotein (Drosophila)	NM_001343	-3.8168	-0.9533	0.2498	0.5165	0.0000
210830_s_at	PON2	paraoxonase 2	NM_000305 /// NM_001018161	-15.1819	-4.5559	0.3001	0.0425	0.0000

Table S2. Continued

Probe Set ID	Gene Symbol	Gene Title	RefSeq Transcript ID	Score(d)	Numerat-or(r)	Denominator(s+s0)	Fold Change	q-value(%)
Down reglulated								
210831_s_at	PTGER3	prostaglandin E receptor 3 (subtype EP3)	NM_001126044 /// NM_198714 /// NM_198715 /// NM_198716 /// NM_198717 /// NM_198718 /// NM_198719 /// NR_028292 /// NR_028293 /// NR_028294	-3.2123	-0.8758	0.2727	0.5449	0.0622
210833_at	PTGER3	prostaglandin E receptor 3 (subtype EP3)	NM_001126044 /// NM_198714 /// NM_198715 /// NM_198716 /// NM_198717 /// NM_198718 /// NM_198719 /// NR_028292 /// NR_028293 /// NR_028294	-7.1822	-1.8192	0.2533	0.2834	0.0000
210870_s_at	EPM2A	epilepsy, progressive myoclonus type 2A, Lafora disease (laforin)	NM_001018041 /// NM_005670	-3.1263	-0.8953	0.2864	0.5377	0.0622

Table S2. Continued

Probe Set ID	Gene Symbol	Gene Title	RefSeq Transcript ID	Score(d)	Numerat-or(r)	Denominat or(s+s0)	Fold Change	q-value(%)
Down reglulated								
210963_s_at	GYG2	glycogenin 2	NM_001079855 /// NM_001184702 /// NM_001184703 /// NM_001184704 /// NM_003918	-3.2757	-1.0324	0.3152	0.4889	0.0000
210964_s_at	GYG2	glycogenin 2	NM_001079855 /// NM_001184702 /// NM_001184703 /// NM_001184704 /// NM_003918	-7.2394	-1.5412	0.2129	0.3436	0.0000
210986_s_at	TPM1	tropomyosin 1 (alpha)	NM_000366 /// NM_001018004 /// NM_001018005 /// NM_001018006 /// NM_001018007 /// NM_001018008 /// NM_001018020	-10.0121	-1.2630	0.1261	0.4167	0.0000

Table S2. Continued

Probe Set ID	Gene Symbol	Gene Title	RefSeq Transcript ID	Score(d)	Numerat-or(r)	Denominat or(s+s0)	Fold Change	q-value(%)
Down reglulated								
210987_x_at	TPM1	tropomyosin 1 (alpha)	NM_000366 /// NM_001018004 /// NM_001018005 /// NM_001018006 /// NM_001018007 /// NM_001018008 /// NM_001018020	-7.1063	-1.2652	0.1780	0.4160	0.0000
210992_x_at	FCGR2C	Fc fragment of IgG, low affinity IIc, receptor for (CD32) (gene/pseudogene)	NM_201563	-2.4884	-0.8887	0.3571	0.5401	0.1081
211003_x_at	TGM2	transglutaminase 2 (C polypeptide, protein-glutamine-gamma-glutamyltransferase)	NM_004613 /// NM_198951	-3.0805	-0.9087	0.2950	0.5327	0.0622
211078_s_at	STK3	serine/threonine kinase 3	NM_006281	-2.3842	-0.8195	0.3437	0.5666	0.1081
211207_s_at	ACSL6	acyl-CoA synthetase long-chain family member 6	NM_001009185 /// NM_015256	-2.7931	-0.8503	0.3044	0.5547	0.0852
211214_s_at	DAPK1	death-associated protein kinase 1	NM_004938	-2.3712	-0.8305	0.3503	0.5623	0.1081
211337_s_at	TUBGCP4	tubulin, gamma complex associated protein 4	NM_014444	-4.1702	-0.9134	0.2190	0.5309	0.0000
211395_x_at	FCGR2C	Fc fragment of IgG, low affinity IIc, receptor for (CD32) (gene/pseudogene)	NM_201563	-2.7281	-0.9427	0.3455	0.5203	0.0852
211403_x_at	VCX2	variable charge, X-linked 2	NM_016378	-4.2580	-1.3297	0.3123	0.3979	0.0000

Table S2. Continued

Probe Set ID	Gene Symbol	Gene Title	RefSeq Transcript ID	Score(d)	Numerat-or(r)	Denominator(s+s0)	Fold Change	q-value(%)
Down reglulated								
211425_x_at	SSX4 /// SSX4B	synovial sarcoma, X breakpoint 4 /// synovial sarcoma, X breakpoint 4B	NM_001034832 /// NM_001040612 /// NM_005636 /// NM_175729	-7.3696	-3.8556	0.5232	0.0691	0.0000
211434_s_at	CCRL2	chemokine (C-C motif) receptor-like 2	NM_001130910 /// NM_003965	-4.5266	-1.1933	0.2636	0.4373	0.0000
211560_s_at	ALAS2	aminolevulinate, delta-, synthase 2	NM_000032 /// NM_001037967 /// NM_001037968	-2.5465	-0.9553	0.3751	0.5158	0.1081
211670_x_at	SSX3	synovial sarcoma, X breakpoint 3	NM_021014 /// NM_175711	-7.1956	-2.1357	0.2968	0.2276	0.0000
211719_x_at	FN1	fibronectin 1	NM_002026 /// NM_054034 /// NM_212474 /// NM_212476 /// NM_212478 /// NM_212482	-6.0436	-1.9526	0.3231	0.2584	0.0000
211731_x_at	SSX3	synovial sarcoma, X breakpoint 3	NM_021014 /// NM_175711	-8.9990	-2.2130	0.2459	0.2157	0.0000
211794_at	FYB	FYN binding protein	NM_001465 /// NM_199335	-10.6577	-2.5687	0.2410	0.1686	0.0000
211795_s_at	FYB	FYN binding protein	NM_001465 /// NM_199335	-8.1302	-3.3249	0.4090	0.0998	0.0000

Table S2. Continued

Probe Set ID	Gene Symbol	Gene Title	RefSeq Transcript ID	Score(d)	Numerat-or(r)	Denominator(s+s0)	Fold Change	q-value(%)
Down reglulated								
211796_s_at	TRBC1 /// TRBC2	T cell receptor beta constant 1 /// T cell receptor beta constant 2	NA	-2.3966	-1.5883	0.6627	0.3326	0.1081
211814_s_at	CCNE2	cyclin E2	NM_057749	-3.7452	-1.2037	0.3214	0.4342	0.0000
212085_at	SLC25A6	solute carrier family 25 (mitochondrial carrier; adenine nucleotide translocator), member 6	NM_001636	-5.9441	-0.9583	0.1612	0.5147	0.0000
212097_at	CAV1	caveolin 1, caveolae protein, 22kDa	NM_001172895 /// NM_001172896 /// NM_001172897 /// NM_001753	-7.2961	-2.6869	0.3683	0.1553	0.0000
212202_s_at	TMEM87A	transmembrane protein 87A	NM_001110503 /// NM_015497	-4.6443	-0.9528	0.2051	0.5166	0.0000
212203_x_at	IFITM3	interferon induced transmembrane protein 3 (1-8U)	NM_021034	-5.7241	-1.0673	0.1865	0.4772	0.0000
212204_at	TMEM87A	transmembrane protein 87A	NM_001110503 /// NM_015497	-4.3300	-0.8750	0.2021	0.5453	0.0000
212274_at	LPIN1	lipin 1	NM_145693	-3.9216	-0.8450	0.2155	0.5567	0.0000
212458_at	SPRED2	sprouty-related, EVH1 domain containing 2	NM_001128210 /// NM_181784	-3.6993	-0.8210	0.2219	0.5660	0.0000

Table S2. Continued

Probe Set ID	Gene Symbol	Gene Title	RefSeq Transcript ID	Score(d)	Numerat-or(r)	Denominator(s+s0)	Fold Change	q-value(%)
Down reglulated								
212464_s_at	FN1	fibronectin 1	NM_002026 /// NM_054034 /// NM_212474 /// NM_212476 /// NM_212478 /// NM_212482	-5.0101	-1.6454	0.3284	0.3196	0.0000
212605_s_at	NA	NA	NA	-3.6433	-0.8322	0.2284	0.5617	0.0000
212729_at	DLG3	discs, large homolog 3 (Drosophila)	NM_001166278 /// NM_020730 /// NM_021120	-3.7534	-0.9882	0.2633	0.5041	0.0000
212737_at	GM2A	GM2 ganglioside activator	NM_000405 /// NM_001167607	-5.7807	-1.1711	0.2026	0.4441	0.0000
212739_s_at	NME4	non-metastatic cells 4, protein expressed in	NM_005009	-3.8988	-0.8441	0.2165	0.5571	0.0000
212741_at	MAOA	monoamine oxidase A	NM_000240	-6.4169	-1.1895	0.1854	0.4385	0.0000
212775_at	OBSL1	obscurin-like 1	NM_001173408 /// NM_001173431 /// NM_015311	-2.9906	-1.0712	0.3582	0.4759	0.0622
212776_s_at	OBSL1	obscurin-like 1	NM_001173408 /// NM_001173431 /// NM_015311	-3.1289	-0.8680	0.2774	0.5479	0.0622
212796_s_at	TBC1D2B	TBC1 domain family, member 2B	NM_015079 /// NM_144572	-4.1481	-0.9064	0.2185	0.5335	0.0000

Table S2. Continued

Probe Set ID	Gene Symbol	Gene Title	RefSeq Transcript ID	Score(d)	Numerat-or(r)	Denominat or(s+s0)	Fold Change	q-value(%)
Down reglulated								
212826_s_at	SLC25A6	solute carrier family 25 (mitochondrial carrier; adenine nucleotide translocator), member 6	NM_001636	-5.0364	-0.8268	0.1642	0.5638	0.0000
212841_s_at	PPFIBP2	PTPRF interacting protein, binding protein 2 (liprin beta 2)	NM_003621	-7.1173	-1.7552	0.2466	0.2962	0.0000
212854_x_at	NBPF10	neuroblastoma breakpoint family, member 10	NM_001039703	-3.2838	-1.0557	0.3215	0.4811	0.0000
212886_at	CCDC69	coiled-coil domain containing 69	NM_015621	-5.2497	-1.7575	0.3348	0.2958	0.0000
212949_at	NCAPH	non-SMC condensin I complex, subunit H	NM_015341	-3.7890	-0.8604	0.2271	0.5508	0.0000
212976_at	LRRC8B	leucine rich repeat containing 8 family, member B	NM_001134476 /// NM_015350	-3.2889	-0.8746	0.2659	0.5454	0.0000
212979_s_at	FAM115A	family with sequence similarity 115, member A	NM_014719	-7.3567	-1.2602	0.1713	0.4175	0.0000
213029_at	NFIB	nuclear factor I/B	NM_001190737 /// NM_001190738 /// NM_005596	-2.4891	-1.0687	0.4294	0.4767	0.1081
213032_at	NFIB	nuclear factor I/B	NM_001190737 /// NM_001190738 /// NM_005596	-7.6498	-0.9233	0.1207	0.5273	0.0000
213160_at	DOCK2	dedicator of cytokinesis 2	NM_004946	-12.4248	-2.5978	0.2091	0.1652	0.0000

Table S2. Continued

Probe Set ID	Gene Symbol	Gene Title	RefSeq Transcript ID	Score(d)	Numerat-or(r)	Denominator(s+s0)	Fold Change	q-value(%)
Down reglulated								
213263_s_at	PCBP2	poly(rC) binding protein 2	NM_001098620 /// NM_001128911 /// NM_001128912 /// NM_001128913 /// NM_001128914 /// NM_005016 /// NM_031989	-3.0438	-0.9567	0.3143	0.5152	0.0622
213309_at	PLCL2	phospholipase C-like 2	NM_001144382 /// NM_015184	-9.5267	-2.8204	0.2960	0.1416	0.0000
213353_at	ABCA5	ATP-binding cassette, sub-family A (ABC1), member 5	NM_018672 /// NM_172232	-3.9434	-1.6257	0.4123	0.3240	0.0000
213363_at	CA5BP	Carbonic anhydrase VB pseudogene	NR_026551	-3.1597	-0.8101	0.2564	0.5704	0.0622
213385_at	CHN2	chimerin (chimaerin) 2	NM_001039936 /// NM_004067	-2.3100	-1.1446	0.4955	0.4523	0.1081
213391_at	DPY19L4	dpy-19-like 4 (C. elegans)	NM_181787	-2.3414	-0.8220	0.3511	0.5656	0.1081
213424_at	KIAA0895	KIAA0895	NM_001100425 /// NM_015314	-3.7222	-1.0014	0.2690	0.4995	0.0000
213427_at	RPP40	ribonuclease P/MRP 40kDa subunit	NM_006638	-7.5223	-1.2926	0.1718	0.4082	0.0000

Table S2. Continued

Probe Set ID	Gene Symbol	Gene Title	RefSeq Transcript ID	Score(d)	Numerat-or(r)	Denominator(s+s0)	Fold Change	q-value(%)
Down reglulated								
213436_at	CNR1	cannabinoid receptor 1 (brain)	NM_001160226 /// NM_001160258 /// NM_001160259 /// NM_001160260 /// NM_016083 /// NM_033181	-3.2875	-0.8189	0.2491	0.5669	0.0000
213469_at	PGAP1	post-GPI attachment to proteins 1	NM_024989	-4.7510	-0.9018	0.1898	0.5352	0.0000
213659_at	ZNF75D	zinc finger protein 75D	NM_001185063 /// NM_007131	-5.8532	-1.0622	0.1815	0.4789	0.0000
213693_s_at	MUC1	mucin 1, cell surface associated	NM_001018016 /// NM_001018017 /// NM_001044390 /// NM_001044391 /// NM_001044392 /// NM_001044393 /// NM_002456	-4.2652	-1.1288	0.2646	0.4573	0.0000
213712_at	ELOVL2	elongation of very long chain fatty acids (FEN1/Elo2, SUR4/Elo3, yeast)-like 2	NM_017770	-3.1401	-1.1016	0.3508	0.4660	0.0622

Table S2. Continued

Probe Set ID	Gene Symbol	Gene Title	RefSeq Transcript ID	Score(d)	Numerat-or(r)	Denominat or(s+s0)	Fold Change	q-value(%)
Down reglulated								
213908_at	WHAMML1 /// WHAMML2	WAS protein homolog associated with actin, golgi membranes and microtubules-like 1 (pseudogene) /// WAS protein homolog associated with actin, golgi membranes and microtubules-like 2 (pseudogene)	NR_003521 /// NR_026589	-5.2770	-1.1313	0.2144	0.4565	0.0000
213927_at	MAP3K9	mitogen-activated protein kinase kinase kinase 9	NM_033141	-3.0924	-0.9749	0.3153	0.5088	0.0622
213933_at	PTGER3	prostaglandin E receptor 3 (subtype EP3)	NM_001126044 /// NM_198714 /// NM_198715 /// NM_198716 /// NM_198717 /// NM_198718 /// NM_198719 /// NR_028292 /// NR_028293 /// NR_028294	-18.8713	-2.9765	0.1577	0.1271	0.0000
213935_at	ABHD5	abhydrolase domain containing 5	NM_016006	-3.0181	-0.8531	0.2827	0.5536	0.0622
213975_s_at	LYZ	lysozyme	NM_000239	-2.7289	-0.9208	0.3374	0.5282	0.0852
214110_s_at	NA	NA	NA	-3.9751	-1.3659	0.3436	0.3880	0.0000
214154_s_at	PKP2	plakophilin 2	NM_001005242 /// NM_004572	-3.7001	-0.8831	0.2387	0.5422	0.0000

Table S2. Continued

Probe Set ID	Gene Symbol	Gene Title	RefSeq Transcript ID	Score(d)	Numerat-or(r)	Denominat or(s+s0)	Fold Change	q-value(%)
Down reglulated								
214185_at	KHDRBS1	KH domain containing, RNA binding, signal transduction associated 1	NM_006559	-9.8986	-1.3100	0.1323	0.4033	0.0000
214220_s_at	ALMS1	Alstrom syndrome 1	NM_015120	-2.4890	-0.9092	0.3653	0.5325	0.1081
214270_s_at	MAPRE3	microtubule-associated protein, RP/EB family, member 3	NM_012326	-4.4495	-1.2573	0.2826	0.4183	0.0000
214358_at	ACACA	acetyl-CoA carboxylase alpha	NM_198834 /// NM_198836 /// NM_198837 /// NM_198838 /// NM_198839	-3.3390	-0.8329	0.2494	0.5614	0.0000
214433_s_at	SELENBP1	selenium binding protein 1	NM_003944	-4.3304	-0.9317	0.2151	0.5243	0.0000
214554_at	HIST1H2AL	histone cluster 1, H2al	NM_003511	-7.1358	-1.5948	0.2235	0.3311	0.0000
214663_at	DSTYK	dual serine/threonine and tyrosine protein kinase	NM_015375 /// NM_199462	-3.1997	-0.9037	0.2824	0.5345	0.0622
214681_at	GK	glycerol kinase	NM_000167 /// NM_001128127 /// NM_203391	-9.2629	-2.1437	0.2314	0.2263	0.0000
214721_x_at	CDC42EP4	CDC42 effector protein (Rho GTPase binding) 4	NM_012121	-4.4461	-1.2376	0.2783	0.4241	0.0000
214723_x_at	ANKRD36	ankyrin repeat domain 36	NM_001164315	-4.1783	-1.2576	0.3010	0.4182	0.0000
214808_at	NA	NA	NA	-3.1859	-1.0109	0.3173	0.4963	0.0622
214823_at	ZNF204P	zinc finger protein 204, pseudogene	NR_002722 /// NR_024553	-4.7746	-1.7983	0.3766	0.2875	0.0000

Table S2. Continued

Probe Set ID	Gene Symbol	Gene Title	RefSeq Transcript ID	Score(d)	Numerat-or(r)	Denominat or(s+s0)	Fold Change	q-value(%)
Down reglulated								
214877_at	CDKAL1	CDK5 regulatory subunit associated protein 1-like 1	NM_017774	-4.9002	-1.5049	0.3071	0.3524	0.0000
215051_x_at	AIF1	allograft inflammatory factor 1	NM_001623 /// NM_004847 /// NM_032955	-4.3155	-1.0214	0.2367	0.4926	0.0000
215099_s_at	RXRB	retinoid X receptor, beta	NM_021976	-2.2530	-0.9234	0.4099	0.5273	0.1081
215146_s_at	TTC28	tetratricopeptide repeat domain 28	NM_001145418	-7.4087	-1.4167	0.1912	0.3746	0.0000
215264_at	EMX1	empty spiracles homeobox 1	NM_004097	-4.7522	-0.9294	0.1956	0.5251	0.0000
215506_s_at	DIRAS3	DIRAS family, GTP-binding RAS-like 3	NM_004675	-7.7743	-2.6794	0.3446	0.1561	0.0000
215523_at	ZNF391	zinc finger protein 391	NM_001076781	-3.6765	-1.4181	0.3857	0.3742	0.0000
215695_s_at	GYG2	glycogenin 2	NM_001079855 /// NM_001184702 /// NM_001184703 /// NM_001184704 /// NM_003918	-5.2156	-1.7157	0.3290	0.3044	0.0000
215726_s_at	CYB5A	cytochrome b5 type A (microsomal)	NM_001190807 /// NM_001914 /// NM_148923	-17.9480	-4.8626	0.2709	0.0344	0.0000
215728_s_at	ACOT7	acyl-CoA thioesterase 7	NM_007274 /// NM_181864 /// NM_181865 /// NM_181866	-3.8149	-0.8775	0.2300	0.5443	0.0000
215765_at	LRRC41	leucine rich repeat containing 41	NM_006369	-5.6996	-0.9318	0.1635	0.5242	0.0000

Table S2. Continued

Probe Set ID	Gene Symbol	Gene Title	RefSeq Transcript ID	Score(d)	Numerat-or(r)	Denominator(s+s0)	Fold Change	q-value(%)
Down reglulated								
215881_x_at	SSX2 /// SSX2B /// SSX3	synovial sarcoma, X breakpoint 2 /// synovial sarcoma, X breakpoint 2B /// synovial sarcoma, X breakpoint 3	NM_001164417 /// NM_003147 /// NM_021014 /// NM_175698 /// NM_175711	-5.1000	-1.5465	0.3032	0.3424	0.0000
215891_s_at	GM2A	GM2 ganglioside activator	NM_000405 /// NM_001167607	-3.3064	-1.1030	0.3336	0.4655	0.0000
215966_x_at	GK3P	glycerol kinase 3 pseudogene	NR_026575	-2.3315	-0.9885	0.4240	0.5040	0.1081
215977_x_at	GK	glycerol kinase	NM_000167 /// NM_001128127 /// NM_203391	-7.8049	-2.2227	0.2848	0.2142	0.0000
216063_at	HBBP1	hemoglobin, beta pseudogene 1	NR_001589	-6.0339	-1.6389	0.2716	0.3211	0.0000
216218_s_at	PLCL2	phospholipase C-like 2	NM_001144382 /// NM_015184	-12.0572	-1.7978	0.1491	0.2876	0.0000
216221_s_at	PUM2	pumilio homolog 2 (Drosophila)	NM_015317	-4.7481	-0.9800	0.2064	0.5070	0.0000
216316_x_at	GK /// GK3P	glycerol kinase /// glycerol kinase 3 pseudogene	NM_000167 /// NM_001128127 /// NM_203391 /// NR_026575	-6.1674	-1.9665	0.3189	0.2559	0.0000
216379_x_at	CD24	CD24 molecule	NM_013230	-2.3184	-1.7678	0.7625	0.2937	0.1081
216438_s_at	TMSB4X /// TMSL3	thymosin beta 4, X-linked /// thymosin-like 3	NM_021109 /// NM_183049	-3.4864	-0.8833	0.2534	0.5421	0.0000

Table S2. Continued

Probe Set ID	Gene Symbol	Gene Title	RefSeq Transcript ID	Score(d)	Numerat-or(r)	Denominator(s+s0)	Fold Change	q-value(%)
Down reglulated								
216442_x_at	FN1	fibronectin 1	NM_002026 /// NM_054034 /// NM_212474 /// NM_212476 /// NM_212478 /// NM_212482	-3.1946	-1.1013	0.3447	0.4661	0.0622
216462_at	NA	NA	NA	-8.3679	-3.5989	0.4301	0.0825	0.0000
216471_x_at	SSX2 /// SSX2B	synovial sarcoma, X breakpoint 2 /// synovial sarcoma, X breakpoint 2B	NM_001164417 /// NM_003147 /// NM_175698	-12.9229	-4.8155	0.3726	0.0355	0.0000
217098_s_at	ZSCAN12	zinc finger and SCAN domain containing 12	NM_001163391 /// NR_028077	-4.8937	-0.8349	0.1706	0.5606	0.0000
217164_at	NA	NA	NA	-3.6132	-0.8090	0.2239	0.5708	0.0000
217167_x_at	GK	glycerol kinase	NM_000167 /// NM_001128127 /// NM_203391	-5.5246	-1.6560	0.2997	0.3173	0.0000
217299_s_at	NBN	nibrin	NM_002485	-4.6738	-0.8891	0.1902	0.5399	0.0000
217403_s_at	ZNF227	zinc finger protein 227	NM_182490	-2.7178	-0.8351	0.3073	0.5605	0.0852
217520_x_at	LOC283683	hypothetical LOC283683	XR_110612 /// XR_111481	-4.2709	-0.8388	0.1964	0.5591	0.0000
217564_s_at	CPS1	carbamoyl-phosphate synthase 1, mitochondrial	NM_001122633 /// NM_001122634 /// NM_001875	-3.7826	-1.6852	0.4455	0.3110	0.0000

Table S2. Continued

Probe Set ID	Gene Symbol	Gene Title	RefSeq Transcript ID	Score(d)	Numerat-or(r)	Denominator(s+s0)	Fold Change	q-value(%)
Down reglulated								
217662_x_at	NA	NA	NA	-2.2112	-1.0014	0.4529	0.4995	0.1519
217899_at	TMEM214	transmembrane protein 214	NM_001083590 /// NM_017727	-5.1457	-0.8360	0.1625	0.5602	0.0000
217923_at	PEF1	penta-EF-hand domain containing 1	NM_012392 /// NR_033686 /// NR_033688	-3.7947	-0.9178	0.2419	0.5293	0.0000
218005_at	ZNF22	zinc finger protein 22 (KOX 15)	NM_006963	-8.0029	-1.9745	0.2467	0.2545	0.0000
218006_s_at	ZNF22	zinc finger protein 22 (KOX 15)	NM_006963	-9.2944	-2.1008	0.2260	0.2331	0.0000
218062_x_at	CDC42EP4	CDC42 effector protein (Rho GTPase binding) 4	NM_012121	-3.7126	-0.8179	0.2203	0.5673	0.0000
218149_s_at	ZNF395	zinc finger protein 395	NM_018660	-7.1518	-1.2560	0.1756	0.4187	0.0000
218175_at	CCDC92	coiled-coil domain containing 92	NM_025140	-5.0814	-1.4651	0.2883	0.3622	0.0000
218219_s_at	LANCL2	LanC lantibiotic synthetase component C-like 2 (bacterial)	NM_018697	-4.9697	-1.2321	0.2479	0.4257	0.0000
218242_s_at	SUV420H1	suppressor of variegation 4-20 homolog 1 (Drosophila)	NM_016028 /// NM_017635	-4.8188	-1.0370	0.2152	0.4873	0.0000
218248_at	FAM111A	family with sequence similarity 111, member A	NM_001142519 /// NM_001142520 /// NM_001142521 /// NM_022074 /// NM_198847	-10.0355	-2.4061	0.2398	0.1887	0.0000
218311_at	MAP4K3	mitogen-activated protein kinase kinase kinase kinase 3	NM_003618	-3.7375	-0.9063	0.2425	0.5336	0.0000

Table S2. Continued

Probe Set ID	Gene Symbol	Gene Title	RefSeq Transcript ID	Score(d)	Numerat-or(r)	Denominator(s+s0)	Fold Change	q-value(%)
Down reglulated								
218423_x_at	VPS54	vacuolar protein sorting 54 homolog (S. cerevisiae)	NM_001005739 /// NM_016516	-5.1639	-0.8212	0.1590	0.5660	0.0000
218424_s_at	STEAP3	STEAP family member 3	NM_001008410 /// NM_018234 /// NM_182915	-8.3901	-1.2523	0.1493	0.4198	0.0000
218428_s_at	REV1	REV1 homolog (S. cerevisiae)	NM_001037872 /// NM_016316	-5.9181	-1.0535	0.1780	0.4818	0.0000
218435_at	DNAJC15	DnaJ (Hsp40) homolog, subfamily C, member 15	NM_013238	-6.0288	-2.6534	0.4401	0.1589	0.0000
218456_at	CAPRIN2	caprin family member 2	NM_001002259 /// NM_023925 /// NM_032156	-13.4075	-1.9985	0.1491	0.2503	0.0000
218480_at	AGBL5	ATP/GTP binding protein-like 5	NM_001035507 /// NM_021831	-3.0567	-0.8465	0.2769	0.5562	0.0622
218486_at	KLF11	Kruppel-like factor 11	NM_001177716 /// NM_001177718 /// NM_003597	-2.7191	-0.8883	0.3267	0.5403	0.0852
218523_at	LHPP	phospholysine phosphohistidine inorganic pyrophosphate phosphatase	NM_001167880 /// NM_022126	-3.5768	-0.8444	0.2361	0.5569	0.0000
218573_at	MAGEH1	melanoma antigen family H, 1	NM_014061	-5.2401	-1.0801	0.2061	0.4730	0.0000
218682_s_at	SLC4A1AP	solute carrier family 4 (anion exchanger), member 1, adaptor protein	NM_018158	-9.0454	-1.4196	0.1569	0.3738	0.0000

Table S2. Continued

Probe Set ID	Gene Symbol	Gene Title	RefSeq Transcript ID	Score(d)	Numerat-or(r)	Denominat or(s+s0)	Fold Change	q-value(%)
Down reglulated								
218692_at	SYBU	syntabulin (syntaxin-interacting)	NM_001099743 /// NM_001099744 /// NM_001099745 /// NM_001099746 /// NM_001099747 /// NM_001099748 /// NM_001099749 /// NM_001099750 /// NM_001099751 /// NM_001099752 /// NM_001099753 /// NM_001099754 /// NM_001099755 /// NM_001099756 /// NM_017786	-6.1967	-2.2917	0.3698	0.2042	0.0000
218694_at	ARMCX1	armadillo repeat containing, X-linked 1	NM_016608	-4.5275	-2.3072	0.5096	0.2020	0.0000
218723_s_at	C13orf15	chromosome 13 open reading frame 15	NM_014059	-4.0375	-1.0685	0.2646	0.4768	0.0000
218776_s_at	TMEM62	transmembrane protein 62	NM_024956	-3.7674	-0.8651	0.2296	0.5490	0.0000
218783_at	INTS7	integrator complex subunit 7	NM_015434	-3.0458	-0.9046	0.2970	0.5342	0.0622
218791_s_at	C15orf29	chromosome 15 open reading frame 29	NM_024713	-6.3533	-1.1783	0.1855	0.4419	0.0000
218836_at	RPP21 /// TRIM39 /// TRIM39R	ribonuclease P/MRP 21kDa subunit /// tripartite motif-containing 39 /// TRIM39-like protein	NM_021253 /// NM_024839 /// NM_172016	-6.4483	-0.8361	0.1297	0.5601	0.0000

Table S2. Continued

Probe Set ID	Gene Symbol	Gene Title	RefSeq Transcript ID	Score(d)	Numerat-or(r)	Denominator(s+s0)	Fold Change	q-value(%)
Down reglulated								
218838_s_at	TTC31	tetratricopeptide repeat domain 31	NM_022492 /// NR_027749	-3.9577	-1.1833	0.2990	0.4403	0.0000
218851_s_at	WDR33	WD repeat domain 33	NM_001006622 /// NM_001006623 /// NM_018383	-4.6873	-1.3810	0.2946	0.3840	0.0000
218857_s_at	ASRGL1	asparaginase like 1	NM_001083926 /// NM_025080	-5.2563	-1.2375	0.2354	0.4241	0.0000
218888_s_at	NETO2	neuropilin (NRP) and tolloid (TLL)-like 2	NM_018092	-4.5046	-1.4400	0.3197	0.3686	0.0000
218950_at	ARAP3	ArfGAP with RhoGAP domain, ankyrin repeat and PH domain 3	NM_022481	-5.2151	-1.9641	0.3766	0.2563	0.0000
218951_s_at	PLCXD1	phosphatidylinositol-specific phospholipase C, X domain containing 1	NM_018390 /// NR_028057	-5.8239	-0.9762	0.1676	0.5083	0.0000
218976_at	DNAJC12	DnaJ (Hsp40) homolog, subfamily C, member 12	NM_021800 /// NM_201262	-14.7612	-5.1660	0.3500	0.0279	0.0000
218992_at	C9orf46	chromosome 9 open reading frame 46	NM_018465	-14.4417	-3.4936	0.2419	0.0888	0.0000
219032_x_at	OPN3	opsin 3	NM_014322	-3.3854	-0.8413	0.2485	0.5582	0.0000
219034_at	PARP16	poly (ADP-ribose) polymerase family, member 16	NM_017851	-5.2675	-1.0126	0.1922	0.4957	0.0000
219043_s_at	LOC285359 /// PDCL3	phosducin-like 3 pseudogene /// phosducin-like 3	NM_024065 /// NR_002941	-7.2798	-1.2830	0.1762	0.4109	0.0000
219056_at	RNASEH2B	ribonuclease H2, subunit B	NM_001142279 /// NM_024570	-2.2203	-1.1011	0.4959	0.4662	0.1519

Table S2. Continued

Probe Set ID	Gene Symbol	Gene Title	RefSeq Transcript ID	Score(d)	Numerat-or(r)	Denominator(s+s0)	Fold Change	q-value(%)
Down reglulated								
219067_s_at	NSMCE4A	non-SMC element 4 homolog A (S. cerevisiae)	NM_001167865 /// NM_017615	-3.6717	-0.8761	0.2386	0.5448	0.0000
219077_s_at	WWOX	WW domain containing oxidoreductase	NM_016373 /// NM_130791 /// NM_130844	-6.8281	-1.7915	0.2624	0.2889	0.0000
219080_s_at	CTPS2	CTP synthase II	NM_001144002 /// NM_019857 /// NM_175859	-6.1340	-1.5032	0.2451	0.3528	0.0000
219109_at	SPAG16	sperm associated antigen 16	NM_001025436 /// NM_024532	-3.9290	-1.5272	0.3887	0.3469	0.0000
219126_at	PHF10	PHD finger protein 10	NM_018288 /// NM_133325	-2.9620	-0.8385	0.2831	0.5592	0.0622
219128_at	C2orf42	chromosome 2 open reading frame 42	NM_017880	-2.8891	-0.8359	0.2893	0.5603	0.0622
219156_at	SYNJ2BP	synaptojanin 2 binding protein	NM_018373	-3.2824	-1.0004	0.3048	0.4999	0.0000
219196_at	SCG3	secretogranin III	NM_001165257 /// NM_013243	-38.8569	-7.4725	0.1923	0.0056	0.0000
219235_s_at	PHACTR4	phosphatase and actin regulator 4	NM_001048183 /// NM_023923	-4.7642	-0.8854	0.1858	0.5414	0.0000
219278_at	MAP3K6	mitogen-activated protein kinase kinase kinase 6	NM_004672	-4.7304	-0.9506	0.2009	0.5174	0.0000
219320_at	MYO19	myosin XIX	NM_001033580 /// NM_001163735 /// NM_025109	-3.3513	-0.8151	0.2432	0.5684	0.0000

Table S2. Continued

Probe Set ID	Gene Symbol	Gene Title	RefSeq Transcript ID	Score(d)	Numerat-or(r)	Denominator(s+s0)	Fold Change	q-value(%)
Down reglulated								
219327_s_at	GPRC5C	G protein-coupled receptor, family C, group 5, member C	NM_018653 /// NM_022036	-4.2337	-0.8186	0.1934	0.5670	0.0000
219342_at	CASD1	CAS1 domain containing 1	NM_022900	-9.4572	-1.6767	0.1773	0.3128	0.0000
219376_at	ZNF322B	zinc finger protein 322B	NM_199005	-4.3056	-1.0193	0.2367	0.4933	0.0000
219405_at	TRIM68	tripartite motif-containing 68	NM_018073	-11.0867	-3.3417	0.3014	0.0986	0.0000
219410_at	TMEM45A	transmembrane protein 45A	NM_018004	-3.8155	-1.0059	0.2636	0.4980	0.0000
219495_s_at	ZNF180	zinc finger protein 180	NM_013256	-2.6581	-1.0033	0.3774	0.4989	0.0852
219607_s_at	MS4A4A	membrane-spanning 4-domains, subfamily A, member 4	NM_024021 /// NM_148975	-11.1870	-3.3929	0.3033	0.0952	0.0000
219648_at	MREG	melanoregulin	NM_018000	-10.0143	-2.9585	0.2954	0.1286	0.0000
219672_at	AHSP	alpha hemoglobin stabilizing protein	NM_016633	-5.9683	-1.2466	0.2089	0.4214	0.0000
219673_at	MCM9	minichromosome maintenance complex component 9	NM_153255	-4.4213	-0.8920	0.2018	0.5389	0.0000
219691_at	SAMD9	sterile alpha motif domain containing 9	NM_001193307 /// NM_017654	-6.7927	-0.9004	0.1326	0.5357	0.0000
219701_at	TMOD2	tropomodulin 2 (neuronal)	NM_001142885 /// NM_014548	-9.7285	-1.9755	0.2031	0.2543	0.0000
219718_at	FGGY	FGGY carbohydrate kinase domain containing	NM_001113411 /// NM_018291	-8.8508	-2.0790	0.2349	0.2367	0.0000
219730_at	MED18	mediator complex subunit 18	NM_001127350 /// NM_017638	-2.2298	-1.0809	0.4848	0.4727	0.1519
219754_at	RBM41	RNA binding motif protein 41	NM_001171080 /// NM_018301	-3.0284	-0.9386	0.3099	0.5217	0.0622

Table S2. Continued

Probe Set ID	Gene Symbol	Gene Title	RefSeq Transcript ID	Score(d)	Numerat-or(r)	Denominator(s+s0)	Fold Change	q-value(%)
Down reglulated								
219821_s_at	GFOD1	glucose-fructose oxidoreductase domain containing 1	NM_018988	-2.9467	-0.8381	0.2844	0.5594	0.0622
219932_at	SLC27A6	solute carrier family 27 (fatty acid transporter), member 6	NM_001017372 /// NM_014031	-2.6305	-1.0659	0.4052	0.4777	0.0852
219958_at	C20orf46	chromosome 20 open reading frame 46	NM_018354	-7.4953	-1.1527	0.1538	0.4498	0.0000
219969_at	TXLNG	taxilin gamma	NM_001168683 /// NM_018360	-4.6450	-1.1642	0.2506	0.4462	0.0000
219999_at	MAN2A2	mannosidase, alpha, class 2A, member 2	NM_006122	-2.9347	-0.9678	0.3298	0.5113	0.0622
220123_at	SLC35F5	solute carrier family 35, member F5	NM_025181	-3.4222	-1.0566	0.3088	0.4808	0.0000
220144_s_at	ANKRD5	ankyrin repeat domain 5	NM_022096 /// NM_198798	-4.6264	-1.2665	0.2738	0.4157	0.0000
220167_s_at	TP53TG3 /// TP53TG3B	TP53 target 3 /// TP53 target 3B	NM_001099687 /// NM_016212	-3.4675	-1.0246	0.2955	0.4915	0.0000
220180_at	CCDC68	coiled-coil domain containing 68	NM_001143829 /// NM_025214	-3.4203	-1.3177	0.3853	0.4012	0.0000
220230_s_at	CYB5R2	cytochrome b5 reductase 2	NM_016229	-19.8018	-4.3253	0.2184	0.0499	0.0000
220314_at	HSFX1 /// HSFX2	heat shock transcription factor family, X linked 1 /// heat shock transcription factor family, X linked 2	NM_001164415 /// NM_016153	-3.3919	-1.1449	0.3375	0.4522	0.0000
220319_s_at	MYLIP	myosin regulatory light chain interacting protein	NM_013262	-8.6052	-1.1720	0.1362	0.4438	0.0000
220367_s_at	SAP130	Sin3A-associated protein, 130kDa	NM_001145928 /// NM_024545	-4.8674	-0.9259	0.1902	0.5263	0.0000

Table S2. Continued

Probe Set ID	Gene Symbol	Gene Title	RefSeq Transcript ID	Score(d)	Numerat-or(r)	Denominator(s+s0)	Fold Change	q-value(%)
Down reglulated								
220382_s_at	ARHGAP28	Rho GTPase activating protein 28	NM_001010000	-2.2932	-0.8391	0.3659	0.5590	0.1081
220739_s_at	CNNM3	cyclin M3	NM_017623 /// NM_199078	-5.5131	-0.9085	0.1648	0.5327	0.0000
221121_at	CXorf48	chromosome X open reading frame 48	NM_001031705 /// NM_017863	-12.1880	-2.2236	0.1824	0.2141	0.0000
221123_x_at	ZNF395	zinc finger protein 395	NM_018660	-7.6335	-0.9440	0.1237	0.5198	0.0000
221142_s_at	PECR	peroxisomal trans-2-enoyl-CoA reductase	NM_018441	-4.3670	-0.9323	0.2135	0.5240	0.0000
221143_at	RPA4	replication protein A4, 30kDa	NM_013347	-7.0412	-1.5173	0.2155	0.3493	0.0000
221259_s_at	TEX11	testis expressed 11	NM_001003811 /// NM_031276	-4.8477	-1.1271	0.2325	0.4578	0.0000
221488_s_at	CUTA	cutA divalent cation tolerance homolog (E. coli)	NM_001014433 /// NM_001014837 /// NM_001014838 /// NM_001014840 /// NM_015921	-6.1274	-0.8413	0.1373	0.5581	0.0000
221582_at	HIST3H2A	histone cluster 3, H2a	NM_033445	-2.8812	-0.9488	0.3293	0.5181	0.0622
221620_s_at	APOO	apolipoprotein O	NM_024122 /// NR_026545	-7.2394	-1.3851	0.1913	0.3829	0.0000
221641_s_at	ACOT9	acyl-CoA thioesterase 9	NM_001033583 /// NM_001037171	-6.1260	-1.2784	0.2087	0.4122	0.0000
221808_at	RAB9A	RAB9A, member RAS oncogene family	NM_001195328 /// NM_004251	-5.5025	-1.1432	0.2078	0.4528	0.0000

Table S2. Continued

Probe Set ID	Gene Symbol	Gene Title	RefSeq Transcript ID	Score(d)	Numerat-or(r)	Denominator(s+s0)	Fold Change	q-value(%)
Down reglulated								
221816_s_at	PHF11	PHD finger protein 11	NM_001040443 /// NM_001040444	-2.8237	-0.9186	0.3253	0.5290	0.0622
221822_at	CCDC101	coiled-coil domain containing 101	NM_138414	-3.7835	-0.9461	0.2501	0.5190	0.0000
221893_s_at	ADCK2	aarF domain containing kinase 2	NM_052853	-5.5108	-1.2548	0.2277	0.4190	0.0000
221895_at	MOSPD2	motile sperm domain containing 2	NM_001177475 /// NM_152581	-2.3907	-1.0325	0.4319	0.4889	0.1081
221912_s_at	CCDC28B	coiled-coil domain containing 28B	NM_024296	-3.7359	-0.9340	0.2500	0.5234	0.0000
222018_at	NACA	nascent polypeptide-associated complex alpha subunit	NM_001113201 /// NM_001113202 /// NM_001113203 /// NM_005594	-3.3740	-0.8829	0.2617	0.5423	0.0000
222108_at	AMIGO2	adhesion molecule with Ig-like domain 2	NM_001143668 /// NM_181847	-4.8092	-1.7822	0.3706	0.2907	0.0000
222203_s_at	RDH14	retinol dehydrogenase 14 (all-trans/9-cis/11-cis)	NM_020905	-4.8610	-0.9863	0.2029	0.5048	0.0000
222270_at	SMEK2	SMEK homolog 2, suppressor of mek1 (Dictyostelium)	NM_001122964 /// NM_020463	-3.4940	-0.9263	0.2651	0.5262	0.0000
222305_at	HK2	hexokinase 2	NM_000189	-4.3230	-1.0206	0.2361	0.4929	0.0000
222324_at	NA	NA	NA	-3.6943	-0.8481	0.2296	0.5555	0.0000
222436_s_at	VPS24	vacuolar protein sorting 24 homolog (S. cerevisiae)	NM_001005753 /// NM_001193517 /// NM_016079 /// NR_036454	-3.5335	-0.8349	0.2363	0.5606	0.0000

Table S2. Continued

Probe Set ID	Gene Symbol	Gene Title	RefSeq Transcript ID	Score(d)	Numerat-or(r)	Denominator(s+s0)	Fold Change	q-value(%)
Down reglulated								
222437_s_at	VPS24	vacuolar protein sorting 24 homolog (S. cerevisiae)	NM_001005753 /// NM_001193517 /// NM_016079 /// NR_036454	-4.0126	-0.8348	0.2081	0.5606	0.0000
222566_at	SUV420H1	suppressor of variegation 4-20 homolog 1 (Drosophila)	NM_016028 /// NM_017635	-3.5892	-0.9843	0.2742	0.5055	0.0000
222627_at	VPS54	vacuolar protein sorting 54 homolog (S. cerevisiae)	NM_001005739 /// NM_016516	-5.2286	-1.0489	0.2006	0.4833	0.0000
222628_s_at	REV1	REV1 homolog (S. cerevisiae)	NM_001037872 /// NM_016316	-5.2107	-0.8790	0.1687	0.5437	0.0000
222629_at	REV1	REV1 homolog (S. cerevisiae)	NM_001037872 /// NM_016316	-3.7330	-1.4113	0.3781	0.3760	0.0000
222640_at	DNMT3A	DNA (cytosine-5-)-methyltransferase 3 alpha	NM_022552 /// NM_153759 /// NM_175629 /// NM_175630	-7.3117	-2.1007	0.2873	0.2331	0.0000
222662_at	PPP1R3B	protein phosphatase 1, regulatory (inhibitor) subunit 3B	NM_024607	-3.8283	-0.8505	0.2222	0.5546	0.0000
222687_s_at	ACER3	alkaline ceramidase 3	NM_018367	-2.8874	-0.9964	0.3451	0.5013	0.0622
222688_at	ACER3	alkaline ceramidase 3	NM_018367	-2.6976	-0.9491	0.3518	0.5179	0.0852
222689_at	ACER3	alkaline ceramidase 3	NM_018367	-3.8533	-0.8369	0.2172	0.5598	0.0000
222734_at	WARS2	tryptophanyl tRNA synthetase 2, mitochondrial	NM_015836 /// NM_201263	-2.1979	-0.8915	0.4056	0.5391	0.1519

Table S2. Continued

Probe Set ID	Gene Symbol	Gene Title	RefSeq Transcript ID	Score(d)	Numerator(r)	Denominator(s+s0)	Fold Change	q-value(%)
Down reglulated								
222763_s_at	WDR33	WD repeat domain 33	NM_001006622 /// NM_001006623 /// NM_018383	-4.7224	-1.1936	0.2527	0.4372	0.0000
222764_at	ASRGL1	asparaginase like 1	NM_001083926 /// NM_025080	-2.9415	-1.0486	0.3565	0.4834	0.0622
222774_s_at	NETO2	neuropilin (NRP) and tolloid (TLL)-like 2	NM_018092	-2.5534	-0.9029	0.3536	0.5348	0.1081
222795_s_at	PLCXD1	phosphatidylinositol-specific phospholipase C, X domain containing 1	NM_018390 /// NR_028057	-7.1069	-0.8794	0.1237	0.5436	0.0000
222819_at	CTPS2	CTP synthase II	NM_001144002 /// NM_019857 /// NM_175859	-9.3639	-2.1468	0.2293	0.2258	0.0000
222854_s_at	GEMIN8	gem (nuclear organelle) associated protein 8	NM_001042479 /// NM_001042480 /// NM_017856	-3.5605	-1.0091	0.2834	0.4969	0.0000
222872_x_at	OBFC2A	oligonucleotide/oligosaccharide-binding fold containing 2A	NM_001031716 /// NR_024415	-3.2950	-0.8907	0.2703	0.5393	0.0000
223044_at	SLC40A1	solute carrier family 40 (iron-regulated transporter), member 1	NM_014585	-4.2977	-1.5716	0.3657	0.3364	0.0000
223093_at	ANKH	ankylosis, progressive homolog (mouse)	NM_054027	-2.2329	-0.9027	0.4043	0.5349	0.1519
223095_at	MARVELD1	MARVEL domain containing 1	NM_031484	-3.6416	-0.9453	0.2596	0.5193	0.0000
223129_x_at	MYLIP	myosin regulatory light chain interacting protein	NM_013262	-6.1805	-1.1857	0.1918	0.4396	0.0000

Table S2. Continued

Probe Set ID	Gene Symbol	Gene Title	RefSeq Transcript ID	Score(d)	Numerat-or(r)	Denominator(s+s0)	Fold Change	q-value(%)
Down reglulated								
223130_s_at	MYLIP	myosin regulatory light chain interacting protein	NM_013262	-5.1089	-1.2409	0.2429	0.4231	0.0000
223158_s_at	NEK6	NIMA (never in mitosis gene a)-related kinase 6	NM_001145001 /// NM_001166167 /// NM_001166168 /// NM_001166169 /// NM_001166170 /// NM_001166171 /// NM_014397	-3.6271	-0.9638	0.2657	0.5127	0.0000
223159_s_at	NEK6	NIMA (never in mitosis gene a)-related kinase 6	NM_001145001 /// NM_001166167 /// NM_001166168 /// NM_001166169 /// NM_001166170 /// NM_001166171 /// NM_014397	-2.6676	-0.9494	0.3559	0.5179	0.0852
223182_s_at	AGPAT3	1-acylglycerol-3-phosphate O-acyltransferase 3	NM_001037553 /// NM_020132	-2.8680	-1.2366	0.4312	0.4244	0.0622
223183_at	AGPAT3	1-acylglycerol-3-phosphate O-acyltransferase 3	NM_001037553 /// NM_020132	-4.1406	-1.2813	0.3095	0.4114	0.0000
223184_s_at	AGPAT3	1-acylglycerol-3-phosphate O-acyltransferase 3	NM_001037553 /// NM_020132	-3.1674	-1.1959	0.3776	0.4365	0.0622
223216_x_at	ZNF395	zinc finger protein 395	NM_018660	-4.6517	-1.0995	0.2364	0.4667	0.0000

Table S2. Continued

Probe Set ID	Gene Symbol	Gene Title	RefSeq Transcript ID	Score(d)	Numerat-or(r)	Denominator(s+s0)	Fold Change	q-value(%)
Down reglulated								
223228_at	LDOC1L	leucine zipper, down-regulated in cancer 1-like	NM_032287	-5.3434	-1.8512	0.3464	0.2772	0.0000
223266_at	STRADB	STE20-related kinase adaptor beta	NM_018571	-6.2434	-1.5670	0.2510	0.3375	0.0000
223274_at	TCF19	transcription factor 19	NM_001077511 /// NM_007109	-4.9105	-0.8876	0.1808	0.5405	0.0000
223276_at	C5orf62	chromosome 5 open reading frame 62	NM_032947	-3.9469	-1.0713	0.2714	0.4759	0.0000
223297_at	AMMECR1L	AMME chromosomal region gene 1-like	NM_031445	-4.9057	-0.9908	0.2020	0.5032	0.0000
223305_at	TMEM216	transmembrane protein 216	NM_001173990 /// NM_001173991 /// NM_016499	-7.2422	-1.5310	0.2114	0.3460	0.0000
223378_at	GLIS2	GLIS family zinc finger 2	NM_032575	-3.0224	-0.8567	0.2834	0.5522	0.0622
223402_at	DUSP23	dual specificity phosphatase 23	NM_017823	-10.4526	-1.7509	0.1675	0.2971	0.0000
223423_at	GPR160	G protein-coupled receptor 160	NM_014373	-8.4241	-2.1661	0.2571	0.2228	0.0000
223437_at	PPARA	peroxisome proliferator-activated receptor alpha	NM_001001928 /// NM_005036	-4.1484	-1.3491	0.3252	0.3925	0.0000
223438_s_at	PPARA	peroxisome proliferator-activated receptor alpha	NM_001001928 /// NM_005036	-3.1720	-1.1700	0.3689	0.4444	0.0622
223482_at	TMEM120A	transmembrane protein 120A	NM_031925	-6.2061	-0.9690	0.1561	0.5108	0.0000
223532_at	ANKRD39	ankyrin repeat domain 39	NM_016466	-5.2486	-1.0526	0.2006	0.4821	0.0000
223611_s_at	LNX1	ligand of numb-protein X 1	NM_001126328 /// NM_032622	-4.1342	-1.4354	0.3472	0.3697	0.0000
223627_at	MEX3B	mex-3 homolog B (C. elegans)	NM_032246	-3.6472	-0.9559	0.2621	0.5155	0.0000

Table S2. Continued

Probe Set ID	Gene Symbol	Gene Title	RefSeq Transcript ID	Score(d)	Numerat-or(r)	Denominat or(s+s0)	Fold Change	q-value(%)
Down reglulated								
223638_at	NBPF3	neuroblastoma breakpoint family, member 3	NM_032264	-6.6666	-1.4740	0.2211	0.3600	0.0000
223710_at	CCL26	chemokine (C-C motif) ligand 26	NM_006072	-5.1414	-0.9636	0.1874	0.5128	0.0000
223721_s_at	DNAJC12	DnaJ (Hsp40) homolog, subfamily C, member 12	NM_021800 /// NM_201262	-10.0112	-3.4534	0.3450	0.0913	0.0000
223722_at	DNAJC12	DnaJ (Hsp40) homolog, subfamily C, member 12	NM_021800 /// NM_201262	-8.5034	-2.3946	0.2816	0.1902	0.0000
223735_at	ARL6	ADP-ribosylation factor-like 6	NM_032146 /// NM_177976	-6.1100	-1.5584	0.2551	0.3395	0.0000
223779_at	AFAP1-AS	AFAP1 antisense RNA (non-protein coding)	NR_026892	-17.4427	-2.8824	0.1652	0.1356	0.0000
223868_s_at	WWOX	WW domain containing oxidoreductase	NM_016373 /// NM_130791 /// NM_130844	-3.8410	-1.0959	0.2853	0.4678	0.0000
223977_s_at	C18orf2	chromosome 18 open reading frame 2	NR_023925 /// NR_023926 /// NR_023927 /// NR_023928	-4.2300	-0.9860	0.2331	0.5049	0.0000
224048_at	USP44	ubiquitin specific peptidase 44	NM_001042403 /// NM_032147	-3.3292	-1.4264	0.4284	0.3721	0.0000
224209_s_at	GDA	guanine deaminase	NM_004293	-6.6100	-1.8821	0.2847	0.2713	0.0000
224348_s_at	NA	NA	NA	-5.6721	-0.8729	0.1539	0.5461	0.0000
224392_s_at	OPN3	opsin 3	NM_014322	-2.3722	-0.8551	0.3605	0.5528	0.1081

Table S2. Continued

Probe Set ID	Gene Symbol	Gene Title	RefSeq Transcript ID	Score(d)	Numerat-or(r)	Denominat or(s+s0)	Fold Change	q-value(%)
Down reglulated								
224486_s_at	C15orf41	chromosome 15 open reading frame 41	NM_001130010 /// NM_032499	-6.8244	-0.9679	0.1418	0.5112	0.0000
224525_s_at	OLA1	Obg-like ATPase 1	NM_001011708 /// NM_013341	-2.3300	-0.8128	0.3488	0.5693	0.1081
224646_x_at	H19	H19, imprinted maternally expressed transcript (non-protein coding)	NR_002196	-9.2479	-3.1319	0.3387	0.1141	0.0000
224719_s_at	C12orf57	chromosome 12 open reading frame 57	NM_138425	-6.0570	-0.9390	0.1550	0.5216	0.0000
224772_at	NAV1	neuron navigator 1	NM_001167738 /// NM_020443	-6.7407	-1.4574	0.2162	0.3641	0.0000
224916_at	TMEM173	transmembrane protein 173	NM_198282	-5.5410	-1.2240	0.2209	0.4281	0.0000
224929_at	TMEM173	transmembrane protein 173	NM_198282	-5.8893	-1.1492	0.1951	0.4509	0.0000
224943_at	BTBD7	BTB (POZ) domain containing 7	NM_001002860 /// NM_018167	-2.2883	-0.8168	0.3570	0.5677	0.1081
224997_x_at	H19	H19, imprinted maternally expressed transcript (non-protein coding)	NR_002196	-8.1859	-2.6552	0.3244	0.1587	0.0000
225050_at	ZNF512	zinc finger protein 512	NM_032434	-4.2759	-0.8639	0.2020	0.5494	0.0000
225055_at	LOC100499466	hypothetical LOC100499466	NR_027418	-3.0738	-1.4636	0.4761	0.3626	0.0622
225060_at	LRP11	low density lipoprotein receptor-related protein 11	NM_032832	-15.1979	-3.2377	0.2130	0.1060	0.0000
225086_at	FAM98B	family with sequence similarity 98, member B	NM_001042429 /// NM_173611	-6.0464	-0.9740	0.1611	0.5091	0.0000

Table S2. Continued

Probe Set ID	Gene Symbol	Gene Title	RefSeq Transcript ID	Score(d)	Numerat-or(r)	Denominat or(s+s0)	Fold Change	q-value(%)
Down reglulated								
225136_at	PLEKHA2	pleckstrin homology domain containing, family A (phosphoinositide binding specific) member 2	NM_021623	-7.0136	-2.4349	0.3472	0.1849	0.0000
225154_at	SYAP1	synapse associated protein 1	NM_032796 /// NR_033181	-7.2665	-1.1987	0.1650	0.4357	0.0000
225164_s_at	EIF2AK4	eukaryotic translation initiation factor 2 alpha kinase 4	NM_001013703	-6.4646	-1.0239	0.1584	0.4918	0.0000
225207_at	PDK4	pyruvate dehydrogenase kinase, isozyme 4	NM_002612	-8.4311	-1.6389	0.1944	0.3211	0.0000
225217_s_at	BRPF3	bromodomain and PHD finger containing, 3	NM_015695	-3.7559	-0.8179	0.2178	0.5673	0.0000
225272_at	SAT2	spermidine/spermine N1-acetyltransferase family member 2	NM_133491	-3.2886	-0.8362	0.2543	0.5601	0.0000
225334_at	C10orf32	chromosome 10 open reading frame 32	NM_001136200 /// NM_144591	-3.9894	-1.1553	0.2896	0.4490	0.0000
225357_s_at	INO80	INO80 homolog (S. cerevisiae)	NM_017553	-5.0028	-0.9175	0.1834	0.5294	0.0000
225390_s_at	KLF13	Kruppel-like factor 13	NM_015995	-5.7686	-2.0278	0.3515	0.2452	0.0000
225415_at	DTX3L	deltex 3-like (Drosophila)	NM_138287	-3.3265	-1.2610	0.3791	0.4172	0.0000
225421_at	PM20D2	peptidase M20 domain containing 2	NM_001010853	-14.1237	-5.0254	0.3558	0.0307	0.0000
225431_x_at	PM20D2	peptidase M20 domain containing 2	NM_001010853	-6.9991	-2.0601	0.2943	0.2398	0.0000
225440_at	AGPAT3	1-acylglycerol-3-phosphate O-acyltransferase 3	NM_001037553 /// NM_020132	-4.0767	-0.8181	0.2007	0.5672	0.0000
225455_at	TADA1	transcriptional adaptor 1	NM_053053	-6.7114	-0.9743	0.1452	0.5090	0.0000
225487_at	TMEM18	transmembrane protein 18	NM_152834	-3.9343	-1.2459	0.3167	0.4216	0.0000

Table S2. Continued

Probe Set ID	Gene Symbol	Gene Title	RefSeq Transcript ID	Score(d)	Numerat-or(r)	Denominat or(s+s0)	Fold Change	q-value(%)
Down reglulated								
225489_at	TMEM18	transmembrane protein 18	NM_152834	-5.5722	-1.1474	0.2059	0.4514	0.0000
225567_at	NA	NA	NA	-3.8275	-1.0192	0.2663	0.4934	0.0000
225599_s_at	C8orf83	chromosome 8 open reading frame 83	NM_001171795 /// NM_001171796 /// NM_001171797 /// NM_001171798 /// NM_001171799 /// NM_001191035 /// NM_001191036	-3.3901	-1.6410	0.4841	0.3206	0.0000
225600_at	C8orf83	chromosome 8 open reading frame 83	NM_001171795 /// NM_001171796 /// NM_001171797 /// NM_001171798 /// NM_001171799 /// NM_001191035 /// NM_001191036	-2.8161	-1.0518	0.3735	0.4824	0.0622
225602_at	GLIPR2	GLI pathogenesis-related 2	NM_022343	-5.2669	-1.8652	0.3541	0.2745	0.0000

Table S2. Continued

Probe Set ID	Gene Symbol	Gene Title	RefSeq Transcript ID	Score(d)	Numerat-or(r)	Denominator(s+s0)	Fold Change	q-value(%)
Down reglulated								
225603_s_at	C8orf83	chromosome 8 open reading frame 83	NM_001171795 /// NM_001171796 /// NM_001171797 /// NM_001171798 /// NM_001171799 /// NM_001191035 /// NM_001191036	-2.5869	-0.9296	0.3594	0.5250	0.1081
225604_s_at	GLIPR2	GLI pathogenesis-related 2	NM_022343	-8.6795	-1.7412	0.2006	0.2991	0.0000
225606_at	BCL2L11	BCL2-like 11 (apoptosis facilitator)	NM_006538 /// NM_138621 /// NM_207002	-3.5670	-0.8150	0.2285	0.5684	0.0000
225612_s_at	B3GNT5	UDP-GlcNAc:betaGal beta-1,3-N-acetylglucosaminyltransferase 5	NM_032047	-3.6604	-0.8335	0.2277	0.5612	0.0000
225687_at	FAM83D	family with sequence similarity 83, member D	NM_030919	-3.4320	-0.8142	0.2372	0.5687	0.0000
225784_s_at	ZC4H2	zinc finger, C4H2 domain containing	NM_001178032 /// NM_001178033 /// NM_018684	-4.5971	-1.1936	0.2596	0.4372	0.0000
225844_at	POLE4	polymerase (DNA-directed), epsilon 4 (p12 subunit)	NM_019896	-4.4351	-1.0957	0.2471	0.4679	0.0000
225959_s_at	ZNRF1	zinc and ring finger 1	NM_032268	-5.0830	-0.8568	0.1686	0.5522	0.0000
225962_at	ZNRF1	zinc and ring finger 1	NM_032268	-2.7867	-1.1175	0.4010	0.4609	0.0852
226008_at	NDNL2	necdin-like 2	NM_138704	-5.4077	-0.8855	0.1638	0.5413	0.0000

Table S2. Continued

Probe Set ID	Gene Symbol	Gene Title	RefSeq Transcript ID	Score(d)	Numerat-or(r)	Denominat or(s+s0)	Fold Change	q-value(%)
Down reglulated								
226040_at	NA	NA	NA	-3.6954	-1.4893	0.4030	0.3562	0.0000
226041_at	NAPEPLD	N-acyl phosphatidylethanolamine phospholipase D	NM_001122838 /// NM_198990	-5.6426	-0.9088	0.1611	0.5326	0.0000
226068_at	SYK	spleen tyrosine kinase	NM_001135052 /// NM_001174167 /// NM_001174168 /// NM_003177	-4.4850	-0.8764	0.1954	0.5447	0.0000
226113_at	ZNF436	zinc finger protein 436	NM_001077195 /// NM_030634	-3.9017	-0.9615	0.2464	0.5135	0.0000
226114_at	ZNF436	zinc finger protein 436	NM_001077195 /// NM_030634	-3.5699	-0.9839	0.2756	0.5056	0.0000
226185_at	CDS1	CDP-diacylglycerol synthase (phosphatidate cytidylyltransferase) 1	NM_001263	-3.1708	-0.8098	0.2554	0.5705	0.0622
226186_at	TMOD2	tropomodulin 2 (neuronal)	NM_001142885 /// NM_014548	-17.5698	-2.9938	0.1704	0.1255	0.0000
226243_at	C2orf79	chromosome 2 open reading frame 79	NM_001013663	-6.2224	-1.0872	0.1747	0.4707	0.0000
226269_at	GDAP1	ganglioside-induced differentiation-associated protein 1	NM_001040875 /// NM_018972	-7.5831	-2.7972	0.3689	0.1439	0.0000
226271_at	GDAP1	ganglioside-induced differentiation-associated protein 1	NM_001040875 /// NM_018972	-4.2529	-1.6529	0.3886	0.3180	0.0000
226295_at	ITFG2	integrin alpha FG-GAP repeat containing 2	NM_018463	-3.1469	-0.8361	0.2657	0.5601	0.0622
226335_at	RPS6KA3	ribosomal protein S6 kinase, 90kDa, polypeptide 3	NM_004586	-3.8749	-0.8381	0.2163	0.5594	0.0000

Table S2. Continued

Probe Set ID	Gene Symbol	Gene Title	RefSeq Transcript ID	Score(d)	Numerat-or(r)	Denominator(s+s0)	Fold Change	q-value(%)
Down reglulated								
226337_at	GORAB	golgin, RAB6-interacting	NM_001146039 /// NM_152281 /// NR_027397	-4.5820	-0.8874	0.1937	0.5406	0.0000
226342_at	SPTBN1	spectrin, beta, non-erythrocytic 1	NM_003128 /// NM_178313	-2.6001	-1.3737	0.5283	0.3859	0.1081
226370_at	KLHL15	kelch-like 15 (Drosophila)	NM_030624	-5.0209	-1.2889	0.2567	0.4093	0.0000
226380_at	PTPN21	protein tyrosine phosphatase, non-receptor type 21	NM_007039	-3.8259	-0.9144	0.2390	0.5306	0.0000
226390_at	STARD4	StAR-related lipid transfer (START) domain containing 4	NM_139164	-7.6138	-1.2513	0.1643	0.4201	0.0000
226441_at	MAP3K2	mitogen-activated protein kinase kinase kinase 2	NM_006609	-3.1464	-1.1859	0.3769	0.4395	0.0622
226507_at	PAK1	p21 protein (Cdc42/Rac)-activated kinase 1	NM_001128620 /// NM_002576	-4.4051	-1.4548	0.3302	0.3648	0.0000
226562_at	ZSCAN29	zinc finger and SCAN domain containing 29	NM_152455	-3.8721	-0.8497	0.2195	0.5549	0.0000
226610_at	CENPV	centromere protein V	NM_181716	-7.9442	-1.7225	0.2168	0.3030	0.0000
226611_s_at	CENPV	centromere protein V	NM_181716	-10.3831	-1.8409	0.1773	0.2791	0.0000
226641_at	ANKRD44	ankyrin repeat domain 44	NM_001195144 /// NM_153697	-16.9654	-2.8884	0.1703	0.1350	0.0000
226745_at	CYP4V2	cytochrome P450, family 4, subfamily V, polypeptide 2	NM_207352	-4.3717	-1.4579	0.3335	0.3640	0.0000

Table S2. Continued

Probe Set ID	Gene Symbol	Gene Title	RefSeq Transcript ID	Score(d)	Numerat-or(r)	Denominator(s+s0)	Fold Change	q-value(%)
Down reglulated								
226748_at	LYSMD2	LysM, putative peptidoglycan-binding, domain containing 2	NM_001143917 /// NM_153374	-14.6930	-4.8814	0.3322	0.0339	0.0000
226752_at	FAM174A	family with sequence similarity 174, member A	NM_198507	-8.8529	-1.6427	0.1856	0.3203	0.0000
226760_at	MBTPS2	membrane-bound transcription factor peptidase, site 2	NM_015884	-3.6850	-0.9470	0.2570	0.5187	0.0000
226763_at	SESTD1	SEC14 and spectrin domains 1	NM_178123	-6.4376	-1.6775	0.2606	0.3126	0.0000
226775_at	ENY2	enhancer of yellow 2 homolog (Drosophila)	NM_001193557 /// NM_020189 /// NR_036471 /// NR_036472	-2.4433	-0.8647	0.3539	0.5492	0.1081
226809_at	LOC100216479	hypothetical LOC100216479	XR_109892 /// XR_112389	-4.9157	-1.0395	0.2115	0.4865	0.0000
226875_at	DOCK11	dedicator of cytokinesis 11	NM_144658	-4.3777	-0.9954	0.2274	0.5016	0.0000
226882_x_at	WDR4	WD repeat domain 4	NM_018669 /// NM_033661	-4.6192	-0.9985	0.2162	0.5005	0.0000
226912_at	ZDHHC23	zinc finger, DHHC-type containing 23	NM_173570	-2.7050	-0.8305	0.3070	0.5623	0.0852
226977_at	C5orf53	chromosome 5 open reading frame 53	NM_001007189	-4.9544	-1.1569	0.2335	0.4485	0.0000
226978_at	PPARA	peroxisome proliferator-activated receptor alpha	NM_001001928 /// NM_005036	-4.7011	-0.9288	0.1976	0.5253	0.0000
226979_at	MAP3K2	mitogen-activated protein kinase kinase kinase 2	NM_006609	-3.4727	-0.9283	0.2673	0.5255	0.0000

Table S2. Continued

Probe Set ID	Gene Symbol	Gene Title	RefSeq Transcript ID	Score(d)	Numerat-or(r)	Denominat or(s+s0)	Fold Change	q-value(%)
Down reglulated								
227002_at	FAM78A	family with sequence similarity 78, member A	NM_033387	-5.6305	-1.1389	0.2023	0.4541	0.0000
227041_at	SESTD1	SEC14 and spectrin domains 1	NM_178123	-2.9448	-0.8955	0.3041	0.5376	0.0622
227053_at	PACSIN1	protein kinase C and casein kinase substrate in neurons 1	NM_020804	-3.1390	-1.6147	0.5144	0.3265	0.0622
227083_at	B3GALTL	beta 1,3-galactosyltransferase-like	NM_194318	-3.3036	-1.1044	0.3343	0.4651	0.0000
227124_at	LOC221710	hypothetical protein LOC221710	NM_001135575	-4.6070	-0.9997	0.2170	0.5001	0.0000
227180_at	ELOVL7	ELOVL family member 7, elongation of long chain fatty acids (yeast)	NM_001104558 /// NM_024930	-3.0954	-1.3699	0.4426	0.3869	0.0622
227266_s_at	FYB	FYN binding protein	NM_001465 /// NM_199335	-18.6745	-5.1323	0.2748	0.0285	0.0000
227274_at	SYNJ2BP	synaptojanin 2 binding protein	NM_018373	-2.7378	-1.0321	0.3770	0.4890	0.0852
227330_x_at	LOC100132288 /// LOC100233156 /// MAFIP	hypothetical protein LOC100132288 /// hypothetical LOC100233156 /// MAFF interacting protein	NM_001033515 /// NM_001190825 /// XR_110796	-6.3518	-1.0882	0.1713	0.4703	0.0000
227353_at	TMC8	transmembrane channel-like 8	NM_152468	-2.6135	-0.8954	0.3426	0.5376	0.1081
227357_at	TAB3	TGF-beta activated kinase 1/MAP3K7 binding protein 3	NM_152787	-6.0095	-1.0119	0.1684	0.4959	0.0000
227361_at	HS3ST3B1	heparan sulfate (glucosamine) 3-O-sulfotransferase 3B1	NM_006041	-3.1236	-1.0150	0.3249	0.4948	0.0622
227395_at	NA	NA	NA	-11.2830	-2.4288	0.2153	0.1857	0.0000

Table S2. Continued

Probe Set ID	Gene Symbol	Gene Title	RefSeq Transcript ID	Score(d)	Numerat-or(r)	Denominator(s+s0)	Fold Change	q-value(%)
Down reglulated								
227409_at	PPP1R3E	protein phosphatase 1, regulatory (inhibitor) subunit 3E	NR_026862	-4.2048	-0.8534	0.2030	0.5535	0.0000
227412_at	PPP1R3E	protein phosphatase 1, regulatory (inhibitor) subunit 3E	NR_026862	-4.1842	-1.0733	0.2565	0.4752	0.0000
227426_at	SOS1	son of sevenless homolog 1 (Drosophila)	NM_005633	-3.0125	-0.8590	0.2852	0.5513	0.0622
227455_at	C6orf136	chromosome 6 open reading frame 136	NM_001109938 /// NM_001161376 /// NM_145029	-5.5259	-1.1757	0.2128	0.4427	0.0000
227456_s_at	C6orf136	chromosome 6 open reading frame 136	NM_001109938 /// NM_001161376 /// NM_145029	-4.4114	-0.9453	0.2143	0.5193	0.0000
227476_at	LPGAT1	lysophosphatidylglycerol acyltransferas 1	NM_014873	-6.2665	-1.2237	0.1953	0.4282	0.0000
227497_at	SOX6	SRY (sex determining region Y)-box 6	NM_001145811 /// NM_001145819 /// NM_017508 /// NM_033326	-4.6347	-1.3015	0.2808	0.4057	0.0000
227498_at	SOX6	SRY (sex determining region Y)-box 6	NM_001145811 /// NM_001145819 /// NM_017508 /// NM_033326	-3.6560	-1.4209	0.3887	0.3735	0.0000
227520_at	TXLNG	taxilin gamma	NM_001168683 /// NM_018360	-4.0085	-1.1201	0.2794	0.4601	0.0000

Table S2. Continued

Probe Set ID	Gene Symbol	Gene Title	RefSeq Transcript ID	Score(d)	Numerat-or(r)	Denominat or(s+s0)	Fold Change	q-value(%)
Down reglulated								
227567_at	LOC100499466	hypothetical LOC100499466	NR_027418	-7.2971	-2.8611	0.3921	0.1376	0.0000
227579_at	FER	fer (fps/fes related) tyrosine kinase	NM_005246	-14.1352	-2.3977	0.1696	0.1898	0.0000
227647_at	KCNE3	potassium voltage-gated channel, Isk-related family, member 3	NM_005472	-3.3244	-1.3796	0.4150	0.3843	0.0000
227693_at	WDR20	WD repeat domain 20	NM_144574 /// NM_181291 /// NM_181302 /// NM_181308	-3.0195	-0.8161	0.2703	0.5680	0.0622
227747_at	MPZL3	myelin protein zero-like 3	NM_198275	-2.6845	-1.2165	0.4532	0.4303	0.0852
227792_at	ITPRIPL2	inositol 1,4,5-triphosphate receptor interacting protein-like 2	NM_001034841 /// NR_028028	-8.4094	-2.1526	0.2560	0.2249	0.0000
227859_at	DNAJC27	DnaJ (Hsp40) homolog, subfamily C, member 27	NM_001198559 /// NM_016544	-2.3189	-0.8098	0.3492	0.5705	0.1081
227875_at	KLHL13	kelch-like 13 (Drosophila)	NM_001168299 /// NM_001168300 /// NM_001168301 /// NM_001168302 /// NM_001168303 /// NM_033495	-6.9761	-1.1848	0.1698	0.4399	0.0000
227903_x_at	C19orf20	chromosome 19 open reading frame 20	NM_033513	-6.9151	-1.0628	0.1537	0.4787	0.0000
227907_at	NA	NA	NA	-3.3018	-0.8398	0.2543	0.5587	0.0000
227911_at	ARHGAP28	Rho GTPase activating protein 28	NM_001010000	-5.5415	-1.3784	0.2487	0.3846	0.0000

Table S2. Continued

Probe Set ID	Gene Symbol	Gene Title	RefSeq Transcript ID	Score(d)	Numerat-or(r)	Denominator(s+s0)	Fold Change	q-value(%)
Down reglulated								
227917_at	FAM85A	family with sequence similarity 85, member A	XR_110590 /// XR_113294	-3.4855	-1.1557	0.3316	0.4489	0.0000
227933_at	LINGO1	leucine rich repeat and Ig domain containing 1	NM_032808	-3.4653	-1.1299	0.3261	0.4569	0.0000
227948_at	FGD4	FYVE, RhoGEF and PH domain containing 4	NM_139241	-8.3246	-1.9043	0.2288	0.2671	0.0000
227959_at	NA	NA	NA	-3.1047	-1.1939	0.3846	0.4371	0.0622
227983_at	RILPL2	Rab interacting lysosomal protein-like 2	NM_145058	-5.9879	-0.8928	0.1491	0.5386	0.0000
228005_at	ZXDB	zinc finger, X-linked, duplicated B	NM_007157	-3.8677	-1.0875	0.2812	0.4706	0.0000
228006_at	NA	NA	NA	-2.5014	-0.8485	0.3392	0.5553	0.1081
228011_at	FAM92A1	family with sequence similarity 92, member A1	NM_145269	-2.9440	-1.3408	0.4554	0.3948	0.0622
228088_at	SESTD1	SEC14 and spectrin domains 1	NM_178123	-3.2629	-0.8827	0.2705	0.5423	0.0000
228090_at	NMNAT3	nicotinamide nucleotide adenylyltransferase 3	NM_178177	-5.5281	-1.0675	0.1931	0.4771	0.0000
228097_at	MYLIP	myosin regulatory light chain interacting protein	NM_013262	-2.1573	-1.0251	0.4752	0.4914	0.1519
228098_s_at	MYLIP	myosin regulatory light chain interacting protein	NM_013262	-5.7515	-1.3315	0.2315	0.3973	0.0000
228099_at	ZNF550	zinc finger protein 550	NM_001039654	-3.9794	-0.8083	0.2031	0.5710	0.0000

Table S2. Continued

Probe Set ID	Gene Symbol	Gene Title	RefSeq Transcript ID	Score(d)	Numerat-or(r)	Denominator(s+s0)	Fold Change	q-value(%)
Down reglulated								
228158_at	LOC645166 /// LOC654342	lymphocyte-specific protein 1 pseudogene /// lymphocyte-specific protein 1 pseudogene	NR_027238 /// NR_027354 /// NR_027355 /// NR_027356	-4.5820	-1.1336	0.2474	0.4558	0.0000
228167_at	KLHL6	kelch-like 6 (Drosophila)	NM_130446	-3.7825	-0.9243	0.2444	0.5269	0.0000
228214_at	SOX6	SRY (sex determining region Y)-box 6	NM_001145811 /// NM_001145819 /// NM_017508 /// NM_033326	-2.7202	-1.1291	0.4151	0.4572	0.0852
228249_at	C11orf74	chromosome 11 open reading frame 74	NM_138787	-5.4894	-1.2766	0.2326	0.4128	0.0000
228316_at	C2orf63	chromosome 2 open reading frame 63	NM_001135598 /// NM_152385	-6.2787	-1.2507	0.1992	0.4202	0.0000
228361_at	E2F2	E2F transcription factor 2	NM_004091	-4.8336	-1.1046	0.2285	0.4650	0.0000
228375_at	IGSF11	immunoglobulin superfamily, member 11	NM_001015887 /// NM_152538	-5.6403	-1.0581	0.1876	0.4802	0.0000
228391_at	CYP4V2	cytochrome P450, family 4, subfamily V, polypeptide 2	NM_207352	-6.4093	-1.7704	0.2762	0.2931	0.0000
228415_at	AP1S2	adaptor-related protein complex 1, sigma 2 subunit	NM_003916	-5.4667	-1.4163	0.2591	0.3747	0.0000
228430_at	NA	NA	NA	-3.4219	-0.8797	0.2571	0.5435	0.0000
228434_at	BTNL9	butyrophilin-like 9	NM_152547	-9.1648	-2.1097	0.2302	0.2317	0.0000
228471_at	ANKRD44	ankyrin repeat domain 44	NM_001195144 /// NM_153697	-10.0858	-2.1613	0.2143	0.2236	0.0000

Table S2. Continued

Probe Set ID	Gene Symbol	Gene Title	RefSeq Transcript ID	Score(d)	Numerat-or(r)	Denominator(s+s0)	Fold Change	q-value(%)
Down reglulated								
228512_at	PTCD3	Pentatricopeptide repeat domain 3	NM_017952	-2.4907	-0.8168	0.3279	0.5677	0.1081
228516_at	CDAN1	congenital dyserythropoietic anemia type I	NM_138477	-6.7552	-0.9371	0.1387	0.5223	0.0000
228531_at	SAMD9	sterile alpha motif domain containing 9	NM_001193307 /// NM_017654	-6.9824	-1.8290	0.2619	0.2815	0.0000
228613_at	RAB11FIP3	RAB11 family interacting protein 3 (class II)	NM_001142272 /// NM_014700	-3.8465	-0.9904	0.2575	0.5033	0.0000
228618_at	PEAR1	platelet endothelial aggregation receptor 1	NM_001080471	-3.3150	-0.9334	0.2816	0.5236	0.0000
228624_at	TMEM144	transmembrane protein 144	NM_018342	-3.3592	-1.3324	0.3966	0.3971	0.0000
228708_at	RAB27B	RAB27B, member RAS oncogene family	NM_004163	-4.9132	-1.1078	0.2255	0.4640	0.0000
228765_at	GTF2IRD2	GTF2I repeat domain containing 2	NM_173537	-5.4226	-1.1002	0.2029	0.4664	0.0000
228812_at	NA	NA	NA	-6.9643	-1.0212	0.1466	0.4927	0.0000
228968_at	ZNF449	zinc finger protein 449	NM_152695	-2.7506	-0.8564	0.3114	0.5523	0.0852
228975_at	SP6	Sp6 transcription factor	NM_199262	-3.0707	-0.9583	0.3121	0.5147	0.0622
229001_at	PPP1R3E	protein phosphatase 1, regulatory (inhibitor) subunit 3E	NR_026862	-4.0997	-0.9499	0.2317	0.5177	0.0000
229022_at	ZFX	zinc finger protein, X-linked	NM_001178084 /// NM_001178085 /// NM_001178086 /// NM_001178095 /// NM_003410	-3.8897	-1.2569	0.3231	0.4184	0.0000
229103_at	WNT3	wingless-type MMTV integration site family, member 3	NM_030753	-3.8932	-1.0267	0.2637	0.4908	0.0000

Table S2. Continued

Probe Set ID	Gene Symbol	Gene Title	RefSeq Transcript ID	Score(d)	Numerat-or(r)	Denominat or(s+s0)	Fold Change	q-value(%)
Down reglulated								
229113_s_at	C1orf86	chromosome 1 open reading frame 86	NM_001146310 /// NM_182533	-4.9429	-0.8415	0.1703	0.5580	0.0000
229141_at	WDR33	WD repeat domain 33	NM_001006622 /// NM_001006623 /// NM_018383	-6.0336	-0.9283	0.1539	0.5255	0.0000
229189_s_at	LOC100507376	hypothetical LOC100507376	XR_108543 /// XR_108544 /// XR_112614 /// XR_112615 /// XR_113612 /// XR_113613	-3.7761	-1.0802	0.2861	0.4730	0.0000
229190_at	LOC100507376	hypothetical LOC100507376	XR_108543 /// XR_108544 /// XR_112614 /// XR_112615 /// XR_113612 /// XR_113613	-4.7213	-1.1849	0.2510	0.4399	0.0000
229208_at	HAUS2	HAUS augmin-like complex, subunit 2	NM_001130447 /// NM_018097	-3.8285	-0.8953	0.2339	0.5376	0.0000
229240_at	ZDHHC21	zinc finger, DHHC-type containing 21	NM_178566	-4.0821	-0.8900	0.2180	0.5396	0.0000
229312_s_at	GKAP1	G kinase anchoring protein 1	NM_001135953 /// NM_025211	-3.7228	-1.1237	0.3019	0.4589	0.0000

Table S2. Continued

Probe Set ID	Gene Symbol	Gene Title	RefSeq Transcript ID	Score(d)	Numerat-or(r)	Denominat or(s+s0)	Fold Change	q-value(%)
Down reglulated								
229450_at	IFIT3	interferon-induced protein with tetratricopeptide repeats 3	NM_001031683 /// NM_001549	-5.9404	-2.1278	0.3582	0.2288	0.0000
229455_at	NA	NA	NA	-3.6655	-1.0463	0.2855	0.4842	0.0000
229511_at	SMARCE1	SWI/SNF related, matrix associated, actin dependent regulator of chromatin, subfamily e, member 1	NM_003079	-2.6500	-0.8414	0.3175	0.5581	0.0852
229559_at	PPM1N	protein phosphatase, Mg2+/Mn2+ dependent, 1N (putative)	NM_001080401	-3.1951	-0.9536	0.2985	0.5164	0.0622
229602_at	NA	NA	NA	-3.8742	-1.0816	0.2792	0.4725	0.0000
229610_at	CKAP2L	cytoskeleton associated protein 2-like	NM_152515	-2.7147	-0.8217	0.3027	0.5658	0.0852
229620_at	NA	NA	NA	-8.2491	-2.4136	0.2926	0.1877	0.0000
229669_at	LOC100507263	hypothetical LOC100507263	XR_109374 /// XR_109375 /// XR_109376 /// XR_111674 /// XR_111675 /// XR_111676 /// XR_115068 /// XR_115069 /// XR_115070	-2.3693	-0.9523	0.4019	0.5168	0.1081
229716_at	NA	NA	NA	-5.1453	-0.8496	0.1651	0.5550	0.0000
229725_at	ACSL6	acyl-CoA synthetase long-chain family member 6	NM_001009185 /// NM_015256	-2.5004	-0.9341	0.3736	0.5234	0.1081

Table S2. Continued

Probe Set ID	Gene Symbol	Gene Title	RefSeq Transcript ID	Score(d)	Numerat-or(r)	Denominat or(s+s0)	Fold Change	q-value(%)
Down reglulated								
229748_x_at	LOC100132288	hypothetical protein LOC100132288	NM_001033515	-5.2887	-1.1325	0.2141	0.4561	0.0000
230016_at	C8orf38	chromosome 8 open reading frame 38	NM_152416	-2.6397	-0.8815	0.3339	0.5428	0.0852
230054_at	LOC100507547	hypothetical LOC100507547	NR_037169 /// NR_037170 /// NR_037171 /// NR_037172 /// XR_108705 /// XR_108706 /// XR_110801 /// XR_110802 /// XR_110957 /// XR_110958 /// XR_110980 /// XR_110981 /// XR_110992 /// XR_110993 /// XR_111004 /// XR_111005 /// XR_111016 /// XR_111017	-4.8560	-0.8612	0.1774	0.5505	0.0000
230092_at	UBXN10	UBX domain protein 10	NM_152376	-2.8073	-0.9997	0.3561	0.5001	0.0852
230179_at	LOC285812	hypothetical protein LOC285812	NA	-3.3734	-1.0563	0.3131	0.4809	0.0000

Table S2. Continued

Probe Set ID	Gene Symbol	Gene Title	RefSeq Transcript ID	Score(d)	Numerat-or(r)	Denominat or(s+s0)	Fold Change	q-value(%)
Down reglulated								
230187_s_at	LOC100507100	hypothetical LOC100507100	XR_108691	-2.1775	-1.0464	0.4806	0.4842	0.1519
230201_at	FXR1	fragile X mental retardation, autosomal homolog 1	NM_001013438 /// NM_001013439 /// NM_005087	-3.9214	-1.0172	0.2594	0.4941	0.0000
230337_at	SOS1	son of sevenless homolog 1 (Drosophila)	NM_005633	-3.1482	-0.9783	0.3107	0.5076	0.0622
230352_at	PRPS2	Phosphoribosyl pyrophosphate synthetase 2	NM_001039091 /// NM_002765	-16.9697	-4.6541	0.2743	0.0397	0.0000
230361_at	HEATR7A	HEAT repeat containing 7A	NM_001099280 /// NM_001099281 /// NM_032450	-3.9544	-0.8503	0.2150	0.5547	0.0000
230449_x_at	NA	NA	NA	-4.4911	-1.2094	0.2693	0.4325	0.0000
230563_at	RASGEF1A	RasGEF domain family, member 1A	NM_145313	-4.5780	-1.4880	0.3250	0.3565	0.0000
230644_at	LRFN5	leucine rich repeat and fibronectin type III domain containing 5	NM_152447	-3.1818	-1.2655	0.3977	0.4159	0.0622
230653_at	NA	NA	NA	-2.4041	-0.8249	0.3431	0.5645	0.1081
230712_at	NBPF1	neuroblastoma breakpoint family, member 1	NM_017940	-3.6738	-1.1766	0.3203	0.4424	0.0000
230758_at	GEMIN8	gem (nuclear organelle) associated protein 8	NM_001042479 /// NM_001042480 /// NM_017856	-4.0240	-1.1015	0.2737	0.4660	0.0000

Table S2. Continued

Probe Set ID	Gene Symbol	Gene Title	RefSeq Transcript ID	Score(d)	Numerat-or(r)	Denominator(s+s0)	Fold Change	q-value(%)
Down reglulated								
230781_at	LOC100505875	hypothetical LOC100505875	XR_109792 /// XR_109793 /// XR_109794 /// XR_109795 /// XR_109796 /// XR_112656 /// XR_112657 /// XR_112658 /// XR_112659 /// XR_112660 /// XR_113655 /// XR_113656 /// XR_113657 /// XR_113658 /// XR_113659	-3.2698	-1.1529	0.3526	0.4497	0.0000
230815_at	LOC389765	kinesin family member 27 pseudogene	NR_029410	-3.4103	-1.3787	0.4043	0.3846	0.0000
230836_at	ST8SIA4	ST8 alpha-N-acetyl-neuraminide alpha-2,8-sialyltransferase 4	NM_005668 /// NM_175052	-6.4841	-1.7010	0.2623	0.3076	0.0000
230951_at	EPB41L5	erythrocyte membrane protein band 4.1 like 5	NM_001184937 /// NM_001184938 /// NM_001184939 /// NM_020909	-4.5105	-0.9957	0.2208	0.5015	0.0000

Table S2. Continued

Probe Set ID	Gene Symbol	Gene Title	RefSeq Transcript ID	Score(d)	Numerat-or(r)	Denominat or(s+s0)	Fold Change	q-value(%)
Down reglulated								
230986_at	KLF8	Kruppel-like factor 8	NM_001159296 /// NM_007250	-2.7218	-0.8636	0.3173	0.5496	0.0852
231131_at	FAM133A	family with sequence similarity 133, member A	NM_001171109 /// NM_001171110 /// NM_001171111 /// NM_173698	-4.6482	-1.1265	0.2424	0.4580	0.0000
231265_at	COX7B2	cytochrome c oxidase subunit VIIb2	NM_130902	-8.5170	-2.0057	0.2355	0.2490	0.0000
231311_at	NA	NA	NA	-2.9303	-1.3007	0.4439	0.4059	0.0622
231407_s_at	FOXH1	forkhead box H1	NM_003923	-3.3327	-0.8649	0.2595	0.5491	0.0000
231669_at	SEPP1	Selenoprotein P, plasma, 1	NM_001085486 /// NM_001093726 /// NM_005410	-5.2020	-0.8684	0.1669	0.5478	0.0000
231769_at	FBXO6	F-box protein 6	NM_018438	-5.8157	-1.5192	0.2612	0.3489	0.0000
231857_s_at	AGBL5	ATP/GTP binding protein-like 5	NM_001035507 /// NM_021831	-3.0216	-0.8804	0.2914	0.5432	0.0622
231863_at	ING3	inhibitor of growth family, member 3	NM_019071 /// NM_198267	-4.9778	-1.5119	0.3037	0.3507	0.0000
231864_at	ZNF33A	zinc finger protein 33A	NM_006954 /// NM_006974	-2.5930	-0.8221	0.3170	0.5656	0.1081
231876_at	TRIM56	tripartite motif-containing 56	NM_030961	-4.0240	-0.9403	0.2337	0.5211	0.0000

Table S2. Continued

Probe Set ID	Gene Symbol	Gene Title	RefSeq Transcript ID	Score(d)	Numerat-or(r)	Denominator(s+s0)	Fold Change	q-value(%)
Down reglulated								
231924_at	LOC100506305	hypothetical LOC100506305	XR_109064 /// XR_109065 /// XR_109066 /// XR_109067 /// XR_109068 /// XR_111166 /// XR_111167 /// XR_111168 /// XR_111169 /// XR_111170 /// XR_114464 /// XR_114465 /// XR_114466 /// XR_114467 /// XR_114468	-3.4298	-0.9815	0.2862	0.5065	0.0000
231996_at	N4BP2	NEDD4 binding protein 2	NM_018177	-5.9504	-0.8425	0.1416	0.5577	0.0000
231997_at	TBCEL	tubulin folding cofactor E-like	NM_001130047 /// NM_152715	-3.4923	-1.3577	0.3888	0.3902	0.0000
232004_at	HNRNPR	heterogeneous nuclear ribonucleoprotein R	NM_001102397 /// NM_001102398 /// NM_001102399 /// NM_005826	-3.4515	-1.3139	0.3807	0.4022	0.0000
232064_at	FER	fer (fps/fes related) tyrosine kinase	NM_005246	-5.3199	-1.8361	0.3451	0.2801	0.0000

Table S2. Continued

Probe Set ID	Gene Symbol	Gene Title	RefSeq Transcript ID	Score(d)	Numerat-or(r)	Denominator(s+s0)	Fold Change	q-value(%)
Down reglulated								
232087_at	CXorf23	chromosome X open reading frame 23	NM_198279	-3.4742	-1.4682	0.4226	0.3614	0.0000
232109_at	UBXN10	UBX domain protein 10	NM_152376	-3.1491	-0.8406	0.2669	0.5584	0.0622
232395_x_at	AGBL3	ATP/GTP binding protein-like 3	NM_178563	-3.3540	-1.4453	0.4309	0.3672	0.0000
232506_s_at	C15orf41	chromosome 15 open reading frame 41	NM_001130010 /// NM_032499	-3.0308	-0.9147	0.3018	0.5305	0.0622
232682_at	MREG	melanoregulin	NM_018000	-4.9517	-1.4807	0.2990	0.3583	0.0000
232693_s_at	FBXO16 /// ZNF395	F-box protein 16 /// zinc finger protein 395	NM_018660 /// NM_172366	-3.2194	-0.9102	0.2827	0.5321	0.0622
232873_at	ZNF33A	zinc finger protein 33A	NM_006954 /// NM_006974	-2.5206	-0.8782	0.3484	0.5440	0.1081
233029_at	OBSCN	obscurin, cytoskeletal calmodulin and titin-interacting RhoGEF	NM_001098623 /// NM_052843	-5.9387	-0.9972	0.1679	0.5010	0.0000
233085_s_at	OBFC2A	oligonucleotide/oligosaccharide-binding fold containing 2A	NM_001031716 /// NR_024415	-3.8600	-0.8156	0.2113	0.5682	0.0000
233123_at	SLC40A1	Solute carrier family 40 (iron-regulated transporter), member 1	NM_014585	-3.8834	-0.8536	0.2198	0.5534	0.0000
233375_at	EFCAB2	EF-hand calcium binding domain 2	NM_001143943 /// NM_032328 /// NR_026586 /// NR_026587 /// NR_026588	-4.8216	-1.0270	0.2130	0.4907	0.0000
233514_x_at	TEX11	testis expressed 11	NM_001003811 /// NM_031276	-3.5770	-1.4749	0.4123	0.3598	0.0000

Table S2. Continued

Probe Set ID	Gene Symbol	Gene Title	RefSeq Transcript ID	Score(d)	Numerat-or(r)	Denominator(s+s0)	Fold Change	q-value(%)
Down reglulated								
233518_at	NA	NA	NA	-2.7897	-0.8573	0.3073	0.5520	0.0852
233562_at	LOC84856	hypothetical LOC84856	NR_026827	-4.5233	-1.2166	0.2690	0.4303	0.0000
233595_at	USP34	ubiquitin specific peptidase 34	NM_014709	-2.4297	-0.8173	0.3364	0.5675	0.1081
233638_s_at	POMGNT1	protein O-linked mannose beta1,2-N-acetylglucosaminyltransferase	NM_017739 /// NR_024332	-4.0745	-0.8342	0.2047	0.5609	0.0000
233656_s_at	VPS54	vacuolar protein sorting 54 homolog (S. cerevisiae)	NM_001005739 /// NM_016516	-8.0496	-0.9986	0.1241	0.5005	0.0000
234192_s_at	GKAP1	G kinase anchoring protein 1	NM_001135953 /// NM_025211	-5.7047	-1.6920	0.2966	0.3095	0.0000
234296_s_at	TEX11	testis expressed 11	NM_001003811 /// NM_031276	-4.9282	-1.2385	0.2513	0.4238	0.0000
234652_at	NA	NA	NA	-4.1102	-0.9596	0.2335	0.5142	0.0000
234704_at	NA	NA	NA	-2.6019	-0.8226	0.3162	0.5654	0.1081
234970_at	TC2N	tandem C2 domains, nuclear	NM_001128595 /// NM_001128596 /// NM_152332	-5.5444	-3.0572	0.5514	0.1201	0.0000
234974_at	GALM	galactose mutarotase (aldose 1-epimerase)	NM_138801	-4.5038	-0.9164	0.2035	0.5298	0.0000
235005_at	DIS3L	DIS3 mitotic control homolog (S. cerevisiae)-like	NM_001143688 /// NM_133375	-5.3912	-1.1646	0.2160	0.4461	0.0000
235027_at	NA	NA	NA	-7.6252	-1.9330	0.2535	0.2619	0.0000
235068_at	ZDHHC21	zinc finger, DHHC-type containing 21	NM_178566	-5.3532	-1.2377	0.2312	0.4240	0.0000

Table S2. Continued

Probe Set ID	Gene Symbol	Gene Title	RefSeq Transcript ID	Score(d)	Numerat-or(r)	Denominator(s+s0)	Fold Change	q-value(%)
Down reglulated								
235072_s_at	KIF13A	kinesin family member 13A	NM_001105566 /// NM_001105567 /// NM_001105568 /// NM_022113	-5.9912	-0.9425	0.1573	0.5203	0.0000
235074_at	SPRED1	sprouty-related, EVH1 domain containing 1	NM_152594	-4.5728	-1.0208	0.2232	0.4929	0.0000
235142_at	ZBTB8A	zinc finger and BTB domain containing 8A	NM_001040441	-9.0934	-2.2633	0.2489	0.2083	0.0000
235242_at	NA	NA	NA	-3.7964	-0.9281	0.2445	0.5255	0.0000
235256_s_at	GALM	galactose mutarotase (aldose 1-epimerase)	NM_138801	-3.1871	-1.1283	0.3540	0.4574	0.0622
235291_s_at	FLJ32255	hypothetical LOC643977	XR_108575 /// XR_112730 /// XR_113737	-4.2619	-1.8889	0.4432	0.2700	0.0000
235292_at	FLJ32255	hypothetical LOC643977	XR_108575 /// XR_112730 /// XR_113737	-7.4751	-2.1366	0.2858	0.2274	0.0000
235320_at	ARL6 /// LOC100506362	ADP-ribosylation factor-like 6 /// hypothetical LOC100506362	NM_032146 /// NM_177976 /// XR_108457 /// XR_112515 /// XR_113567	-3.5894	-1.0378	0.2891	0.4871	0.0000

Table S2. Continued

Probe Set ID	Gene Symbol	Gene Title	RefSeq Transcript ID	Score(d)	Numerat-or(r)	Denominator(s+s0)	Fold Change	q-value(%)
Down reglulated								
235349_at	FAM82A1	family with sequence similarity 82, member A1	NM_001170791 /// NM_001170792 /// NM_001170793 /// NM_144713	-4.1419	-1.0729	0.2590	0.4754	0.0000
235363_at	NA	NA	NA	-4.7512	-1.5804	0.3326	0.3344	0.0000
235382_at	AQPEP	laeverin	NM_173800	-5.3167	-1.7372	0.3267	0.2999	0.0000
235391_at	FAM92A1	family with sequence similarity 92, member A1	NM_145269	-3.7646	-1.5650	0.4157	0.3380	0.0000
235428_at	LOC100508176	hypothetical LOC100508176	XR_113188 /// XR_113189 /// XR_113190 /// XR_114230 /// XR_114231 /// XR_114232	-5.9806	-0.8453	0.1413	0.5566	0.0000
235482_at	LOC400960	hypothetical LOC400960	NR_033872	-5.0793	-1.0014	0.1972	0.4995	0.0000
235493_at	NA	NA	NA	-2.6036	-0.8727	0.3352	0.5461	0.1081
235509_at	C8orf38	chromosome 8 open reading frame 38	NM_152416	-3.7224	-0.9797	0.2632	0.5071	0.0000
235536_at	SNORD89	small nucleolar RNA, C/D box 89	NR_003070	-3.8024	-1.0220	0.2688	0.4924	0.0000
235542_at	TET3	tet oncogene family member 3	NM_144993	-6.5626	-1.1321	0.1725	0.4562	0.0000
235678_at	GM2A	GM2 ganglioside activator	NM_000405 /// NM_001167607	-4.0868	-1.2178	0.2980	0.4299	0.0000
235698_at	ZFP90	zinc finger protein 90 homolog (mouse)	NM_133458	-10.3440	-2.1909	0.2118	0.2190	0.0000

Table S2. Continued

Probe Set ID	Gene Symbol	Gene Title	RefSeq Transcript ID	Score(d)	Numerat-or(r)	Denominator(s+s0)	Fold Change	q-value(%)
Down reglulated								
235719_at	CYP4V2	cytochrome P450, family 4, subfamily V, polypeptide 2	NM_207352	-12.1125	-2.3272	0.1921	0.1993	0.0000
235729_at	ZNF514	zinc finger protein 514	NM_032788	-4.8349	-1.2650	0.2616	0.4161	0.0000
235737_at	TSLP	thymic stromal lymphopoietin	NM_033035 /// NR_033425	-3.8303	-1.6753	0.4374	0.3131	0.0000
235738_at	NA	NA	NA	-4.0428	-0.9822	0.2429	0.5062	0.0000
235911_at	MFI2	antigen p97 (melanoma associated) identified by monoclonal antibodies 133.2 and 96.5	NM_005929 /// NM_033316	-5.2180	-0.8917	0.1709	0.5390	0.0000
235981_at	C8orf22	chromosome 8 open reading frame 22	NM_001007176	-7.0218	-1.8214	0.2594	0.2829	0.0000
236128_at	ZNF91	zinc finger protein 91	NM_003430	-6.7250	-1.4213	0.2113	0.3734	0.0000
236165_at	NA	NA	NA	-8.5427	-1.2840	0.1503	0.4107	0.0000
236273_at	NBPF1	neuroblastoma breakpoint family, member 1	NM_017940	-3.0227	-0.8736	0.2890	0.5458	0.0622
236302_at	PPM1E	protein phosphatase, Mg2+/Mn2+ dependent, 1E	NM_014906	-9.2237	-2.5980	0.2817	0.1652	0.0000
236336_at	NA	NA	NA	-4.6247	-0.8289	0.1792	0.5629	0.0000
236488_s_at	NA	NA	NA	-4.6895	-0.8190	0.1746	0.5669	0.0000
236504_x_at	C6orf52	chromosome 6 open reading frame 52	NM_001145020 /// NR_026736 /// NR_026737	-5.1540	-1.2091	0.2346	0.4325	0.0000

Table S2. Continued

Probe Set ID	Gene Symbol	Gene Title	RefSeq Transcript ID	Score(d)	Numerat-or(r)	Denominat or(s+s0)	Fold Change	q-value(%)
Down reglulated								
236600_at	SPG20	spastic paraplegia 20 (Troyer syndrome)	NM_001142294 /// NM_001142295 /// NM_001142296 /// NM_015087	-3.1618	-0.9879	0.3124	0.5042	0.0622
236766_at	NA	NA	NA	-4.7453	-0.9437	0.1989	0.5199	0.0000
236777_at	LOC100129195	hypothetical LOC100129195	XR_108694 /// XR_112871 /// XR_113860	-2.3435	-0.8512	0.3632	0.5543	0.1081
236882_at	NA	NA	NA	-6.1111	-0.9016	0.1475	0.5353	0.0000
236981_at	C17orf99	chromosome 17 open reading frame 99	NM_001163075	-5.6146	-2.2848	0.4069	0.2052	0.0000
237247_at	USP51	ubiquitin specific peptidase 51	NM_201286	-2.9359	-0.8395	0.2859	0.5588	0.0622
237449_at	SP8	Sp8 transcription factor	NM_182700 /// NM_198956	-5.0366	-1.6293	0.3235	0.3232	0.0000
237739_at	NA	NA	NA	-3.6341	-0.9361	0.2576	0.5226	0.0000
237771_s_at	NA	NA	NA	-22.2272	-3.4410	0.1548	0.0921	0.0000
238013_at	PLEKHA2	pleckstrin homology domain containing, family A (phosphoinositide binding specific) member 2	NM_021623	-6.9458	-1.6791	0.2417	0.3123	0.0000
238025_at	MLKL	mixed lineage kinase domain-like	NM_001142497 /// NM_152649	-4.6190	-1.4487	0.3136	0.3664	0.0000
238356_at	DOCK11	dedicator of cytokinesis 11	NM_144658	-3.2575	-1.0924	0.3353	0.4690	0.0000
238435_at	LOC100506661	hypothetical LOC100506661	XR_110931	-2.4890	-0.9495	0.3815	0.5178	0.1081

Table S2. Continued

Probe Set ID	Gene Symbol	Gene Title	RefSeq Transcript ID	Score(d)	Numerat-or(r)	Denominator(s+s0)	Fold Change	q-value(%)
Down reglulated								
238599_at	IRAK1BP1	interleukin-1 receptor-associated kinase 1 binding protein 1	NM_001010844	-7.4883	-1.9233	0.2568	0.2637	0.0000
238657_at	UBXN10	UBX domain protein 10	NM_152376	-5.9971	-2.1496	0.3584	0.2254	0.0000
238668_at	NA	NA	NA	-3.6219	-1.2481	0.3446	0.4210	0.0000
238717_at	NA	NA	NA	-4.6061	-0.8198	0.1780	0.5665	0.0000
238722_x_at	NAPEPLD	N-acyl phosphatidylethanolamine phospholipase D	NM_001122838 /// NM_198990	-3.5873	-0.8269	0.2305	0.5637	0.0000
238768_at	C2orf68	chromosome 2 open reading frame 68	NM_001013649	-3.7084	-0.8539	0.2303	0.5533	0.0000
238969_at	C3orf55	chromosome 3 open reading frame 55	NM_001099777 /// NM_001130001 /// NM_001130002 /// NR_024016	-3.6823	-1.0275	0.2790	0.4906	0.0000
239106_at	CA5BP	Carbonic anhydrase VB pseudogene	NR_026551	-3.9100	-1.2345	0.3157	0.4250	0.0000
239133_at	CTDSPL2	CTD (carboxy-terminal domain, RNA polymerase II, polypeptide A) small phosphatase like 2	NM_016396	-6.7628	-0.9049	0.1338	0.5341	0.0000

Table S2. Continued

Probe Set ID	Gene Symbol	Gene Title	RefSeq Transcript ID	Score(d)	Numerat-or(r)	Denominat or(s+s0)	Fold Change	q-value(%)
Down reglulated								
239169_at	RDM1	RAD52 motif 1	NM_001034836 /// NM_001163120 /// NM_001163121 /// NM_001163122 /// NM_001163124 /// NM_001163125 /// NM_001163130 /// NM_145654 /// NR_027996 /// NR_027997 /// NR_027998 /// NR_027999 /// NR_028000	-2.6719	-1.5421	0.5771	0.3434	0.0852
239277_at	NA	NA	NA	-6.2859	-1.5731	0.2503	0.3361	0.0000
239410_at	NA	NA	NA	-3.6912	-1.0320	0.2796	0.4890	0.0000
239417_x_at	C6orf52	chromosome 6 open reading frame 52	NM_001145020 /// NR_026736 /// NR_026737	-6.3028	-1.0307	0.1635	0.4895	0.0000
239538_at	ZRANB3	zinc finger, RAN-binding domain containing 3	NM_032143	-9.1862	-1.5183	0.1653	0.3491	0.0000
239680_at	WDR76	WD repeat domain 76	NM_001167941 /// NM_024908	-2.7665	-0.8681	0.3138	0.5479	0.0852

Table S2. Continued

Probe Set ID	Gene Symbol	Gene Title	RefSeq Transcript ID	Score(d)	Numerat-or(r)	Denominator(s+s0)	Fold Change	q-value(%)
Down reglulated								
239711_at	ADAL	adenosine deaminase-like	NM_001012969 /// NM_001159280	-4.0061	-0.8872	0.2215	0.5407	0.0000
239847_at	NA	NA	NA	-3.1332	-0.8561	0.2732	0.5525	0.0622
240121_x_at	NA	NA	NA	-5.9266	-1.5070	0.2543	0.3518	0.0000
240214_at	RWDD1	RWD domain containing 1	NM_001007464 /// NM_015952 /// NM_016104	-2.7290	-0.8429	0.3089	0.5575	0.0852
240368_at	NA	NA	NA	-2.5830	-0.8886	0.3440	0.5401	0.1081
240687_at	PASD1	PAS domain containing 1	NM_173493	-3.4240	-0.8291	0.2422	0.5629	0.0000
241017_at	RPL31 /// TBC1D8	ribosomal protein L31 /// TBC1 domain family, member 8 (with GRAM domain)	NM_000993 /// NM_001098577 /// NM_001099693 /// NM_001102426	-3.0309	-0.8846	0.2918	0.5417	0.0622
241074_at	SKAP2	Src kinase associated phosphoprotein 2	NM_003930	-3.9082	-0.9234	0.2363	0.5273	0.0000
241416_at	NA	NA	NA	-3.8840	-1.1769	0.3030	0.4423	0.0000
241469_at	HFM1	HFM1, ATP-dependent DNA helicase homolog (S. cerevisiae)	NM_001017975	-5.0200	-1.0987	0.2189	0.4669	0.0000
241716_at	HSPD1	heat shock 60kDa protein 1 (chaperonin)	NM_002156 /// NM_199440	-2.7683	-0.8350	0.3016	0.5606	0.0852
241946_at	ZDHHC21	zinc finger, DHHC-type containing 21	NM_178566	-4.0405	-0.9213	0.2280	0.5280	0.0000
242218_at	LOC100509629	hypothetical LOC100509629	XR_112866 /// XR_113883	-4.4145	-1.2999	0.2945	0.4061	0.0000

Table S2. Continued

Probe Set ID	Gene Symbol	Gene Title	RefSeq Transcript ID	Score(d)	Numerat-or(r)	Denominator(s+s0)	Fold Change	q-value(%)
Down reglulated								
242263_at	TMED5	transmembrane emp24 protein transport domain containing 5	NM_001167830 /// NM_016040 /// NR_030761	-2.6684	-0.8357	0.3132	0.5603	0.0852
242293_at	ING3	inhibitor of growth family, member 3	NM_019071 /// NM_198267	-2.2588	-0.8509	0.3767	0.5544	0.1081
242427_at	WAC	WW domain containing adaptor with coiled-coil	NM_016628 /// NM_100486 /// NR_024557	-3.4220	-0.9974	0.2915	0.5009	0.0000
242519_at	CCDC152	coiled-coil domain containing 152	NM_001134848	-10.4812	-2.7075	0.2583	0.1531	0.0000
242601_at	HEPACAM2	HEPACAM family member 2	NM_001039372 /// NM_198151	-5.0186	-4.1773	0.8324	0.0553	0.0000
242917_at	RASGEF1A	RasGEF domain family, member 1A	NM_145313	-2.2510	-0.8420	0.3741	0.5579	0.1081
242943_at	ST8SIA4	ST8 alpha-N-acetyl-neuraminide alpha-2,8-sialyltransferase 4	NM_005668 /// NM_175052	-4.3786	-1.0806	0.2468	0.4728	0.0000
243110_x_at	NPW	neuropeptide W	NM_001099456	-4.4652	-0.8668	0.1941	0.5484	0.0000
243179_at	LOC100130360	Hypothetical LOC100130360	NA	-5.2028	-1.9528	0.3753	0.2583	0.0000
243209_at	KCNQ4	potassium voltage-gated channel, KQT-like subfamily, member 4	NM_004700 /// NM_172163	-5.1391	-1.4275	0.2778	0.3718	0.0000

Table S2. Continued

Probe Set ID	Gene Symbol	Gene Title	RefSeq Transcript ID	Score(d)	Numerat-or(r)	Denominator(s+s0)	Fold Change	q-value(%)
Down reglulated								
243299_at	VRK2	Vaccinia related kinase 2	NM_001130480 /// NM_001130481 /// NM_001130482 /// NM_001130483 /// NM_006296 /// NR_036441	-3.6338	-0.8778	0.2416	0.5442	0.0000
243478_at	NA	NA	NA	-2.8459	-0.8396	0.2950	0.5588	0.0622
243729_at	NA	NA	NA	-3.6662	-1.0130	0.2763	0.4955	0.0000
243943_x_at	C6orf52	chromosome 6 open reading frame 52	NM_001145020 /// NR_026736 /// NR_026737	-4.8910	-1.2160	0.2486	0.4305	0.0000
243996_at	NA	NA	NA	-3.4982	-0.8574	0.2451	0.5520	0.0000
244360_at	FBXL17	F-box and leucine-rich repeat protein 17	NM_001163315	-7.5310	-1.2742	0.1692	0.4134	0.0000
244406_at	ZNF20 /// ZNF625	zinc finger protein 20 /// zinc finger protein 625	NM_021143 /// NM_145233	-4.7923	-1.1011	0.2298	0.4662	0.0000
244570_at	NA	NA	NA	-6.8797	-1.6028	0.2330	0.3292	0.0000
244758_at	SCAND3	SCAN domain containing 3	NM_052923	-3.0750	-0.8254	0.2684	0.5643	0.0622
244764_at	LOC100507198	hypothetical LOC100507198	XR_110503 /// XR_112054 /// XR_113348	-2.5537	-1.0188	0.3989	0.4935	0.1081
244802_at	NA	NA	NA	-10.1533	-2.2926	0.2258	0.2041	0.0000
33322_i_at	SFN	stratifin	NM_006142	-5.1538	-0.8242	0.1599	0.5648	0.0000
33323_r_at	SFN	stratifin	NM_006142	-7.6744	-1.5658	0.2040	0.3378	0.0000

Table S2. Continued

Probe Set ID	Gene Symbol	Gene Title	RefSeq Transcript ID	Score(d)	Numerat-or(r)	Denominat or(s+s0)	Fold Change	q-value(%)
Down reglulated								
34210_at	CD52	CD52 molecule	NM_001803	-7.6248	-1.8541	0.2432	0.2766	0.0000
35820_at	GM2A	GM2 ganglioside activator	NM_000405 /// NM_001167607	-5.4260	-1.1798	0.2174	0.4414	0.0000
35974_at	LRMP	lymphoid-restricted membrane protein	NM_006152	-3.0668	-0.8924	0.2910	0.5387	0.0622
37152_at	PPARD	peroxisome proliferator-activated receptor delta	NM_001171818 /// NM_001171819 /// NM_001171820 /// NM_006238 /// NM_177435	-4.3391	-1.0826	0.2495	0.4722	0.0000
41577_at	PPP1R16B	protein phosphatase 1, regulatory (inhibitor) subunit 16B	NM_001172735 /// NM_015568	-2.5713	-0.9682	0.3765	0.5111	0.1081
44040_at	FBXO41	F-box protein 41	NM_001080410	-8.1406	-1.3081	0.1607	0.4039	0.0000
44120_at	ADCK2	aarF domain containing kinase 2	NM_052853	-4.5480	-1.2016	0.2642	0.4348	0.0000
46665_at	SEMA4C	sema domain, immunoglobulin domai (Ig), transmembrane domain (TM) and short cytoplasmic domain, (semaphorin) 4C	NM_017789	-3.8755	-1.0520	0.2715	0.4823	0.0000
53720_at	C19orf66	chromosome 19 open reading frame 66	NM_018381	-4.5396	-1.2229	0.2694	0.4284	0.0000
59433_at	LOC389906	hypothetical LOC389906	NR_034031	-2.6034	-0.8797	0.3379	0.5435	0.1081
64883_at	MOSPD2	motile sperm domain containing 2	NM_001177475 /// NM_152581	-2.6320	-1.0072	0.3827	0.4975	0.0852
65472_at	C2orf68	chromosome 2 open reading frame 68	NM_001013649	-3.2780	-0.9261	0.2825	0.5263	0.0000
74694_s_at	RABEP2	rabaptin, RAB GTPase binding effector protein 2	NM_024816	-3.4080	-1.5091	0.4428	0.3513	0.0000
