# Supplementary material for: Anticancer Agent Shikonin Is an Incompetent Inducer of Cancer Drug Resistance
Source: PLoS One. 2013 Jan 3;8(1):e52706. doi: 10.1371/journal.pone.0052706 (PMC3536779; doi:10.1371/journal.pone.0052706)
Supplement: Table S3 — The common gene ontology terms of up regulated genes in K562/Shk cells. Cells were treated with shikonin for 18 months, control cells were treated with vehicle as described in Materials and Methods. (PDF) [file pone.0052706.s003.pdf]

Table S3: The common gene ontology terms of up regulated genes in K562/Shk cells. Cells were treated with shikonin for 18 months, control cells were treated with vehicle as described in Materials and Methods.

GO ID	GO Term	Count	PValue	Fold Enrichment
Biological Process				
GO:0042127	regulation of cell proliferation	24	0.0128	1.7118
GO:0043067	regulation of programmed cell death	24	0.0179	1.6591
GO:0010941	regulation of cell death	24	0.0186	1.6530
GO:0042981	regulation of apoptosis	23	0.0287	1.6058
GO:0010033	response to organic substance	22	0.0177	1.7128
GO:0055114	oxidation reduction	21	0.0100	1.8447
GO:0042592	homeostatic process	21	0.0456	1.5696
GO:0007267	cell-cell signaling	20	0.0106	1.8711
GO:0007049	cell cycle	20	0.0965	1.4467
GO:0031328	positive regulation of cellular biosynthetic process	19	0.0625	1.5570
GO:0009891	positive regulation of biosynthetic process	19	0.0697	1.5346
GO:0009611	response to wounding	18	0.0133	1.9064
GO:0010557	positive regulation of macromolecule biosynthetic process	18	0.0746	1.5449
GO:0009719	response to endogenous stimulus	16	0.0058	2.2176
GO:0007167	enzyme linked receptor protein signaling pathway	15	0.0032	2.4620
GO:0030030	cell projection organization	15	0.0061	2.2880
GO:0030182	neuron differentiation	15	0.0249	1.9224
GO:0042325	regulation of phosphorylation	15	0.0386	1.8068
GO:0051174	regulation of phosphorus metabolic process	15	0.0507	1.7361
GO:0019220	regulation of phosphate metabolic process	15	0.0507	1.7361
GO:0001944	vasculature development	14	0.0006	3.1309
GO:0001775	cell activation	14	0.0019	2.7382
GO:0009725	response to hormone stimulus	14	0.0140	2.1413
GO:0051338	regulation of transferase activity	14	0.0156	2.1125
GO:0008284	positive regulation of cell proliferation	14	0.0333	1.8982
GO:0001568	blood vessel development	13	0.0014	2.9785
GO:0043549	regulation of kinase activity	13	0.0257	2.0441
GO:0043069	negative regulation of programmed cell death	13	0.0266	2.0327
GO:0060548	negative regulation of cell death	13	0.0274	2.0270

Table S3: Continued

GO ID	GO Term	Count	PValue	Fold Enrichment
GO:0043068	positive regulation of programmed cell death	13	0.0850	1.6853
GO:0010942	positive regulation of cell death	13	0.0873	1.6775
GO:0007010	cytoskeleton organization	13	0.0885	1.6737
GO:0044092	negative regulation of molecular function	12	0.0363	2.0167
GO:0048666	neuron development	12	0.0397	1.9870
GO:0045859	regulation of protein kinase activity	12	0.0441	1.9524
GO:0043066	negative regulation of apoptosis	12	0.0514	1.9028
GO:0032989	cellular component morphogenesis	12	0.0969	1.6967
GO:0010035	response to inorganic substance	11	0.0036	3.0120
GO:0031175	neuron projection development	11	0.0161	2.4120
GO:0003006	reproductive developmental process	11	0.0187	2.3567
GO:0019226	transmission of nerve impulse	11	0.0943	1.7642
GO:0009991	response to extracellular stimulus	10	0.0167	2.5515
GO:0048584	positive regulation of response to stimulus	10	0.0251	2.3785
GO:0045321	leukocyte activation	10	0.0289	2.3195
GO:0000904	cell morphogenesis involved in differentiation	10	0.0298	2.3005
GO:0060341	regulation of cellular localization	10	0.0330	2.2634
GO:0043086	negative regulation of catalytic activity	10	0.0590	2.0265
GO:0051094	positive regulation of developmental process	10	0.0601	2.0192
GO:0042060	wound healing	9	0.0206	2.6450
GO:0048545	response to steroid hormone stimulus	9	0.0213	2.6312
GO:0019637	organophosphate metabolic process	9	0.0263	2.5260
GO:0048514	blood vessel morphogenesis	9	0.0346	2.3943
GO:0033043	regulation of organelle organization	9	0.0398	2.3281
GO:0007169	transmembrane receptor protein tyrosine kinase signaling pathway	9	0.0466	2.2553
GO:0048858	cell projection morphogenesis	9	0.0709	2.0620
GO:0032990	cell part morphogenesis	9	0.0862	1.9734
GO:0007584	response to nutrient	8	0.0124	3.2076
GO:0046942	carboxylic acid transport	8	0.0159	3.0548
GO:0015849	organic acid transport	8	0.0164	3.0342
GO:0007548	sex differentiation	8	0.0181	2.9739
GO:0006644	phospholipid metabolic process	8	0.0527	2.3635
GO:0040012	regulation of locomotion	8	0.0552	2.3389

Table S3: Continued

GO ID	GO Term	Count	PValue	Fold Enrichment
GO:0051270	regulation of cell motion	8	0.0564	2.3267
GO:0031667	response to nutrient levels	8	0.0616	2.2795
GO:0051046	regulation of secretion	8	0.0685	2.2231
GO:0048667	cell morphogenesis involved in neuron differentiation	8	0.0789	2.1486
GO:0007517	muscle organ development	8	0.0820	2.1283
GO:0009968	negative regulation of signal transduction	8	0.0987	2.0320
GO:0033273	response to vitamin	7	0.0011	5.9535
GO:0002237	response to molecule of bacterial origin	7	0.0042	4.5689
GO:0051348	negative regulation of transferase activity	7	0.0072	4.0930
GO:0040017	positive regulation of locomotion	7	0.0079	4.0095
GO:0021700	developmental maturation	7	0.0092	3.8904
GO:0042692	muscle cell differentiation	7	0.0208	3.2474
GO:0045137	development of primary sexual characteristics	7	0.0256	3.0939
GO:0032583	regulation of gene-specific transcription	7	0.0322	2.9323
GO:0001525	angiogenesis	7	0.0484	2.6549
GO:0043062	extracellular structure organization	7	0.0704	2.4106
GO:0030335	positive regulation of cell migration	6	0.0210	3.7842
GO:0033673	negative regulation of kinase activity	6	0.0219	3.7422
GO:0001822	kidney development	6	0.0280	3.5083
GO:0051272	positive regulation of cell motion	6	0.0302	3.4367
GO:0051329	interphase of mitotic cell cycle	6	0.0364	3.2699
GO:0051325	interphase	6	0.0404	3.1773
GO:0001655	urogenital system development	6	0.0461	3.0618
GO:0008406	gonad development	6	0.0491	3.0071
GO:0048608	reproductive structure development	6	0.0737	2.6730
GO:0010038	response to metal ion	6	0.0797	2.6108
GO:0022604	regulation of cell morphogenesis	6	0.0838	2.5710
GO:0016055	Wnt receptor signaling pathway	6	0.0881	2.5323
GO:0006470	protein amino acid dephosphorylation	6	0.0881	2.5323
GO:0002252	immune effector process	6	0.0902	2.5134
GO:0051493	regulation of cytoskeleton organization	6	0.0947	2.4764
GO:0030855	epithelial cell differentiation	6	0.0969	2.4584
GO:0048638	regulation of developmental growth	5	0.0095	5.9716
GO:0015718	monocarboxylic acid transport	5	0.0118	5.6133

Table S3: Continued

GO ID	GO Term	Count	PValue	Fold Enrichment
GO:0045444	fat cell differentiation	5	0.0143	5.2955
GO:0009636	response to toxin	5	0.0229	4.6010
GO:0046545	development of primary female sexual characteristics	5	0.0341	4.0676
GO:0046660	female sex differentiation	5	0.0341	4.0676
GO:0031349	positive regulation of defense response	5	0.0406	3.8447
GO:0048469	cell maturation	5	0.0442	3.7422
GO:0032496	response to lipopolysaccharide	5	0.0479	3.6450
GO:0030278	regulation of ossification	5	0.0498	3.5983
GO:0009266	response to temperature stimulus	5	0.0600	3.3815
GO:0010638	positive regulation of organelle organization	5	0.0600	3.3815
GO:0006469	negative regulation of protein kinase activity	5	0.0689	3.2260
GO:0043523	regulation of neuron apoptosis	5	0.0760	3.1185
GO:0042058	regulation of epidermal growth factor receptor signaling pathway	4	0.0075	9.7622
GO:0032675	regulation of interleukin-6 production	4	0.0256	6.2370
GO:0030072	peptide hormone secretion	4	0.0315	5.7572
GO:0002790	peptide secretion	4	0.0359	5.4764
GO:0046879	hormone secretion	4	0.0453	4.9896
GO:0006690	icosanoid metabolic process	4	0.0505	4.7773
GO:0032582	negative regulation of gene-specific transcription	4	0.0532	4.6777
GO:0042593	glucose homeostasis	4	0.0560	4.5823
GO:0009914	hormone transport	4	0.0560	4.5823
GO:0033500	carbohydrate homeostasis	4	0.0560	4.5823
GO:0032886	regulation of microtubule-based process	4	0.0560	4.5823
GO:0033559	unsaturated fatty acid metabolic process	4	0.0617	4.4026
GO:0008277	regulation of G-protein coupled receptor protein signaling pathway	4	0.0617	4.4026
GO:0001889	liver development	4	0.0676	4.2364
GO:0015833	peptide transport	4	0.0707	4.1580
GO:0008037	cell recognition	4	0.0739	4.0824
GO:0000082	G1/S transition of mitotic cell cycle	4	0.0771	4.0095
GO:0045787	positive regulation of cell cycle	4	0.0803	3.9391

Table S3: Continued

GO ID	GO Term	Count	PValue	Fold Enrichment
GO:0007179	transforming growth factor beta receptor signaling pathway	4	0.0905	3.7422
GO:0045410	positive regulation of interleukin-6 biosynthetic process	3	0.0045	28.0664
GO:0043584	nose development	3	0.0082	21.0498
GO:0060324	face development	3	0.0155	15.3089
GO:0030728	ovulation	3	0.0248	12.0285
GO:0045408	regulation of interleukin-6 biosynthetic process	3	0.0248	12.0285
GO:0060322	head development	3	0.0248	12.0285
GO:0007176	regulation of epidermal growth factor receptor activity	3	0.0283	11.2266
GO:0048639	positive regulation of developmental growth	3	0.0319	10.5249
GO:0010469	regulation of receptor activity	3	0.0398	9.3555
GO:0006693	prostaglandin metabolic process	3	0.0440	8.8631
GO:0006692	prostanoid metabolic process	3	0.0440	8.8631
GO:0030516	regulation of axon extension	3	0.0483	8.4199
GO:0051607	defense response to virus	3	0.0483	8.4199
GO:0009409	response to cold	3	0.0528	8.0190
GO:0009395	phospholipid catabolic process	3	0.0574	7.6545
GO:0019827	stem cell maintenance	3	0.0720	6.7359
GO:0032768	regulation of monooxygenase activity	3	0.0720	6.7359
GO:0048146	positive regulation of fibroblast proliferation	3	0.0771	6.4769
GO:0048864	stem cell development	3	0.0771	6.4769
GO:0030073	insulin secretion	3	0.0823	6.2370
GO:0045740	positive regulation of DNA replication	3	0.0877	6.0142
GO:0030199	collagen fibril organization	3	0.0931	5.8068
GO:0030500	regulation of bone mineralization	3	0.0986	5.6133
GO:0021549	cerebellum development	3	0.0986	5.6133
GO:0050919	negative chemotaxis	2	0.0856	22.4531
GO:0051001	negative regulation of nitric-oxide synthase activity	2	0.0856	22.4531
GO:0045741	positive regulation of epidermal growth factor receptor activity	2	0.0856	22.4531
Cellular componoent				
GO:0005886	plasma membrane	87	0.0040	1.2913

Table S3: Continued

GO ID	GO Term	Count	PValue	Fold Enrichment
GO:0030141	secretory granule	10	0.0049	3.1145
GO:0000267	cell fraction	32	0.0054	1.6565
GO:0042995	cell projection	23	0.0064	1.8499
GO:0044459	plasma membrane part	54	0.0108	1.3742
GO:0031982	vesicle	21	0.0160	1.7571
GO:0031091	platelet alpha granule	5	0.0173	5.0055
GO:0005856	cytoskeleton	36	0.0193	1.4614
GO:0031410	cytoplasmic vesicle	20	0.0203	1.7465
GO:0031988	membrane-bounded vesicle	18	0.0249	1.7766
GO:0016323	basolateral plasma membrane	9	0.0286	2.4855
GO:0016023	cytoplasmic membrane-bounded vesicle	17	0.0364	1.7328
GO:0015630	microtubule cytoskeleton	17	0.0364	1.7360
GO:0005626	insoluble fraction	23	0.0429	1.5368
GO:0060205	cytoplasmic membrane-bounded vesicle lumen	4	0.0430	5.0965
GO:0031983	vesicle lumen	4	0.0480	4.8749
GO:0005887	integral to plasma membrane	30	0.0486	1.4157
GO:0030055	cell-substrate junction	6	0.0493	3.0033
GO:0015629	actin cytoskeleton	10	0.0511	2.0841
GO:0005624	membrane fraction	22	0.0516	1.5245
GO:0005625	soluble fraction	11	0.0532	1.9702
GO:0044421	extracellular region part	25	0.0558	1.4599
GO:0005792	microsome	9	0.0611	2.1289
GO:0031226	intrinsic to plasma membrane	30	0.0614	1.3842
GO:0042598	vesicular fraction	9	0.0698	2.0678
GO:0005773	vacuole	9	0.0806	2.0022
GO:0043005	neuron projection	11	0.0844	1.8031
GO:0005853	eukaryotic translation elongation factor 1 complex	2	0.0857	22.4246
GO:0070161	anchoring junction	7	0.0863	2.2816
Molecular Function				
GO:0019207	kinase regulator activity	9	0.0003	5.3465
GO:0019887	protein kinase regulator activity	8	0.0006	5.4565
GO:0047115	trans-1,2-dihydrobenzene-1,2-diol dehydrogenase activity	3	0.0019	41.4351
GO:0031418	L-ascorbic acid binding	4	0.0060	10.5232
GO:0005509	calcium ion binding	28	0.0082	1.6833

Table S3: Continued

GO ID	GO Term	Count	PValue	Fold Enrichment
GO:0008028	monocarboxylic acid transmembrane transporter activity	4	0.0088	9.2078
GO:0019900	kinase binding	9	0.0159	2.7778
GO:0004860	protein kinase inhibitor activity	4	0.0267	6.1385
GO:0005518	collagen binding	4	0.0267	6.1385
GO:0019210	kinase inhibitor activity	4	0.0286	5.9726
GO:0008656	caspase activator activity	3	0.0329	10.3588
GO:0047718	indanol dehydrogenase activity	2	0.0357	55.2468
GO:0047026	3-alpha-hydroxysteroid dehydrogenase (A-specific) activity	2	0.0357	55.2468
GO:0030291	protein serine/threonine kinase inhibitor activity	3	0.0368	9.7494
GO:0008092	cytoskeletal protein binding	16	0.0395	1.7539
GO:0016628	oxidoreductase activity, acting on the CH-CH group of donors, NAD or NADP as acceptor	3	0.0452	8.7232
GO:0050662	coenzyme binding	8	0.0457	2.4418
GO:0031406	carboxylic acid binding	7	0.0461	2.6856
GO:0051287	NAD or NADH binding	4	0.0525	4.7019
GO:0030145	manganese ion binding	7	0.0601	2.5112
GO:0016651	oxidoreductase activity, acting on NADH or NADPH	5	0.0628	3.3281
GO:0016504	peptidase activator activity	3	0.0639	7.2061
GO:0008289	lipid binding	14	0.0641	1.7188
GO:0032403	protein complex binding	8	0.0646	2.2550
GO:0003746	translation elongation factor activity	3	0.0689	6.9059
GO:0005088	Ras guanyl-nucleotide exchange factor activity	5	0.0745	3.1390
GO:0004721	phosphoprotein phosphatase activity	7	0.0782	2.3438
GO:0048037	cofactor binding	9	0.0818	1.9969
GO:0008047	enzyme activator activity	11	0.0819	1.8141
GO:0033764	steroid dehydrogenase activity, acting on the CH-OH group of donors, NAD or NADP as acceptor	3	0.0846	6.1385
GO:0019842	vitamin binding	6	0.0860	2.5499
GO:0004862	cAMP-dependent protein kinase inhibitor activity	2	0.0869	22.0987
GO:0004791	thioredoxin-disulfide reductase activity	2	0.0869	22.0987

Table S3: Continued

GO ID	GO Term	Count	PValue	Fold Enrichment
GO:0005522	profilin binding	2	0.0869	22.0987
GO:0015355	secondary active monocarboxylate transmembrane transporter activity	2	0.0869	22.0987
GO:0030674	protein binding, bridging	5	0.0898	2.9387
GO:0005158	insulin receptor binding	3	0.0901	5.9193
GO:0016706	oxidoreductase activity, acting on paired donors, with incorporation or reduction of molecular oxygen, 2-oxoglutarate as one donor, and incorporation of one atom each of oxygen into both donors	3	0.0901	5.9193
GO:0043028	caspase regulator activity	3	0.0956	5.7152
