# Supplementary material for: Anticancer Agent Shikonin Is an Incompetent Inducer of Cancer Drug Resistance
Source: PLoS One. 2013 Jan 3;8(1):e52706. doi: 10.1371/journal.pone.0052706 (PMC3536779; doi:10.1371/journal.pone.0052706)
Supplement: Table S4 — The common gene ontology terms of down regulated genes in K562/Shk cells. Cells were treated with shikonin for 18 months, control cells were treated with vehicle as described in Materials and Methods. (PDF) [file pone.0052706.s004.pdf]

Table S4: The common gene ontology terms of down regulated genes in K562/Shk cells. Cells were treated with shikonin for 18 months, control cells were treated with vehicle as described in Materials and Methods.

GO ID	GO Term	Count	PValue	Fold Enrichment
Biological Process				
GO:0030036	actin cytoskeleton organization	14	0.0307	1.9265
GO:0051693	actin filament capping	4	0.0321	5.6543
GO:0030029	actin filament-based process	14	0.0478	1.8066
GO:0007257	activation of JUN kinase activity	5	0.0089	5.9805
GO:0006639	acylglycerol metabolic process	6	0.0199	3.8080
GO:0006916	anti-apoptosis	12	0.0687	1.8116
GO:0006915	apoptosis	31	0.0114	1.6014
GO:0048318	axial mesoderm development	2	0.0932	20.7326
GO:0007610	behavior	23	0.0484	1.5251
GO:0046394	carboxylic acid biosynthetic process	10	0.0621	2.0064
GO:0009712	catechol metabolic process	4	0.0945	3.6587
GO:0006584	catecholamine metabolic process	4	0.0945	3.6587
GO:0008219	cell death	37	0.0054	1.6004
GO:0030031	cell projection assembly	9	0.0051	3.3722
GO:0030030	cell projection organization	20	0.0286	1.6902
GO:0032989	cellular component morphogenesis	20	0.0541	1.5667
GO:0006073	cellular glucan metabolic process	6	0.0055	5.1831
GO:0006879	cellular iron ion homeostasis	4	0.0760	4.0128
GO:0044264	cellular polysaccharide metabolic process	6	0.0270	3.5206
GO:0033554	cellular response to stress	25	0.0998	1.3736
GO:0042632	cholesterol homeostasis	5	0.0326	4.0920
GO:0007010	cytoskeleton organization	31	0.0001	2.2112
GO:0016265	death	37	0.0059	1.5893
GO:0034311	diol metabolic process	4	0.0945	3.6587
GO:0006259	DNA metabolic process	23	0.0918	1.4136
GO:0006270	DNA replication initiation	3	0.0917	5.8310
GO:0006897	endocytosis	13	0.0518	1.8377
GO:0006112	energy reserve metabolic process	6	0.0117	4.3394
GO:0006631	fatty acid metabolic process	13	0.0261	2.0418
GO:0044042	glucan metabolic process	6	0.0055	5.1831
GO:0006006	glucose metabolic process	12	0.0103	2.4391
GO:0009064	glutamine family amino acid metabolic process	5	0.0849	2.9903
GO:0006662	glycerol ether metabolic process	6	0.0233	3.6587
GO:0046486	glycerolipid metabolic process	10	0.0772	1.9197

Table S4: Continued

GO ID	GO Term	Count	PValue	Fold Enrichment
GO:0005977	glycogen metabolic process	6	0.0049	5.3312
GO:0019318	hexose metabolic process	16	0.0013	2.5916
GO:0048872	homeostasis of number of cells	8	0.0417	2.4879
GO:0002520	immune system development	15	0.0619	1.6902
GO:0008624	induction of apoptosis by extracellular signals	8	0.0687	2.2213
GO:0007242	intracellular signaling cascade	54	0.0231	1.3371
GO:0006826	iron ion transport	4	0.0647	4.2895
GO:0007254	JNK cascade	7	0.0113	3.6897
GO:0045321	leukocyte activation	13	0.0906	1.6706
GO:0055088	lipid homeostasis	6	0.0233	3.6587
GO:0010876	lipid localization	10	0.0662	1.9808
GO:0006869	lipid transport	9	0.0951	1.9303
GO:0046649	lymphocyte activation	12	0.0561	1.8753
GO:0051235	maintenance of location	7	0.0165	3.4014
GO:0051651	maintenance of location in cell	5	0.0673	3.2395
GO:0000165	MAPKKK cascade	12	0.0351	2.0282
GO:0010324	membrane invagination	13	0.0518	1.8377
GO:0001765	membrane raft formation	2	0.0631	31.0989
GO:0031579	membrane raft organization	3	0.0194	13.3281
GO:0000226	microtubule cytoskeleton organization	10	0.0473	2.1156
GO:0005996	monosaccharide metabolic process	17	0.0022	2.3814
GO:0030835	negative regulation of actin filament depolymerization	4	0.0403	5.1831
GO:0030837	negative regulation of actin filament polymerization	5	0.0116	5.5534
GO:0043066	negative regulation of apoptosis	18	0.0647	1.5813
GO:0060548	negative regulation of cell death	18	0.0730	1.5549
GO:0030336	negative regulation of cell migration	6	0.0355	3.2736
GO:0051271	negative regulation of cell motion	6	0.0512	2.9618
GO:0008285	negative regulation of cell proliferation	19	0.0437	1.6368
GO:0051129	negative regulation of cellular component organization	11	0.0161	2.4091
GO:0051494	negative regulation of cytoskeleton organization	6	0.0311	3.3926
GO:0010888	negative regulation of lipid storage	3	0.0548	7.7747
GO:0040013	negative regulation of locomotion	6	0.0456	3.0589

Table S4: Continued

GO ID	GO Term	Count	PValue	Fold Enrichment
GO:0045665	negative regulation of neuron differentiation	4	0.0882	3.7696
GO:0010639	negative regulation of organelle organization	7	0.0479	2.6548
GO:0043069	negative regulation of programmed cell death	18	0.0716	1.5593
GO:0031333	negative regulation of protein complex assembly	6	0.0043	5.4880
GO:0043242	negative regulation of protein complex disassembly	5	0.0416	3.7925
GO:0032272	negative regulation of protein polymerization	5	0.0131	5.3619
GO:0006638	neutral lipid metabolic process	7	0.0051	4.3538
GO:0044271	nitrogen compound biosynthetic process	17	0.0600	1.6267
GO:0009116	nucleoside metabolic process	6	0.0541	2.9155
GO:0009124	nucleoside monophosphate biosynthetic process	5	0.0758	3.1099
GO:0070427	nucleotide-binding oligomerization domain containing 1 signaling pathway	2	0.0631	31.0989
GO:0016053	organic acid biosynthetic process	10	0.0621	2.0064
GO:0018904	organic ether metabolic process	6	0.0270	3.5206
GO:0006796	phosphate metabolic process	45	0.0130	1.4383
GO:0006817	phosphate transport	3	0.0634	7.1767
GO:0006793	phosphorus metabolic process	45	0.0130	1.4383
GO:0016310	phosphorylation	36	0.0373	1.3994
GO:0046635	positive regulation of alpha-beta T cell activation	4	0.0494	4.7844
GO:0046641	positive regulation of alpha-beta T cell proliferation	3	0.0318	10.3663
GO:0043065	positive regulation of apoptosis	22	0.0373	1.5911
GO:0010647	positive regulation of cell communication	17	0.0656	1.6069
GO:0010942	positive regulation of cell death	22	0.0412	1.5728
GO:0032729	positive regulation of interferon-gamma production	3	0.0466	8.4815

Table S4: Continued

GO ID	GO Term	Count	PValue	Fold Enrichment
GO:0043507	positive regulation of JUN kinase activity	6	0.0033	5.8310
GO:0070665	positive regulation of leukocyte proliferation	6	0.0333	3.3320
GO:0051251	positive regulation of lymphocyte activation	7	0.0915	2.2442
GO:0050671	positive regulation of lymphocyte proliferation	6	0.0311	3.3926
GO:0032946	positive regulation of mononuclear cell proliferation	6	0.0333	3.3320
GO:0043068	positive regulation of programmed cell death	22	0.0398	1.5801
GO:0009967	positive regulation of signal transduction	15	0.0942	1.5813
GO:0050870	positive regulation of T cell activation	7	0.0350	2.8644
GO:0002842	positive regulation of T cell mediated immune response to tumor cell	2	0.0631	31.0989
GO:0042102	positive regulation of T cell proliferation	5	0.0355	3.9870
GO:0009791	post-embryonic development	6	0.0849	2.5561
GO:0012501	programmed cell death	32	0.0080	1.6287
GO:0046777	protein amino acid autophosphorylation	8	0.0192	2.9270
GO:0006468	protein amino acid phosphorylation	34	0.0087	1.5852
GO:0051260	protein homooligomerization	8	0.0329	2.6189
GO:0007243	protein kinase cascade	22	0.0084	1.8491
GO:0032956	regulation of actin cytoskeleton organization	7	0.0662	2.4460
GO:0030834	regulation of actin filament depolymerization	4	0.0543	4.6072
GO:0030832	regulation of actin filament length	6	0.0512	2.9618
GO:0030833	regulation of actin filament polymerization	6	0.0290	3.4554
GO:0032970	regulation of actin filament-based process	7	0.0752	2.3662
GO:0008064	regulation of actin polymerization or depolymerization	6	0.0456	3.0589
GO:0046640	regulation of alpha-beta T cell proliferation	3	0.0634	7.1767
GO:0042981	regulation of apoptosis	37	0.0268	1.4312

Table S4: Continued

GO ID	GO Term	Count	PValue	Fold Enrichment
GO:0030155	regulation of cell adhesion	10	0.0324	2.2700
GO:0010941	regulation of cell death	37	0.0318	1.4118
GO:0033632	regulation of cell-cell adhesion mediated by integrin	2	0.0932	20.7326
GO:0044087	regulation of cellular component biogenesis	14	0.0006	3.0661
GO:0080135	regulation of cellular response to stress	8	0.0477	2.4154
GO:0000079	regulation of cyclin-dependent protein kinase activity	7	0.0074	4.0313
GO:0051493	regulation of cytoskeleton organization	10	0.0311	2.2867
GO:0045598	regulation of fat cell differentiation	3	0.0917	5.8310
GO:0046328	regulation of JNK cascade	7	0.0177	3.3491
GO:0043506	regulation of JUN kinase activity	6	0.0062	5.0431
GO:0043549	regulation of kinase activity	23	0.0027	2.0036
GO:0043405	regulation of MAP kinase activity	11	0.0154	2.4262
GO:0043408	regulation of MAPKKK cascade	10	0.0084	2.8531
GO:0033043	regulation of organelle organization	15	0.0104	2.1497
GO:0019220	regulation of phosphate metabolic process	25	0.0242	1.6030
GO:0051174	regulation of phosphorus metabolic process	25	0.0242	1.6030
GO:0042325	regulation of phosphorylation	25	0.0155	1.6684
GO:0043067	regulation of programmed cell death	37	0.0304	1.4171
GO:0043254	regulation of protein complex assembly	11	0.0006	3.8010
GO:0043244	regulation of protein complex disassembly	5	0.0803	3.0489
GO:0045859	regulation of protein kinase activity	22	0.0039	1.9831
GO:0010627	regulation of protein kinase cascade	16	0.0147	1.9983
GO:0032271	regulation of protein polymerization	8	0.0060	3.6587
GO:0051056	regulation of small GTPase mediated signal transduction	14	0.0633	1.7277
GO:0070302	regulation of stress-activated protein kinase signaling pathway	8	0.0065	3.6057
GO:0002840	regulation of T cell mediated immune response to tumor cell	2	0.0631	31.0989
GO:0051338	regulation of transferase activity	23	0.0044	1.9228
GO:0060627	regulation of vesicle-mediated transport	7	0.0881	2.2676

Table S4: Continued

GO ID	GO Term	Count	PValue	Fold Enrichment
GO:0042493	response to drug	12	0.0891	1.7277
GO:0043627	response to estrogen stimulus	8	0.0519	2.3694
GO:0055092	sterol homeostasis	5	0.0326	4.0920
GO:0031098	stress-activated protein kinase signaling pathway	7	0.0153	3.4554
GO:0048488	synaptic vesicle endocytosis	3	0.0725	6.6640
GO:0042110	T cell activation	10	0.0201	2.4682
GO:0034142	toll-like receptor 4 signaling pathway	2	0.0631	31.0989
GO:0002224	toll-like receptor signaling pathway	3	0.0725	6.6640
GO:0006641	triglyceride metabolic process	6	0.0117	4.3394
GO:0016050	vesicle organization	5	0.0849	2.9903
GO:0006766	vitamin metabolic process	6	0.0928	2.4879
Cellular componoent				
GO:0019867	outer membrane	11	0.0014	3.4260
GO:0005829	cytosol	59	0.0017	1.4922
GO:0005739	mitochondrion	49	0.0034	1.5163
GO:0031968	organelle outer membrane	10	0.0037	3.2343
GO:0030665	clathrin coated vesicle membrane	7	0.0046	4.4426
GO:0005741	mitochondrial outer membrane	9	0.0052	3.3637
GO:0031090	organelle membrane	48	0.0064	1.4731
GO:0019898	extrinsic to membrane	26	0.0066	1.7704
GO:0045177	apical part of cell	12	0.0178	2.2550
GO:0030662	coated vesicle membrane	7	0.0210	3.2255
GO:0000139	Golgi membrane	12	0.0229	2.1701
GO:0031430	M band	3	0.0275	11.2123
GO:0015629	actin cytoskeleton	15	0.0298	1.8757
GO:0012506	vesicle membrane	10	0.0359	2.2276
GO:0005905	coated pit	5	0.0407	3.8224
GO:0005856	cytoskeleton	53	0.0414	1.2909
GO:0005740	mitochondrial envelope	20	0.0438	1.6056
GO:0031966	mitochondrial membrane	19	0.0462	1.6221
GO:0030135	coated vesicle	10	0.0473	2.1155
GO:0001726	ruffle	6	0.0483	3.0123
GO:0009898	internal side of plasma membrane	16	0.0495	1.7031
GO:0044433	cytoplasmic vesicle part	11	0.0520	1.9786
GO:0000228	nuclear chromosome	10	0.0521	2.0763
GO:0030659	cytoplasmic vesicle membrane	9	0.0549	2.1779

Table S4: Continued

GO ID	GO Term	Count	PValue	Fold Enrichment
GO:0034362	low-density lipoprotein particle	3	0.0551	7.7623
GO:0012505	endomembrane system	32	0.0597	1.3764
GO:0000123	histone acetyltransferase complex	5	0.0603	3.3637
GO:0005657	replication fork	4	0.0681	4.2046
GO:0043292	contractile fiber	8	0.0684	2.2239
GO:0005813	centrosome	12	0.0699	1.8020
GO:0005811	lipid particle	3	0.0714	6.7274
GO:0031967	organelle envelope	26	0.0737	1.4106
GO:0031975	envelope	26	0.0766	1.4060
GO:0031672	A band	3	0.0800	6.3069
GO:0000793	condensed chromosome	8	0.0891	2.0860
GO:0031982	vesicle	27	0.0962	1.3555
GO:0030136	clathrin-coated vesicle	8	0.0977	2.0386
Molecular Function				
GO:0030695	GTPase regulator activity	27	0.0006	2.0708
GO:0060589	nucleoside-triphosphatase regulator activity	27	0.0009	2.0257
GO:0005083	small GTPase regulator activity	19	0.0034	2.1486
GO:0004672	protein kinase activity	33	0.0041	1.6873
GO:0000287	magnesium ion binding	26	0.0060	1.7824
GO:0008047	enzyme activator activity	21	0.0060	1.9424
GO:0008092	cytoskeletal protein binding	28	0.0068	1.7214
GO:0004674	protein serine/threonine kinase activity	23	0.0220	1.6574
GO:0046914	transition metal ion binding	108	0.0223	1.2016
GO:0005085	guanyl-nucleotide exchange factor activity	11	0.0252	2.2424
GO:0046872	metal ion binding	153	0.0270	1.1451
GO:0043169	cation binding	154	0.0290	1.1419
GO:0016563	transcription activator activity	21	0.0429	1.5871
GO:0043167	ion binding	154	0.0463	1.1252
GO:0005524	ATP binding	60	0.0468	1.2587
GO:0016410	N-acyltransferase activity	7	0.0510	2.6133
GO:0005096	GTPase activator activity	13	0.0527	1.8310
GO:0032559	adenyl ribonucleotide binding	60	0.0576	1.2419
GO:0001882	nucleoside binding	64	0.0583	1.2302
GO:0008270	zinc ion binding	88	0.0589	1.1799

Table S4: Continued

GO ID	GO Term	Count	PValue	Fold Enrichment
GO:0005088	Ras guanyl-nucleotide exchange factor activity	7	0.0642	2.4648
GO:0001883	purine nucleoside binding	63	0.0691	1.2193
GO:0008080	N-acetyltransferase activity	6	0.0711	2.6944
GO:0030554	adenyl nucleotide binding	62	0.0719	1.2182
GO:0032553	ribonucleotide binding	71	0.0723	1.1982
GO:0032555	purine ribonucleotide binding	71	0.0723	1.1982
GO:0004033	aldo-keto reductase activity	3	0.0824	6.1971
GO:0017076	purine nucleotide binding	73	0.0873	1.1793
GO:0005089	Rho guanyl-nucleotide exchange factor activity	6	0.0898	2.5124
GO:0051015	actin filament binding	5	0.0905	2.9232
GO:0008289	lipid binding	21	0.0908	1.4460
GO:0005173	stem cell factor receptor binding	2	0.0935	20.6571
GO:0015226	carnitine transporter activity	2	0.0935	20.6571
GO:0033293	monocarboxylic acid binding	5	0.0954	2.8690
